# Do Ionic Liquids Exhibit the Required Characteristics to Dissolve, Extract, Stabilize, and Purify Proteins? Past-Present-Future Assessment

**DOI:** 10.1021/acs.chemrev.3c00551

**Published:** 2024-03-04

**Authors:** Pankaj Bharmoria, Alesia A. Tietze, Dibyendu Mondal, Tejwant Singh Kang, Arvind Kumar, Mara G Freire

**Affiliations:** † CICECO - Aveiro Institute of Materials, Chemistry Department, University of Aveiro, 3810-193 Aveiro, Portugal; ‡ Department of Smart Molecular, Inorganic and Hybrid Materials, Institute of Materials Science of Barcelona (ICMAB-CSIC), 08193 Bellaterra, Barcelona, Spain; § Department of Chemistry and Molecular Biology, Wallenberg Centre for Molecular and Translational Medicine, University of Gothenburg, SE-412 96 Göteborg, Sweden; ∥ Institute of Plant Genetics (IPG), Polish Academy of Sciences, Strzeszyńska 34, 60-479 Poznań, Poland; ⊥ Centre for Nano and Material Sciences, JAIN (Deemed-to-be University), Jain Global Campus, Bangalore 562112, India; # Department of Chemistry, UGC Center for Advance Studies-II, Guru Nanak Dev University (GNDU), Amritsar 143005, Punjab, India; ● Salt and Marine Chemicals Division, CSIR-Central Salt and Marine Chemicals Research Institute, G. B. Marg, Bhavnagar 364002, Gujarat, India

## Abstract

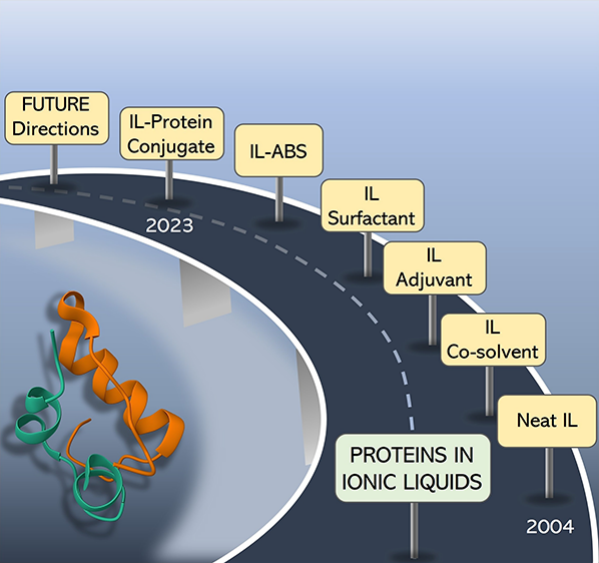

Proteins are highly labile molecules, thus requiring the presence of appropriate solvents and excipients in their liquid milieu to keep their stability and biological activity. In this field, ionic liquids (ILs) have gained momentum in the past years, with a relevant number of works reporting their successful use to dissolve, stabilize, extract, and purify proteins. Different approaches in protein-IL systems have been reported, namely, proteins dissolved in (*i*) neat ILs, (*ii*) ILs as co-solvents, (*iii*) ILs as adjuvants, (*iv*) ILs as surfactants, (*v*) ILs as phase-forming components of aqueous biphasic systems, and (*vi*) IL-polymer-protein/peptide conjugates. Herein, we critically analyze the works published to date and provide a comprehensive understanding of the IL-protein interactions affecting the stability, conformational alteration, unfolding, misfolding, and refolding of proteins while providing directions for future studies in view of imminent applications. Overall, it has been found that the stability or purification of proteins by ILs is bispecific and depends on the structure of both the IL and the protein. The most promising IL-protein systems are identified, which is valuable when foreseeing market applications of ILs, e.g., in “protein packaging” and “detergent applications”. Future directions and other possibilities of IL-protein systems in light-harvesting and biotechnology/biomedical applications are discussed.

## 1 Introduction

Proteins are among the most incredible creations of nature, performing activities from generation to operation, from the beginning to the end of life.^1^ Their *in vivo* operation has inspired their *ex vivo* applications, such as in healthcare, food,^2,3^ paper pulp bleaching,^4^ leather processing,^5^ biocatalysis,^6^ detergency,^7^ and materials.^8^ However, these applications require proteins in a bioactive form, particularly if storage is envisaged for long periods. Several strategies for the *in vitro* packaging of proteins have been developed,^9−11^ such as protein lyophilization with disaccharides; maintenance of their conformational stability in water using salts, osmolytes, or buffers; ligand binding; and interfacial stabilization promoted by surfactants, colloidal stabilization, aerogels, protein cohabitation, pharmacological chaperones, chemical modification, site-directed mutagenesis, protein staple, and use of ionic liquids (ILs).^9−11^ Among the several strategies investigated, in the past two decades ILs have been a hot topic of research in the field of protein stabilization, dissolution, extraction, and purification.

ILs are salts with an organic cation and organic/inorganic anions, thus having a low lattice energy and low melting points when compared to inorganic salts. Due to their organic nature, ILs are able to establish a wider range of interactions when compared to Coulombic-dominated salts,^12^ resulting in a series of unique properties. Aprotic ILs, if properly designed, have a negligible vapor pressure at ambient conditions and a high thermal and chemical stability. However, one of their most relevant properties is their designer solvent ability, i.e., solvation customization by choosing suitable cation–anion combinations,^13^ which has contributed to the observed continuous growth in IL applications.^14^ These include protein dissolution, stabilization, extraction, and purification in native ILs and their mixtures with molecular solvents.^15−17^

The ability of ILs and their water mixtures to solvate and to keep the protein’s integrity depends on their physicochemical properties, such as viscosity, polarity, hydrogen-bond donor or acceptor ability, ionicity or ion charge density, and IL concentration, among other factors.^18−27^ Neat ILs can exist as ion-pairs, directional hydrogen bond networks, ion-clusters, and/or self-assembled nanostructures,^27^ and all possibilities have a relevant impact on protein solvation and stabilization. In addition to the IL characteristics, the proteins structure, molecular weight, isoelectric point, and conformation are key determinants for their solubility and stability in given ILs. If we apply the “like dissolves like” concept, most proteins are expected to display similar solubility in neat ILs since both exhibit polar/nonpolar domains; yet, this is only true for a few proteins (Lipase, Cytochrome c, Cellulase, Zein, Keratin) and in scarce ILs.^28−34^ Therefore, both the IL and protein contributions should be taken into account when dealing with neat ILs as promising solvents for the molecular packaging of proteins.

Beyond molecular packaging, protein dispersions in neat ILs have been applied to carry out high-temperature biocatalysis.^35,36^ Most reports to date on neat IL-protein systems are focused on enzyme biocatalysis, a topic that has been comprehensively reviewed from 2002 to 2021.^15,37−52^ Because this is beyond the scope of this review, it is here only briefly discussed. These reviews summarized developments in enzyme biocatalysis, achieved mainly with imidazolium-based ILs, highlighting key impediments for practical applications, like high-end cost, the toxicity of the ILs employed, and poor fundamental understanding of IL-enzyme interactions to increase stability and activity of the enzyme. Commonly, less viscous, more hydrophobic, and surface-active ILs, or ILs composed of “kosmotropic” anions and “chaotropic” cations (in accordance with the Hofmeister series^53^), enhance the activity and stability of enzymes.^49^ Overall, the thermal stability of ILs is the key property being exploited in biocatalysis, allowing the increase of the reaction rate by causing more collisions between the enzyme and the substrate at high temperatures. In biocatalysis, enzymes need not be dissolved in ILs and can function in their dispersed form if the active sites are accessible.

As stated before, neat ILs exist in hydrogen-bonded supramolecular structures consisting of polar/nonpolar domains.^24,54^ Upon small dilution with water, marked changes in the physicochemical properties of ILs occur,^55,56^ which then affect their ability to solubilize and stabilize proteins.^57−75^ The main advantage of aqueous concentrated-IL solutions (A-ILCSs) for protein packaging is based on the inherent natural environment, in the form of the surrounding water, in addition to the thermal stability afforded by the presence of IL clusters.^27,54,76^ Nevertheless, opposite findings have been reported, namely on the deleterious effects of ILs on proteins when moving from neat ILs (NILs) to A-ILCS.^57,77^ Furthermore, in highly diluted solutions, IL ions are expected to behave similarly to conventional inorganic electrolytes; however, inconsistent perceptions have been reported,^78−83^ in which the associative or dissociative nature of ILs in dilute solutions seems to depend on their hydrophobicity.^79^ For instance, by molecular dynamics studies, water molecules were reported to be confined at the boundary of the polar and nonpolar of ([C_8_mim][NO_3_])/water mixtures, wherein the IL retained its nanodomain structure.^81^ In contrast, in a later report,^83^ carried out with ^1^H NMR studies with quaternary ammonium- and imidazolium-based ILs, water molecules have been shown to be in close proximity and to be confined inside the IL. These inconsistencies further have implications on how ILs and their solutions with other solvents behave and interact with proteins.

When considering the dilute solution of ILs, attempts have been carried out to better understand the effects of ILs on proteins’ stability based on the Hofmeister series and related concepts.^84−86^ According to the Hofmeister concept, more hydrating (“kosmotrope”) anions, like SO_4_^2–^ and CO_3_^2–^, and less hydrating (“chaotrope”) cations, like NH_4_^+^ and (CH_3_)_4_N^+^, stabilize proteins in aqueous solution, whereas the opposite effect is observed for “kosmotropic” cations (Li^+^ and Mg^2^^+^) and “chaotropic” anions (SCN^–^ and ClO_4_^–^).^87−102^ It should be noted that caution should be taken with the “kosmotropic”/“chaotropic” concepts, which are based on the stabilization or destabilization of the water structure.^103^ In this field, recent spectroscopic and computer simulation studies have refuted these concepts and tried to justify protein stabilization or destabilization based on the ion-specific interactions of salts with the protein surface rather than by ion–water interactions.^97−102^ In-depth discussion on the Hofmeister series and its validity based on the “kosmotropic”/“chaotropic” concept is done later in this review. Most of the reported cations of ILs are “chaotropes”, an inherent result of their large organic cation structure. Accordingly, their Hofmeister-based effect on proteins has been scrutinized by considering ion pairs of “chaotropic” cations and “chaotropic” anions or “chaotropic” cations and “kosmotropic” anions. Interestingly, in addition to the traditional specific ion–water interaction phenomenon, the Hofmeister effect of ILs on proteins has been interpreted in the purview of modifying the protein–water interactions, IL-protein interactions, and the effect of IL cations and anions on protein stability and activity.^15,16,86^ However, contradictions persist to universalize the Hofmeister ranking of IL ions, which is mainly due to the contrasting results obtained with different proteins, thus reinforcing the need for further and systematic revision in the field.

When considering the concept of protein stability, the common perception is that the native conformation of a given protein is thermodynamically the most stable and consequently the most biologically active one.^104^ However, whether the thermodynamically most stable form is the catalytically most active one is still a disputed point.^105^ This is because it is determined by the totality of interatomic interactions and hence by the amino acid sequences.^106−109^ Regarding the thermodynamic vs kinetic control of protein stability in ILs, when proteins are packaged for a long time, thermodynamic control plays a key role since kinetic control depends solely on initial conditions. When short periods are considered instead, for example in biocatalysis, the protein stability is under kinetic control. This is supported by the fact that certain ILs lead to an increase in the activity of enzymes, though their structures are different from their native conformation. ILs provide enough kinetic energy in the system to cross the energy barrier to achieve the most active conformation, which is not at the global energy minima.^110^

When considering ILs composed of hydrophobic/fluorinated anions, such as [TfO]^−^, [Tf_2_N]^−^, and [PF_6_]^−^_,_ it has been shown that local hydrophobic interactions play a major role in protein stability.^16^ On the other hand, when increasing the hydrophobicity of ILs by increasing the alkyl side chain length of either the IL cation or anion, different behaviors upon the IL ion’s interaction with proteins have been identified.^111−140^ ILs with an alkyl side chain length with more than 8 carbon atoms (*n*) have been classified as surface-active ILs (SAILs).^141^ Some SAILs exhibit superior properties compared to their nonsurface-active counterparts in terms of adsorption behavior, emulsifying tendency, and low aggregation concentration.^141,142^ Since surfactants exhibit wide applications with proteins,^143,144^ specifically in detergent and pharma industries, SAIL-protein colloidal formulations were also studied in light of their possible applications in these directions, and they are discussed in a separate section in this review.^111−140,145−149^ In addition to these systems, investigations on proteins fibrillation, PEGylation, and the formation of protein/peptide–polymer conjugates as ways of improving the solubility and stability of proteins in NILs, either for nonaqueous biocatalysis or kinetic storage, have also been discussed.^150−153^

ILs have been used as well to form liquid two-phase systems with water or organic solvents, while envisioning the development of separation processes for proteins/enzymes.^154,155^ Among these, IL-based aqueous biphasic systems (IL-ABS, ternary systems typically composed of water, ILs, and salts) hold advantages over other systems based on more hydrophobic ILs due to their large water content, a beneficial aspect when dealing with most proteins. Therefore, progress made with IL-ABS in pursuit of protein extraction processes^156−158^ is also reviewed in this work.

Before the inception of the IL era, there were already strategies available for the stabilization and packaging of proteins. Therefore, the first section of this review is focused on providing a rational understanding behind the protein’s stability afforded by more conventional strategies. The stronger and weaker links of these strategies are revealed, and limitations that can be overcome by ILs are discussed. The following section covers IL-protein systems in view of molecular-level mechanisms and interactions established between ILs and proteins, in parallel with those occurring in well-known protein stabilizers to come up with a rational perspective of the ILs’ promise. The last section of the current review provides the main conclusions on the suitability of ILs for different protein-based applications, with special emphasis on protein packaging and purification. Future prospects of ILs are finally provided, including the packaging of proteins, as well as in light-harvesting systems, protein/antibody purification, *in vitro* biomimicking of biological fluids (molecular crowding), and 3-D cell culture, among others.

## 2 Rational Understanding behind Protein Stability

In biological fluids, the native conformation of proteins is stabilized by minerals, sugars, lipids, amino acids, and other biomolecules.^159^ Consequently, some of these molecules have been used to stabilize proteins *in vitro*, employing various strategies and making use of several solvents. Below, we briefly review these strategies and solvents used, aiming to better understand and expose the molecular-level mechanisms responsible for protein stability.

### 2.1 Proteins in Pure Water

**Figure 1:**
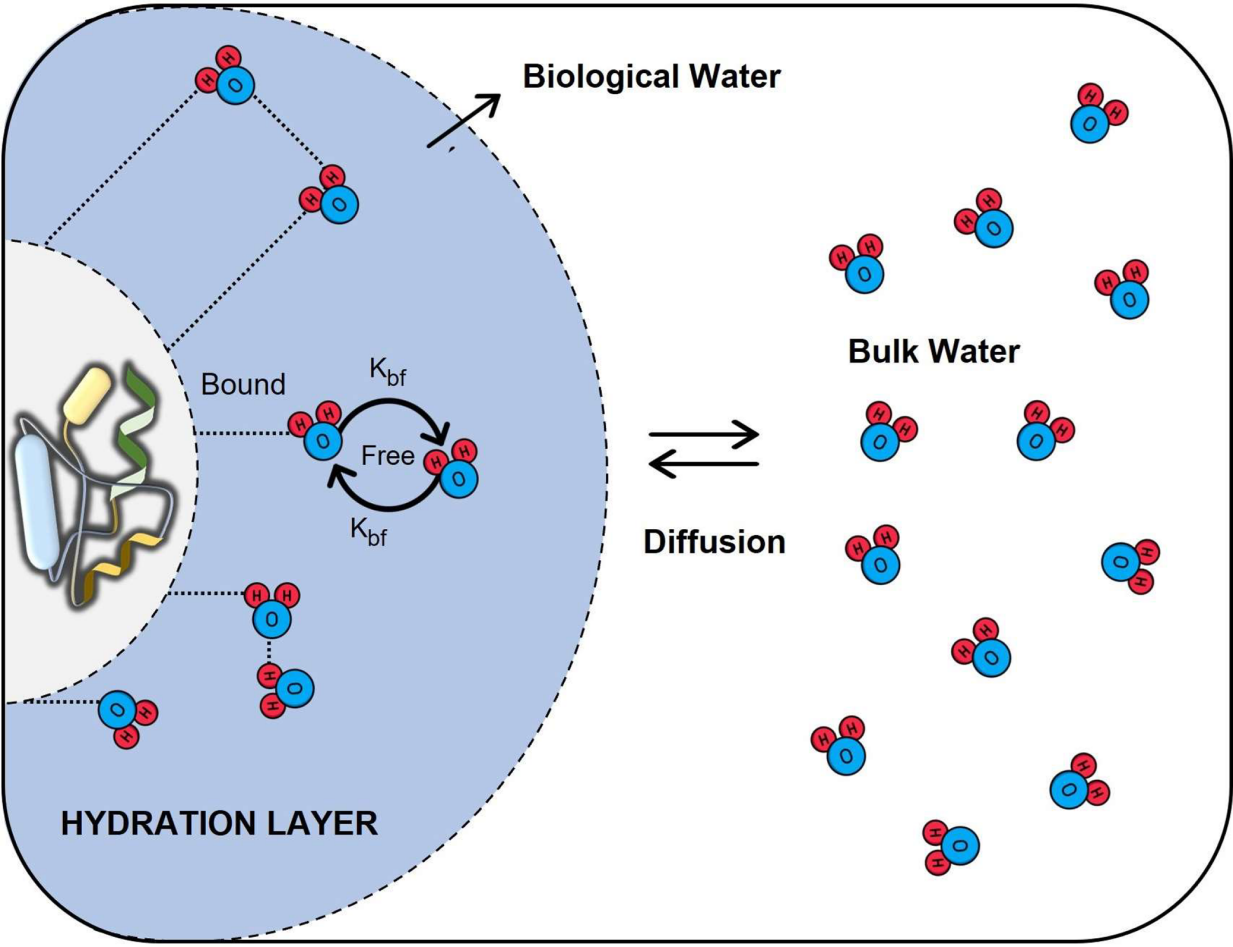
Water molecules in the hydration layer of proteins, showing the “feedback mechanism” of dynamical exchange between free and bound water along with continuous diffusion from the bulk water. Adapted with permission from ref (165). Copyright 2004 American Chemical Society.

The role of water to drive the structure, dynamics, and biological functions of proteins is well-known.^160−172^ Proteins have a high affinity for water, where “about 0.3–0.7 g of bound water remains associated per gram of a dry protein in an aqueous solution.”^160−162^ Upon solubilization in water, proteins adopt a compact structure, driven by H-bonding with the protein-charged sites and hydrophobic solvation of the nonpolar core, where 83% of the protein’s nonpolar side chains and 82% of the peptide groups are buried.^165^ Compared to bulk water, the hydration shell of a protein has been evidenced to be 10–25% denser due to the clustering of water molecules, as determined by translational diffusion coefficients.^160,164^ The hydration process or the cover of the polar charged groups at the protein surface by water assists proteins to attain a high biological activity, sometimes described as a “lubricating effect” on proteins.^167^ The hydration layer of proteins, also called “biological water”,^165^ is composed of bound and free water, which remain in dynamic equilibrium. Free water molecules diffuse into the hydration layer from the bulk, and this represents a “feedback” mechanism of the hydration layer, wherein the bound and free water undergo reversible transitions..^165^ A scheme showing the interactions between water and proteins and the “feedback” mechanism of hydration layer is depicted in Figure 1.

Excellent reviews since the 1950s have highlighted the developments in the understanding of the solvation dynamics of water, in both the hydration shell and bulk, along with the water role in protein stability and activity and protein–protein interactions.^160,163,165,167−170^ Water–protein interactions have been discussed taking into account five main issues: (i) bound water molecules inside a protein, which have shown to provide flexibility to proteins to interact with different strands during folding or collapse;^171^ (ii) presence of water molecules in protein reactions, such as proton transfer in Bacteriorhodopsin (BR) membrane proteins to convert light into chemical energy; (iii) water at the protein surface (biological water), which assists in the stabilization of proteins and acts as an antifreeze agent to sustain life at low temperatures; (iv) solvent role in the protein glass transition, disclosing water–protein interactions and the dynamic transition of free and bulk water; and (v) motions in proteins induced by the translational diffusion of water.^172^

The long-term aqueous stability of proteins at room temperature against aggregation-induced denaturation is still a tricky issue, considering the fact that the native state of proteins is just 2–10 kcal·mol^–1^ more stable than the denatured form.^168^ The major force responsible for protein destabilization is conformational entropy.^173^ Being colloidal solutes, proteins undergo Brownian motion, thus allowing the occurrence of protein–protein interactions that may lead to aggregation and induced denaturation via unfolding pathways. The protein’s stability in water is controlled by five major forces: covalent peptide bonds, covalent disulfide bonds, electrostatic forces, dispersive-type interactions, and H-bonding. However, the total energy contributions of these forces to protein stability are easily overcome by denaturing processes in water, such as deamination, cysteine decomposition, and microbial growth causing denaturation by proteolysis, followed by aggregation. Still, the role of surrounding water in protein denaturation via misfolding and dimerization has also been reported.^174^ These findings reveal the dual nature of biological water in protein functioning and protein misfolding. Although water is the largest fraction of the protein’s biological environment, some proteins, and especially enzymes, have shown higher activity in organic solvents with low water content, with additional advantages, such as higher thermostability and avoidance of denaturation via microbial growth.^174^ Additionally, the stabilization of proteins in water can be further enhanced by the addition of additives (organic solvents, salts, osmolytes, and surfactants) or by their conjugation with polymers, as discussed below.

### 2.2 Proteins in Organic Solvents

Solubility limitations of some important fine chemicals and polymers, the need to reduce unwanted side reactions, and difficulties in product recovery are some of the major reasons behind the need for using organic solvents to solubilize proteins/enzymes.^175^ Organic solvents may additionally offer significant advantages over water, like pH control, protein imprinting, control of substrate specificity, regiospecificity, and enantioselectivity.^175,176^ Upon dispersion in organic solvents, proteins attain conformational rigidity, thus restricting their free mobility. But this does not mean that proteins do not require any water to display enhanced functions in organic solvents. As discussed in section 2.1, proteins attain full biological activity upon the formation of the first hydration layer. Accordingly, an additional question arises on how many water molecules are required. The biological activity of lysozyme is detectable after its hydration with 174 water molecules,^177^ corresponding to the water content required for the protein to acquire mobility, whereas chymotrypsin and subtilisin are active in organic solvents after hydration by 40–60 water molecules,^178^ which corresponds to the hydration of only the protein’s surface polar groups. Also, when lyophilized with specific substrates in water, proteins develop a special affinity for these molecules, even when later dissolved in organic solvents, called “molecular imprinting”.^179,180^ From these results, it is clear that in the absence of water, the protein’s charged groups get locked up with each other leading to an inactive conformation and that water is still required in the presence of organic solvents to maintain the protein stability and improved activity.

Serdakowski et al.^181^ reviewed the published works on enzyme activation in organic solvents in the purview of excipient (sugar and salts) addition during lyophilization. These excipients help in holding the water required to retain enzyme activity after lyophilization processes. They concluded that among sugars, disaccharides, like trehalose and sorbitol, are the best lyoprotectants. However, salts outperformed sugars. For instance, a 3,700-fold increase in the transesterification activity of subtilisin was reported in hexane when the enzyme lyophilization was carried out in the presence of KCl.^182^ Even more remarkably, the penicillin lyophilization in a 1:1 mixture of CsCl and KAc (or CH_3_COOK) leads to a 35,000-fold increase in the enzyme activity in hexane.^183^ The reasons given for such rises in activity were based on the formation of CsAc in solution, comprising a “kosmotropic” anion and a “chaotropic” cation.

Doukyu et al.^184^ reviewed the organic solvent tolerant enzymes and concluded that most of these enzymes are lipases, esterases, and proteases and that their solvent stability depends on the presence of disulfide bonds at their surface and secondary structure. Recently, Stepankova et al.^185^ reviewed the various strategies to stabilize enzymes in organic solvents, which are based on three main methods: (1) isolation of novel enzymes able to function under extreme conditions; (ii) modification of enzyme structures to increase their resistance toward nonconventional solvent media; and (iii) modification of the solvent environment to decrease its denaturing effect on enzymes. Though proteins/enzyme’s stability and activity in organic solvents exhibit certain advantages, this type of application is still limited to a few enzymes, and improvements are still required, either via protein engineering or by the finding of more adequate solvents.

### 2.3 Proteins in Aqueous Solution of Salts

**Figure 2:**
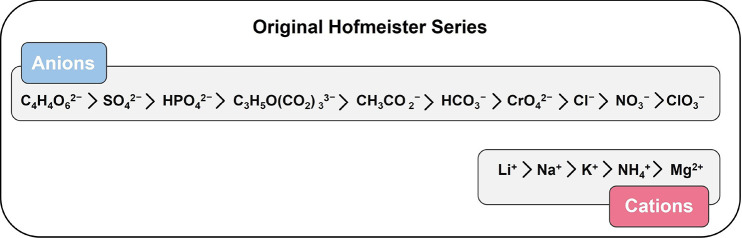
Depiction of the original Hofmeister series published in 1887 by Franz Hofmeister.

It is imperative to understand the behavior of proteins in aqueous solutions of salts owing to their phylogenesis of modulating protein–protein interactions *in vivo* for solubility, aggregation, and function.^186^ However, due to the sheer complexity of salt ion-protein interactions, the related mechanism is still poorly understood.^186−188^ This is partly due to the labyrinthine nano anisotropic colloidal structure of proteins with an inhomogeneous surface charge density and differential polarity.^186,189^ Furthermore, protein–protein interactions involve electrostatic, hydrophobic, ion-dipole, and H-bonding.^190^ Salt ions can modulate these interactions (specifically the electrostatic interactions) subject to their concentrations, hydration, charge, and polarizability.^191,192^ The first important contribution to understanding protein behavior in an aqueous solution of salts was made by Franz Hofmeister.^87^ In 1887, while investigating regularities in the protein precipitating effects of salts and the relation of these effects with the physiological behavior of salts, Hofmeister found that the minimum normal concentration of salts required to precipitate egg globulin from an aqueous solution of egg white proteome follows an ion-specific order, known as the Hofmeister series (HS).^87^ The original HS, published in his 1887 seminal paper, is shown in Figure 2.

**Figure 3:**
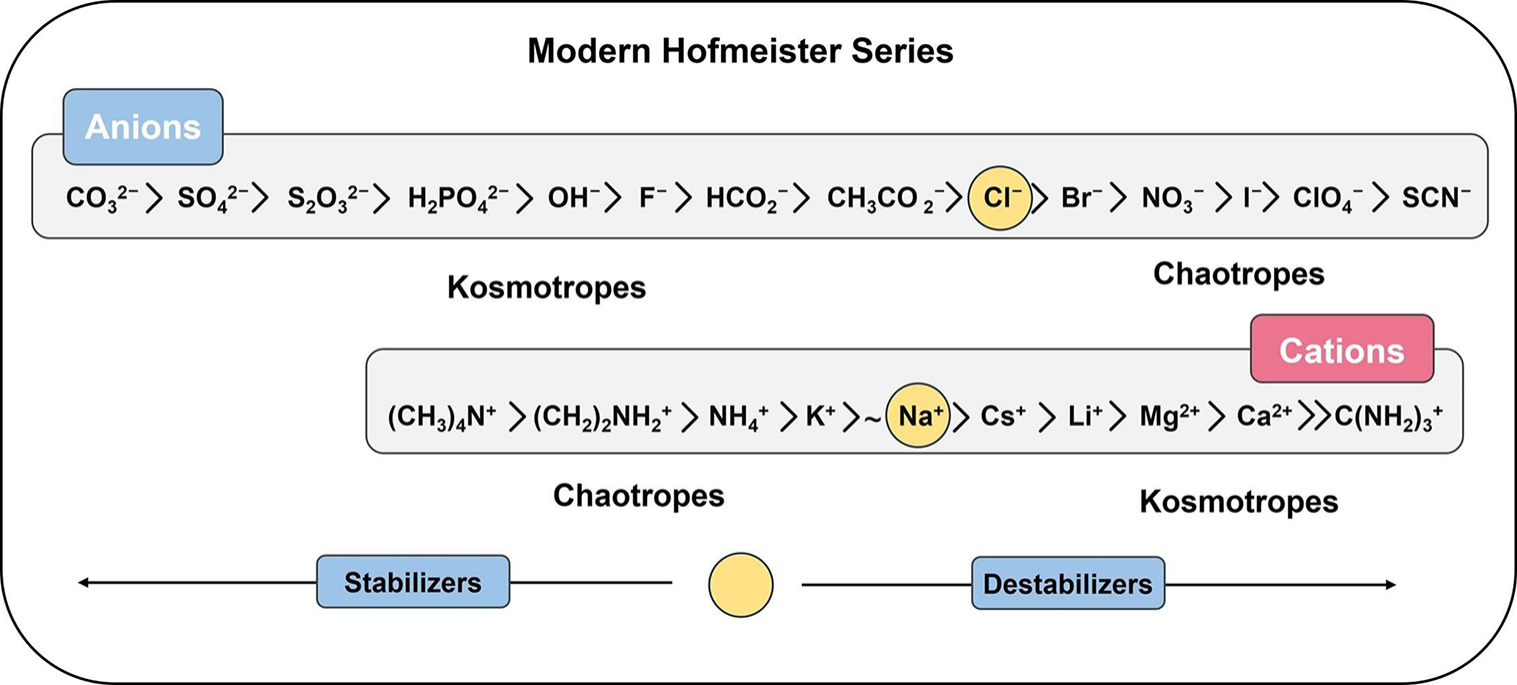
Modern Hofmeister series of ions for protein stability/denaturation.

In his next paper,^88^ Hofmeister explained this effect based on the water-absorbing ability of salts and concluded that more water-absorbing ions, like SO_4_^2^^–^ and Li^+^, induced fast precipitation compared to less water-absorbing ions, like NH_4_^+^ and ClO_3_^–^. This explanation was later framed into the 1930–1950s theory of water structure making (kosmotropes) and breaking (chaotropes) ions.^89,90^ The theory states that strongly hydrating kosmotropes can induce long-range water ordering beyond their first solvation shell, whereas weakly hydrating chaotropes lack this ability.^89−91^ Consequently, kosmotropes can easily withdraw water from the hydration shell of proteins to induce salting-out by promoting van der Waal’s and hydrophobic association of proteins at low concentrations, whereas chaotropes do not. Over the years, further experiments with a number of proteins and salts rendered improvements in the HS and extended the Hofmeister effect to protein stabilization and denaturing based on the same explanation of the kosmotropic or chaotropic nature of ions.^91−95^ Consequently, with few exceptions like lysozyme^92^ and γD-crystallins^193^ below their isoelectric point wherein a reversal of the order of ions was observed, the present-day Hofmeister series for protein stabilization and denaturation is summarized in Figure 3.^91−95^

Despite the veracity in the ion trend of the Hofmeister series, it should be remarked that recent spectroscopic and molecular dynamics simulation observations have demonstrated specific ion effects on the protein hydration shell, change in dielectric constant at the protein–water interface for adsorption of polarizable ions at the hydrophobic sites, ion–ion interactions at the protein surface, etc.^96−101,194,195^ The validity of these concepts is not in question in the current work, and as such, “kosmotropic” ions will be defined as those of high charge density and with strong salting-out ability, while “chaotropic” ions are those of low charge density displaying a salting-in nature.^102^

Due to their nanometer size and scattering nature, proteins in an aqueous solution are considered colloidal particles.^196^ Hence, apart from the Hofmeister effect, other theories (Poisson–Boltzmann;^197^ Debye–Huckel;^198,199^ and Derjaguin and Landau, Verwey and Overbeek (DLVO)^200,201^) have been used to explain the stability of proteins as colloidal particles in salt solutions. According to the Poisson–Boltzmann and Debye–Hückel theories, the colloidal stability of proteins is maintained by screening their surface charge due to the formation of electrical double layers of salt ions in the salt solutions causing electrostatic repulsion.^197^ Hence, electrostatic interactions dominate the van der Waals interactions. The DLVO theory, on the other hand, considers both electrostatic and van der Waals interactions between the charged protein particles for their colloidal stability in salt solutions.^200,201^

Currently, it is highly difficult to explain the colloidal stability of a given protein based on a simple hard sphere model since specific counterion-protein, and co-ion-protein interactions at the protein surface dominate their colloidal stability. Several small-angle X-ray (SAXS) and neutron scattering (SANS) studies with proteins (ovalbumin, α-crystallins, bovine serum albumin, and Apoferrtin) above their isoelectric point have shown that the effectiveness of counterions in screening electrostatic repulsive interactions between proteins depends on the co-ion and molecular weight of the protein when NaCl, YCl_3_, and CH_3_COONa are used as salts.^202−204^ Similarly, contrasting interactions of salts with proteins are reported at much below or slightly below their isoelectric points.^205,206^ A decrease in solubility due to enhanced protein–protein interactions of lysozyme was reported between pH 3.0 and 9.0, whereas an increase in solubility was reported at pH > 9.0 up to 1.2 M NaCl.^205^ Furthermore, at pH 9.4, the lysozyme showed inverse Hofmeister effect for the anions in liquid–liquid phase transition at low salt concentration, whereas the opposite effect was observed at high salt concentrations.^84^ Another important aspect to be considered here is the colloidal stability of proteins in buffer solution since they have been extensively used to stabilize proteins at a specific pH.^207^ Although not much is investigated about the mechanism of protein stabilization in buffers, the most accepted mechanism is the binding of buffer ions as ligands at the oppositely charged amino acids on the protein surface.^207,208^ In the case when the salt is added to the buffer solution of proteins, the salt ions compete with the buffer ions to adsorb at the specific sites.^208^ Accordingly, the mechanism of salt ion-protein interactions is very specific and strictly depends on a variety of physicochemical conditions, like solution pH, temperature, properties of ions, salt concentrations, protein structure, molecular weight, and surface charge density.

### 2.4 Proteins in Osmolytes Aqueous Solutions

Osmolytes are mainly organic solutes generally considered to protect the native conformation of proteins from external stress stimuli, such as variations in temperature, salinity, and pH.^209−211^ However, they can induce both stabilizing and denaturing effects, depending on their preferential interactions with proteins. Among the major osmolytes present in cellular fluids of eukaryotes, polyols, and disaccharides (glycerol and sucrose), some free amino acids and their derivatives (taurine and P-alanine, octopine), methylamines (trimethylamine-N-oxide (TMAO), betaine), and sarcosine, have been classified as protein stabilizers. On the other hand, urea, arginine, and guanidinium have been classified as protein destabilizers.^209−211^

**Figure 4:**
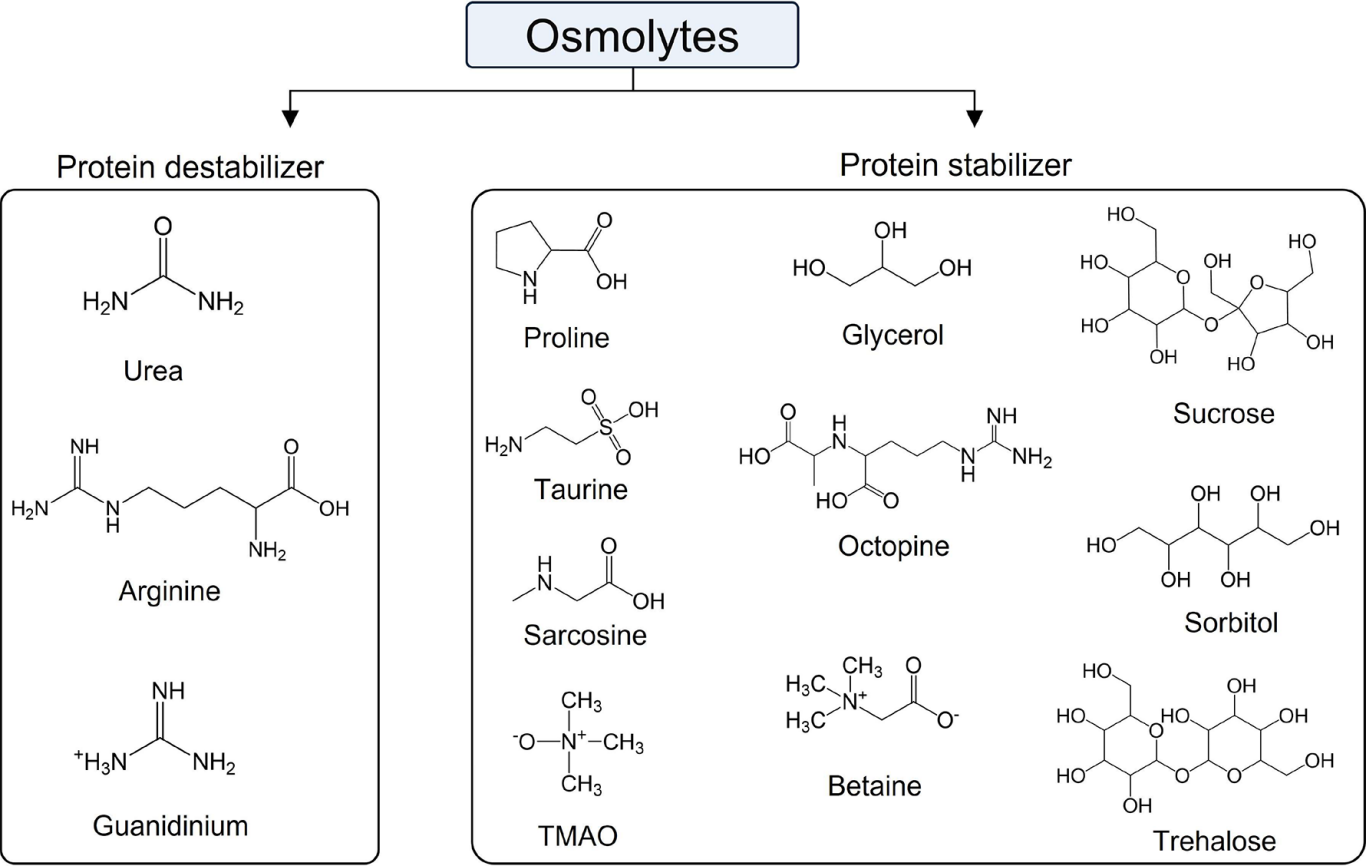
Molecular structures of well-known osmolytes based on their stabilizing and destabilizing effect toward proteins.

In 1982, Yancey et al.^209^ investigated the evolution of osmolytes in various organisms (from prokaryotes to eukaryotes) against water stress and their compatibility with proteins. One of the author’s major conclusions relays on the “genetic simplicity in proteins”. The genetic simplicity and presence of compatible solutes allow proteins to function in the presence of various solutes and at their high/low concentrations, without any modification in the structure/confirmation of a number of proteins and assisting in the proper functioning of cells.^209^ The mechanisms of interaction of osmolytes with proteins have been explained in line with the Hofmeister effects, even with counteracting systems, e.g., urea:methylamine in a 2:1 molar ratio, wherein methylamine overwhelms the destabilizing action of urea.^210^ A summary of osmolyte behavior toward proteins is provided in Figure 4.

Various other interaction mechanisms between osmolytes and proteins have been proposed over the years. However, if we consider it from a solution thermodynamic perspective, stabilizing osmolytes decreases the free energy of the protein’s unfolded state, favoring the folded population, whereas denaturing osmolytes lowers the free energy of the unfolded state, favoring the unfolded population of proteins. Takano et al.^212^ performed a molecular dynamics (MD) simulation on the effect of sarcosine on native RNase Sa, denatured RNase Sa, and four related proteins. The results gathered are in agreement with the osmophobic theory originally proposed by Bolen et al.,^213^ where “the osmolyte effect on protein stability is due to a solvophobic thermodynamic force which arises due to an unfavorable interaction of osmolytes mainly with the protein backbone.” These interactions can be determined from the transfer Gibbs energies of residue-specific contributions of proteins at the amino-acid level, from water to an osmolyte, based on solubility measurements.^212,213^ The stabilizing osmolytes get excluded from the vicinity of proteins in solution on account of unfavorable interactions, thus stabilizing it due to a “preferential hydration or preferential exclusion of solute” concept. This effect can be appraised by the preferential interaction parameter from equilibrium dialysis, which is positive for destabilizing osmolytes and negative for stabilizing osmolytes.^214^ This preferential exclusion concept is however supported by solvophobicity/solvophilicity, excluded volume, and surface tension (γ) effects. Solutes that increase the surface tension of the solution stabilize proteins, and *vice versa*, due to the preferential exclusion of solutes from the protein’s surface. Nevertheless, an opposing effect is observed with urea and TMAO, which respectively increase and decrease the surface tension of the solution and denature and stabilize proteins.^210^ Again, proteins are extremely complex molecules, and still there are many challenges to overcome aiming at finding a general theory capable of describing the reasons behind the protein’s stability/nonstability in solvents.

Street et al.^215^ stated that the thermodynamics concept is only descriptive and that there is no universal theory to explain the mechanisms by which osmolytes interact with proteins and accordingly affect their stability. The authors showed that there is no significant difference in the binding constant of stabilizing and denaturing osmolytes, thus attenuating the concept of preferential hydration or exclusion given above. A new model of osmolyte–protein–water interactions was proposed based on the transfer free energies of the protein’s backbone and osmolyte solution. This model was proposed based on the determination of transfer free energies (Δ*g*_tr_) of backbone models from water to 1 M osmolyte solutions. Although side chains do play a role, it is primarily the backbone Δ*g*_tr_ that determines the extent to which osmolytes either stabilize (i.e., Δ*g*_tr_ > 0) or destabilize (i.e., Δ*g*_tr_ < 0) the protein. The validity of this concept was proved by experimentally determined and calculated Δ*g*_tr_ values.^215^ This model was further validated by Auton et al.,^216^ who proposed the additive contribution of both backbone and side chains to calculate Δ*g*_tr_ for 46 proteins and 9 different osmolytes, stating that the contribution of the side chain always opposes the backbone contribution.

In the latest development using Kirkwood–Buff integrals for protein–water interactions (G_12_) and protein-osmolyte interactions (G_23_), using sucrose, trehalose, sorbitol, and poly(ethylene glycol) as osmolytes and the antibody antistreptavidin immunoglobulin gamma-1 (AS-IgG1) as the protein, Barnett et al.^217^ concluded that the protective or denaturing action of osmolytes is concentration-dependent.^217^ It was also shown that the stabilization or destabilization tendency of osmolytes depends on the type of protein.^217^ Additionally, osmolytes such as betaine, citrulline, and proline have been reported to inhibit insulin fibrillation, operating via preferential exclusion of the osmolyte and polar interactions with the protein surface.^218^ The thermal stabilization of lysozyme by Glycine (GLY), *N*-methylglycine (NMG), N,N-dimethylglycine (DMG), N,N,N-trimethylglycine (TMG), and trimethyl-N-oxide (TMAO) has also been reported. Stabilization effects were explained due to favorable interactions between the amino protons of osmolytes with water, acting as proton donors in protein–water interactions.^219^ The stabilization propensity decreased with an increase in the alkyl chains in the amino groups,^219^ which attenuates the concept of the TMAO stabilization by preferential exclusion by steric reasons. In this regard, recent studies^217,219^ have presented reservations against the existing concepts of “preferential hydration or exclusion”, thus seeking new explanations based on novel mechanisms. Even though the molecular-level mechanisms behind the protein’s improved stability are not completely understood, the use of ILs as osmolytes, mainly due to their tunable features, may represent a promising option to impart better thermal and long-term stabilization of proteins, as will be discussed later.

### 2.5 Protein Formulations with Surfactants

Surfactants are a class of organic amphiphilic molecules that reduce the surface tension of molecular solvents or ILs upon adsorption at the air–liquid interface.^26,220,221^ Due to their amphiphilic nature, they can self-assemble into well-organized structures in the nanometer or micrometer scales, such as micelles, vesicles, and lamellar phases in solution.^222^ Depending upon having charge or not, and their position, surfactants can be classified into ionic, nonionic, zwitterionic, and gemini. Their relevance in protein formulation can be appraised from their applications in pharmaceuticals, laundry, cosmetics, paints, coatings, and biochemical reactions.^143,144,223−229^ If we go back into history, the sodium dodecyl sulfate (SDS)-induced denaturation of methemoglobin, evidenced by the protein color change, was the first report on surfactant-protein systems.^230^ It was followed by a series of studies on protein–surfactant chemistry, comprising features such as solubilization, dissociation, denaturing effects, and interactions occurring between proteins and surfactants, as summarized by Putnam^231^ and Steinhardt et al.^232^ Otzen^224^ reviewed the developments in surfactant-protein chemistry, delineating the existing mechanisms of interactions inducing the protein unfolding/refolding/misfolding.

From the protein stability perspective, ionic surfactants (cationic, anionic, and zwitterionic) have been reported to be 1,000-fold stronger denaturants, when compared to common denaturants like urea and guanidinium chloride. Due to their differences in chemical structures, surfactants interact with proteins via different mechanisms. Given that surfactants in solution may exist as monomers, aggregates, shared aggregates, and vesicles/micelles, their interaction mechanisms with proteins follow multiple equilibrium steps, which have been classified into four regimes: monomeric (0 → C_1_), aggregation (C_1_ → C_2_), shared aggregation (C_2_ → C_3_), and post aggregation (>C_3_).^224,225^ C_1_ is the critical aggregation concentration (CAC), which is a signature of the completion of the monomers (surfactants as individual molecules/ion) binding on proteins by electrostatic or hydrophobic interactions. It should be highlighted that CAC in the absence of protein, or any other polyelectrolyte, is equivalent to the CMC or CVC. Between C_1_ → C_2_, clusters of surfactants begin to form on proteins via cooperative binding due to hydrophobic interactions, thus causing the protein to unfold. These complexes are generally stabilized by interactions between different protein molecules forming shared micellar complexes between C_2_ → C_3_. The rate constant of unfolding in this regime has a linear dependence on the surfactant concentration. However, just below C_3_, proteins form individual complexes with surfactants, which can be quantified as the number of surfactants *per* protein. After CMC or CVC, surfactant aggregates individually form micelles/vesicles in solution, which is also composed of denatured proteins.^233−236^ Otzen^224^ reviewed the technical difficulties in characterizing surfactant-protein complexes and highlighted the problems above CMC/CVC, which could possibly be overcome using small-angle neutron scattering (SANS).^237,238^

Case studies with bovine serum albumin (BSA) and human serum albumin (HSA) allowed establishing the order of induced denaturation by ionic surfactants at low concentration, according to the rank gemini > cationic > anionic > zwitterionic.^231,239,240^ On the other hand, nonionic surfactants, with few exceptions, have been reported as stabilizers of proteins in all concentrations.^231^ Although these are overall trends, there are exceptions depending on the protein and surfactant, as observed before with organic solvents and osmolytes. For instance, the anionic surfactant SDS, the cationic surfactant tetradecyltrimethylammonium bromide (TTAB), and the uncharged surfactants octyl maltoside and octyl glucoside have been reported to activate the enzymes *Thermomyces lanuginosus* and *cutinase*, particularly at low concentrations and below their CMC.^241,242^ Still, it should be kept in mind that it is not feasible to compare denaturant/renaturant effects of surfactants over enzymes and nonenzymatic globular proteins, mainly because the structural alterations occurring in proteins are different from functional alterations.^224^ Moreover, the observed opposite effects of SDS and cetyltrimethylammonium chloride (CTAC) on the stability of BSA and HSA further vindicate the specific nature of surfactant-protein interactions.^240^

**Figure 5:**
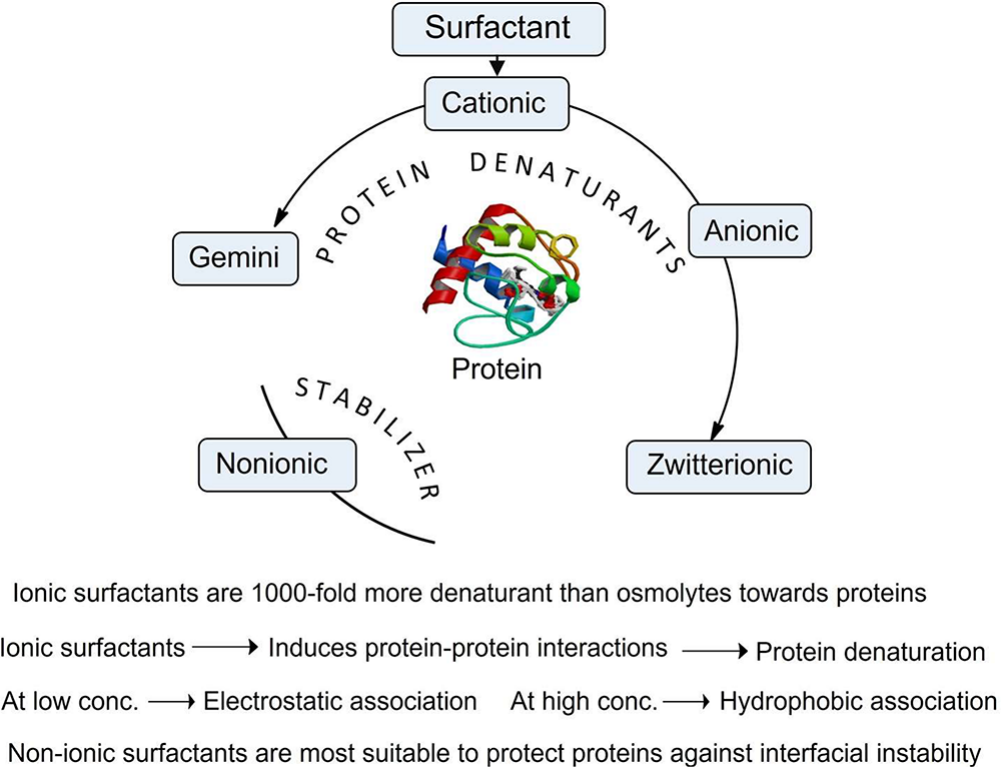
Schematic overview of the denaturation and stabilizing action of various types of surfactants toward proteins.

Apart from the surfactant charge, surfactant-protein interactions also depend on the solution pH, temperature, and stereochemistry.^243,244^ The stereochemical dependence of interactions opens the door to the design of surfactants for the selective extraction and stabilization of proteins. From the pharmaceutical point of view, many protein-based drugs need to be stored and shipped as solution formulations. The transportation causes severe agitation of the protein solutions, resulting in their interaction with container surfaces which can damage proteins due to the interfacial stress. Surfactants have been found to be the most suitable additives to avoid interfacial instability since they compete with proteins for the interfaces, upon migrating to the interfaces and protecting proteins during shaking or stirring.^245^ However, not all surfactants can stabilize proteins against interfacial damage since factors such as surfactant charge can act as a denaturant via ionic interactions with the protein surface. The generalized action of various types of surfactants toward proteins is shown in Figure 5.

Irreversible aggregation of proteins is caused by disulfide shuffling or stable hydrophobic association. In the case of therapeutic proteins (TPs), this effect has a direct impact on drug efficacy and immunogenicity.^246^ The most common TPs stabilized by surfactants are monoclonal antibodies, Interleukin 2, cytokines, Human chorionic gonadotropin HGH, and fusion proteins.^247−253^ Most of the surfactants used to stabilize TPs are nonionic, while possessing long alkyl side chains, such as polysorbate 80 (PS 80), PS 60, PS 20, and Poloxamer 188, in the concentration range between 0.005 and 0.16%.^247−253^ In the bulk phase, surfactant-protein complexes prevent protein–protein interactions, which is one the major causes of their denaturation.

In 2011, Lee et al.^254^ reviewed the molecular origin of the surfactant-based protein stabilization potential and concluded that surfactants stabilize proteins by their preferential location at the interface and/or their association with proteins in solution. On the other hand, Khan et al.^255^ reviewed the key interactions occurring in formulations of surfactants and therapeutic proteins, raising reservations against the proposed mechanisms and warranting further work to develop a clearer picture of the phenomenon.^255^ More recently, ionic liquid surfactants (SAILs) with higher surface activity compared to their conventional counterparts have been proposed,^111−140^ which are discussed below as an independent section.

### 2.6 Protein-Polymer Conjugates

**Figure 6:**
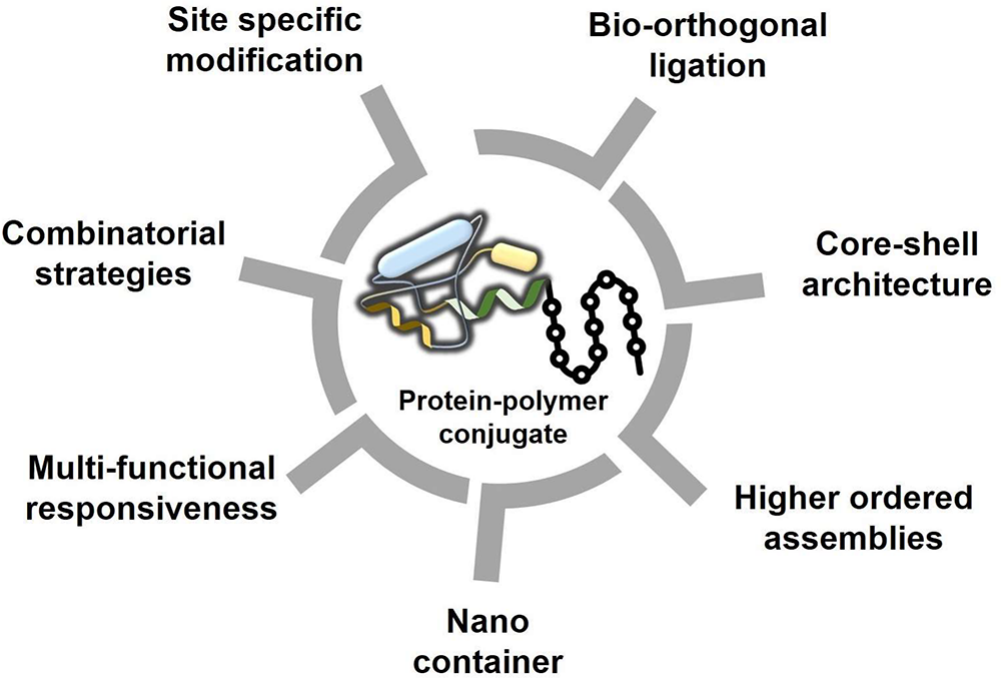
Overview of the design, synthesis, bioarchitectures, and bioactivities of protein–polymer conjugates for biobased applications.

Frequently administered TPs are afforded in high concentrations due to their longer half-lives in the bloodstream. Therefore, they are required to be stable for long periods at high concentrations, up to 10 mg/mL or 10,000 ppm, against aggregation-induced denaturation.^256^ One of the most prevalent strategies to curb this challenge is their conjugation with polymers, such as by the PEGylation approach.^256,257^ The first report in this direction was published by Abuchowski et al.,^258^ who PEGylated the protein BSA, leading to lower immunogenic response and higher circulation time in blood.^259^ This pioneering work led to the rise in frequency of works in protein-PEGylation, with some strategies already approved by the FDA.^260^ Currently, there are 23 PEGylated protein therapeutics clinically used to treat a wide range of diseases.^261^ The high promise of this strategy is due to the special characteristics of PEG, such as its nonfouling nature and resistance to opsonin binding, which is the major protein initiating the phagocytosis of foreign molecules in the bloodstream. Since opsonin exhibits a higher affinity for hydrophobic and charged species, being a hydrophilic and neutral polymer, PEG overturns the opsonin’s phagocytosis effect and thus increases the biocirculation time of conjugated proteins. Biocirculation time is also increased due to an increase in the molecular weight of PEG attached to the TPs, therefore reducing kidney clearance.^262−264^ In 2015, Pelegri-O’Day et al.^265^ reviewed the developments in TP-polymer conjugates in the purview of PEGylation and beyond. The authors stated that besides the described specific advantages, the strategy also has shortcomings, such as hypersensitivity, nonbiodegradability causing accumulation in tissues, accelerated blood clearance by anti-PEG antibodies, and recognition of PEG by the immune system and immunological responses.^266−269^ Alternative polymers evolved to overturn these limitations, namely N-(2-hydroxypropyl)methacrylamide (HPMA), poly(2-ethyl-2-oxazoline), poly(glutamic acid), poly(ethylene glycol) methyl ether methacrylate (pPEGMA), poly(carboxybetaine), polyglycerols, hydroxyethyl starch, polysialic acid, poly(*N*-hydroxypropyl)methacrylamide (pHPMA), polyglycans, and glycopolymers with pendant trehalose.^265^ Zhao et al.^270^ reviewed the TP-polymer conjugates in terms of methods of their synthesis. However, further improvements to increase the activity of the proteins by specific orthogonal biological function are needed to design polymers with precise sequence control and monodispersity.^265,270^ The recent rise of polymeric ILs as a conjugated polymer can further improve the protein/TP solubility/stability,^153,271^ which is described later in this review. A pictorial summary of different designs, syntheses, and architectures of protein–polymer conjugates proposed for bioapplications is shown in Figure 6.

## 3 Ionic Liquid-Protein Systems

The structural diversity of ILs, responsible for their tunable nature and properties, endowed them with the “luxury” to interact with proteins by different mechanisms. Some ILs have shown great promise as protein stabilizers and thus as potential protein packaging materials/solvents, which is a relevant application in the field of therapeutic proteins. Therefore, this section will cover the works published to date, divided according to the IL use, i.e., as native solvents, co-solvents, adjuvants, surfactants, IL-based aqueous biphasic systems (IL-ABS), and Poly(IL)-protein conjugates. The detailed mechanisms of interaction between ILs and proteins are discussed in parallel with those occurring in well-known protein stabilizers to come up with a rational perspective of the IL’s potential. Apart from protein packaging and relevance with TPs, other applications of IL-protein systems are addressed here.

### 3.1 Ionic Liquids as Native Solvents

**Figure 7:**
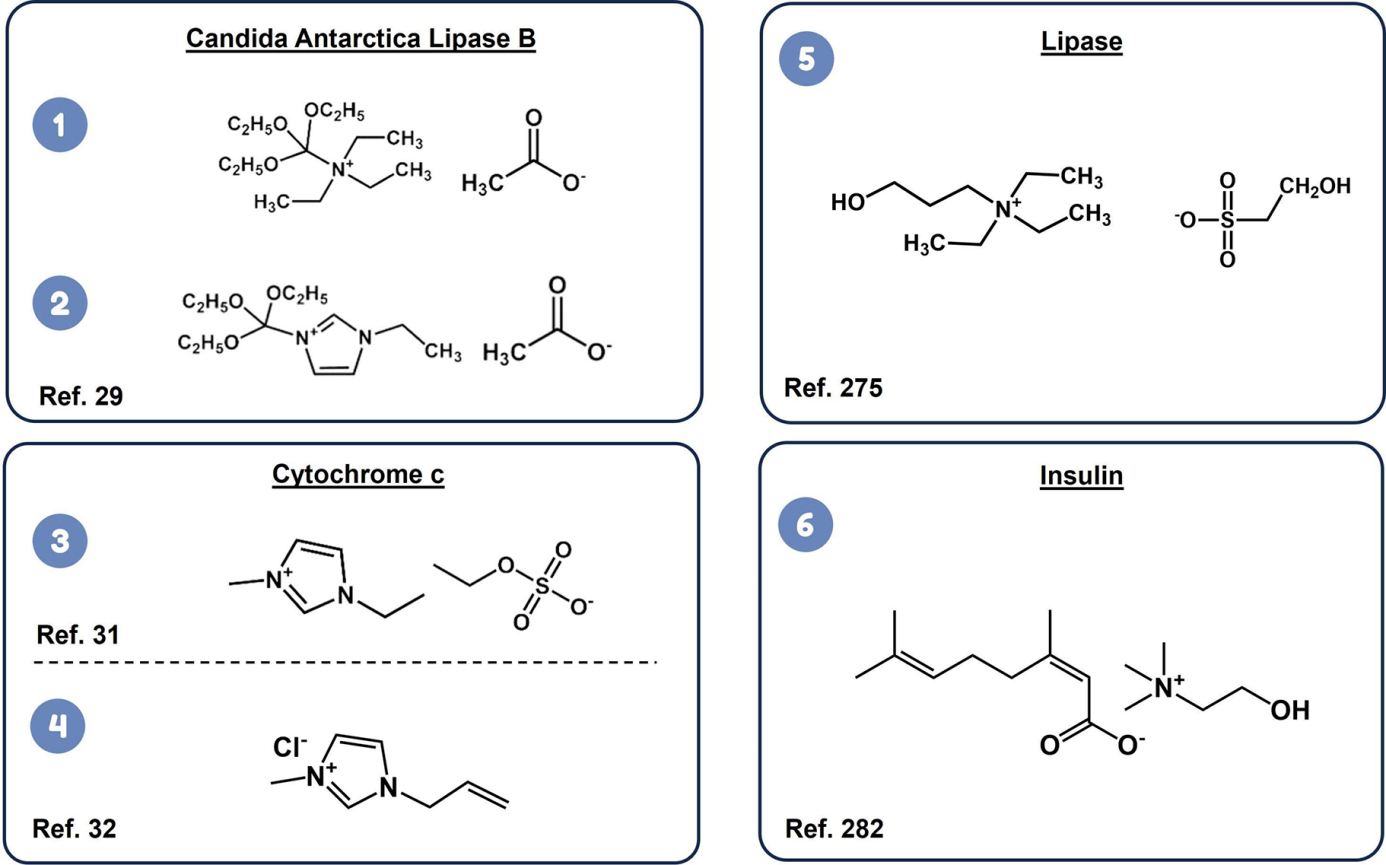
Molecular structures of the most promising neat ionic liquids for both solubilization and stabilization of specific proteins.^29,31,32,275,282^

Due to their ionic and hygroscopic nature, it is impossible to dry all ILs up to 100% as a ppm level of water always remains with ILs. Therefore, most of the marketed ILs are always tagged with a given percentage of water. However, despite the presence of water at ppm levels, generally considered enough to form hydration layers around proteins, only a scarce number of proteins are soluble in dry ILs or NILs,^28−34^ contrary to the larger number of organic solvents that act as good protein solvents. As discussed earlier, ILs comprise nanoscale heterogeneous domains, i.e., polar/nonpolar nanodomains,^19−27^ and as such must solvate proteins operating via different mechanisms. A summary of the proteins that have been solubilized in native ILs, along with their secondary structure and employed ILs, is shown in Table 1. The molecular structure of the most promising NILs for the stable solubilization of specific proteins is shown in Figure 7.

**Table 1:** Summary of Proteins Dissolved in Native ILs along with Their Secondary Structure

Protein	Secondary Structure	Ionic Liquid
CAL B^28^	**α/β**	[C_2_mim][C_2_OSO_3_], [C_4_mim][Lac], [C_2_NH_3_][NO_3_], [C_4_mim][NO_3_], [(C_2_)_3_C_1_N][C_1_OSO_3_]
CAL B^272^	**α/β**	[(C_2_)_3_C_1_N][C_1_OSO_3_] and [C_4_mim][C_2_N_3_]
CAL B^274^	**α/β**	[HOPmim][NO_3_]
CAL B^29^	**α/β**	[Me(OEt)_2_-Et-Im][OAc], [Me(OEt)_3_-Et-Im][OAc], [Me(Opr)_3_-Et-Im][OAc], [Me(Oet)_3_-Et_3_N][OAc], [C_4_mim][dca], [C_2_mim][OAc] [C_8_mim[OAc], [C_4_mim]HCOO], [Me(Oet)_3_-Et-Im][C_2_N_3_], [Me(Oet)_3_-Et_3_N][HCOO], [Me(Oet)_2_-Et_3_N][Oac], [Me(Oet)_3_-Bu-Im][Oac], [C_2_mim][C_2_OSO_3_], [(C_4_)_4_N][HCOO], [Amm110]Cl, [Me(Oet)_7_-Et-Im][OAc], [Me(Oet)3-MeOEtOMe-Im][OAc], [Me(Oet)3-Me-Et-Im][OAc]
CAL B^275^	α/β	[C_2_OHmim][HOC_1_SO_3_], [C_2_OHmim][HOC_2_SO_3_], [C_3_OHTEA][HOC_2_SO_3_]
Cellulase^30^	**α/β barrel**	[C_1_mim][Cl],[(OHC_2_H_5_)_3_C_1_N][C_1_OSO_3_]
Cytochrome c^276^	All-α	[C_2_OHmim][Tf_2_N], [C_3_OHmim][Tf_2_N], [C_2_OC_1_mim][Tf_2_N], [C_6_OHmim][Tf_2_N], [C_8_OHmim][Tf_2_N]
Cytochrome c^31^	**All-α**	[C_2_mim][C_2_OSO_3_]
Cytochrome c^32^	**All-α**	[C_4_mim]Cl, [Amim]Cl
Silk Fibroin^277^	**Cross-β**	[C_4_mim]Cl, [C_4_mim]Br, [C_4_mim]I, [C_4_(C_1_)_2_im]Cl, [C_2_mim]Cl
Keratin^33^	**α+β**	[DMEA][HCOO]
Keratin^278^	**α+β**	[C_4_mim][OAc], [Cho][TGA], [Cho][Pn], [C_4_mim]Cl, [TMG][Pn]
Zein^279^	**All-α**	[C_4_mim] [C_2_N_3_], [C_4_mim]Cl
Zein^34^	**All-α**	[C_4_mim][OAc], [C_2_mim][OAc], [C_2_mim][dca], [C_4_mim]Cl, [C_1_mim][OAc], [C_1_mim][HSO_4_], [C_1_mim][HCOO]
HSA^280^	**All-α**	[C_4_mim][OAc], [C_4_mim][SCN]
Ovalbumin^280^	**α+β**	[C_6,6,6,14_P]Cl [C_2,4,4,4_P][(C_2_)_2_OPO_3_], [C_4_mim][OAc], [C_4_mim][SCN], [C_2_mim][C_2_OSO_3_]
Myoglobin^280^	All-α	[C_2,4,4,4_P][(C_2_)_2_OPO_3_], [C_4_mim][OAc], [C_4_mim][SCN]
α-Chymotrypsin^280^	All-β	[C_2,4,4,4_P][(C_2_)_2_OPO_3_], [C_4_mim][OAc], [C_4_mim][SCN], [C_2_mim][C_2_OSO_3_]
Lysozyme^280^	α+β	[C_1,4_Pyr][C_2_N_3_], [C_4_mim][OAc], [C_4_mim][SCN], [C_2_mim][C_2_OSO_3_]
Lactoferrin^280^	α+β	[C_4_mim][OAc], [C_4_mim][SCN], [C_2_mim][C_2_OSO_3_]
Gelatin^281^	**Random coil**	[C_*n*_NH_3_][NO_3_] (*n* = 2, 3, 4), [CnNH_3_][NO_3_] + [C_4_mim]Cl
Insulin^282^	α+β	[Cho][gerenate]

Different studies on NIL-protein systems have emphasized the role of covalent and noncovalent interactions both in protein solubility and stabilization. Lau et al.,^28^ in 2004, were the first to show the dissolution of the enzyme *Candida antarctica* lipase B in NILs, *viz*. [C_2_mim][C_2_OSO_3_], [C_4_mim][Lac], [C_2_NH_3_][NO_3_], [C_4_mim][NO_3_], and [(C_2_)_3_C_1_N][C_1_OSO_3_] at 40 °C. However, only [(C_2_)_3_C_1_N][C_1_OSO_3_] stabilized the enzyme, indicated by the retention of the secondary structure and trans-esterification activity, which is due to the H-bonding between the IL ions and the protein surface. The authors concluded the need for a balance between steric factors and hydrogen bond accepting/donating properties of ILs for protein solubilization and stabilization.^28^ In 2006, Sheldon and co-workers^272^ studied the comparative stability and activity of *Candida antarctica* lipase B and its cross-linked enzyme aggregate (CLEA) in [(C_2_)_3_C_1_N][C_1_OSO_3_] and [C_4_mim][C_2_N_3_].^272^ They found high stability of the enzyme in [(C_2_)_3_C_1_N][C_1_OSO_3_] compared to that in [C_4_mim][C_2_N_3_] due to the strong hydrogen bonding nature of the first IL. However, higher activity of CLEA was observed in [C_4_mim][C_2_N_3_], implying the role of protein engineering to increase enzyme activity in ILs.^272^

The relevance of H-bonding for protein stability in NILs was also demonstrated from simulation studies on the solvation of small cyclic hexapeptide in ILs, namely, [C_4_mim]Cl and [C_4_mim][PF_6_].^273^ The H-bonding between the hydroxyl groups of the peptide and the IL anion was shown to stabilize the peptide in [C_4_mim]Cl compared to that in [C_4_mim][PF_6_].^273^ Bermejo et al.^274^ also reported higher solubility of *Candida antarctica* lipase (12%) in [HOPmim][NO_3_] containing a cation with an H-bonding site. Zhao et al.^29^ investigated the dissolution and stabilization of *Candida antarctica* lipase B in 18, nonfunctionalized and ether-functionalized ammonium and imidazolium cation containing ILs, with [OAc], [HCOO], [dca], and [C_2_OSO_3_] anions. The order of solubility found was as follows: [Me(OEt)_2_-Et-Im][OAc] ≈ [Me(OEt)_3_-Et-Im][OAc] ≈ [Me(OPr)_3_-Et-Im][OAc] ≈ [Me(OEt)_3_-Et_3_N][OAc] > [C_4_mim][C_2_N_3_] ≈ [C_2_mim][OAc] ≈ [C_8_mim[OAc] ≈ [C_4_mim]HCOO] ≈ [Me(OEt)_3_-Et-Im][dca] > [Me(OEt)_3_-Et_3_N][HCOO] ≈ [Me(OEt)_2_-Et_3_N][OAc] ≈ [Me(OEt)_3_-Bu-Im][OAc] > [C_2_mim][C_2_OSO_3_] ≈ [(C_4_)_4_N][HCOO] > [Amm110]Cl ≈ [Me(OEt)_7_-Et-Im][OAc] ≈[Me(OEt)_3_-MeOEtOMe-Im][OAc] ≈ [Me(OEt)_3_-Me-Et-Im][OAc]. The ether-functionalized ILs, [Me(OEt)_3_-Et_3_N][OAc] and [Me(OEt)_3_-Et-Im][OAc], were found to be the most effective in preserving the secondary structure and activity of the enzyme.^29^ Ou et al.^275^ showed that the ionizing-dissociating abilities of ILs having hydroxyl functionalities paralleled the catalytic activity trend of lipases dissolved in these ILs. The studied ILs—[C_2_OHmim][HOC_1_SO_3_], [C_2_OHmim][HOC_2_SO_3_] and [C_3_OHTEA][HOC_2_SO_3_]—provide a nondenaturing and noninhibitory environment to the enzyme due to their ionizing-dissociating abilities. This evidence is critical for the development of ILs for the stabilization of not only TPs but as well to other relevant proteins with a moderate hydrophobic nature.

Besides *Lipases*, few other enzymes, like Cellulase and Cytochrome c, have been investigated in NILs. Bose et al.^30^ investigated the reactivity and stability of commercial Cellulases from *Trichoderma reesei* in eight ILs, reporting their structural and functional stability in [C_1_mim]Cl and [(OHC_2_H_5_)_3_C_1_N][C_1_OSO_3_]. However, HEMA imparted much higher thermal stability to *Cellulase*, demonstrated by its high melting temperature (*T*_m_ = 115–125 °C) compared to the *T*_m_ = 70–94 °C in [C_1_mim]Cl. Using an indirect strategy by complexation with dicyclohexano-18-crown-6, Shimojo et al.^276^ solubilized Cytochrome c in hydroxyl and oxy functional imidazolium-based ILs, paired with the bistriflamide anion. The enzyme solubility according to the IL cation follows the order [C_2_OHmim] > [C_3_OHmim] > [C_2_OC_1_mim] > [C_6_OHmim] > [C_8_OHmim]. The dissolution step, however, changed Cytochrome c from an electron transfer to a peroxidase enzyme due to the replacement of axial Met80 from the sixth coordination position by amino acid from the peptide chain.^276^

Bihari et al.^31^ reported the dissolution of Cytochrome c in [C_2_mim][C_2_OSO_3_], achieved by the complexation of the free heme coordination site imidazolium cation. The [C_2_mim][C_2_OSO_3_] preserved the secondary structure of the enzyme with an enhancement in its peroxidase activity.^31^ Tamura et al.^32^ explained the solubility of Cytochrome c in [C_4_mim]Cl and [Amim]Cl at 80 °C based on the Kamlet–Taft parameters (β and π*) of ILs. They stated that ILs with β and π* higher than 0.70 and 1.17, respectively, can dissolve Cytochrome c. Indeed, if we look back into the earlier report of Cytochrome c solubility in [C_2_mim][C_2_OSO_3_],^31^ we find that [C_2_mim][C_2_OSO_3_] has a β value of 0.71, thus supporting the Tamura et al.^32^ views. Hence, the role of H-bonding in solubilization and stability of enzymes is one common denominator for all the ILs discussed.

Other than enzymes, many structural and functional proteins have been dissolved in native ILs via different dissolution mechanisms. Phillips et al.^277^ reported the dissolution of various percentages of protein silk fibroin in [C_4_mim]Cl (13.2%), [C_4_mim]Br (0.7%), [C_4_mim]I (0.2%), [C_4_(C1)_2_im]Cl (8.3%), and [C_2_mim]Cl (23.3%), in which the dissolution is achieved by the disruption of hydrogen bonds in the crystalline domains of the cross-β structure.^277^ Chen et al.^278^ reported the dissolution of natural protein fibers such as wool, human hair, and silk in ILs, viz. [C4mim][OAc], [Choline][TGA], [Choline][Pn], [C4mim]Cl, and [TMG][Pn], occurring due to cuticle swelling or surface interactions.^278^ Idris et al.^33^ showed the dissolution of keratin in [DMEA][HCOO], yet without reporting any dissolution mechanism.

Biswas et al.^279^ reported the dissolution of zein in [C_4_mim][C_2_N_3_] and [C_4_mim]Cl and used the solution for successful acylation of the native protein. Tomlinson et al.^34^ further explored the solubility of zein in imidazolium-based ILs comprising the anions [OAc], Cl, [dca], [HCOO], and [HSO_4_]. They found the highest solubility in [C_1_mim][OAc] and [C_2_mim][C_2_N_3_] and explained the improved solubility based on the molar volume of ILs.

Strassburg et al.^280^ looked beyond the largely studied imidazolium-based ILs and reported the solubilization of BSA, HSA, Ovalbumin, Myoglobin, α-Chymotrypsin, Lysozyme, Cytochrome c, and Lactoferrin in quaternary phosphonium-, quaternary ammonium-, and pyrrolidinium-based ILs, alongside imidazolium-based counterparts for comparison purposes. Among these, [C_6,6,6,14_P]Cl dissolved ovalbumin; [C_2,4,4,4_P][(C_2_)_2_OPO_3_] dissolved BSA, Ovalbumin, Myoglobin, α-Chymotrypsin, and Cytochrome c; [C_1,8,8,8_N]Cl dissolved BSA; and [C_1,4_Pyr][dca] dissolved Lysozyme. However, imidazolium-based ILs still proved to be the first-choice cation for proteins, as [C_4_mim][OAc] and [C_4_mim][SCN] dissolved all the studied proteins. Nevertheless, further temperature-dependent stability analysis using DLS and FTIR revealed only small alterations in lysozyme structure upon heating at 80 °C in [C_2,4,4,4_P][C_2_OPO_3_] compared to that in [C_2_mim][C_2_OSO_3_].^280^

Mehta et al.^281^ reported high solubility (58 to 87%) of protein gelatin in neat alkyl ammonium nitrates ([C_*n*_NH_3_][NO_3_], *n* = 2, 3, 4) and their binary mixtures with [C_4_mim]Cl. The H-bonding of ILs with gelatin was suggested as the major driving force for protein solubility, as appraised by solubility experiments with an increasing [C_4_mim]Cl concentration and decreasing gelatin solubility in the [C_*n*_NH_3_][NO_3_] + [C_4_mim]Cl mixture.^281^

**Figure 8:**
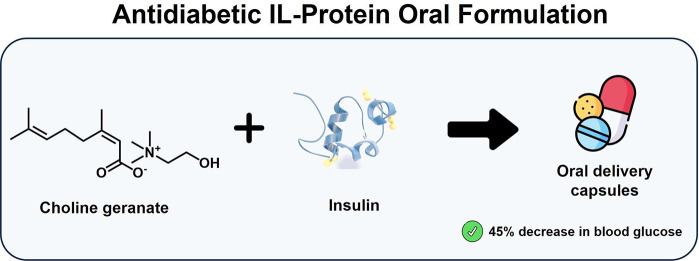
Illustration of the [Cho][gerenate]-insulin oral formulation.

In a recent significant development, Banerjee et al.^282^ (Figure 8) reported the stable dissolution of Insulin (therapeutic protein) in choline geranate, [Cho][gerenate], as an oral insulin formulation. The 10 U/kg insulin-[Cho][gerenate] was orally delivered in enterically coated capsules using an oral gavage, resulting in a sustained decrease in blood glucose of up to 45%, thus establishing the relevant role of neat cytocompatible IL-protein formulations for pharmaceutical applications.^282^

#### 3.1.1 Solubilization Mechanisms of Proteins in NILs

**Figure 9:**
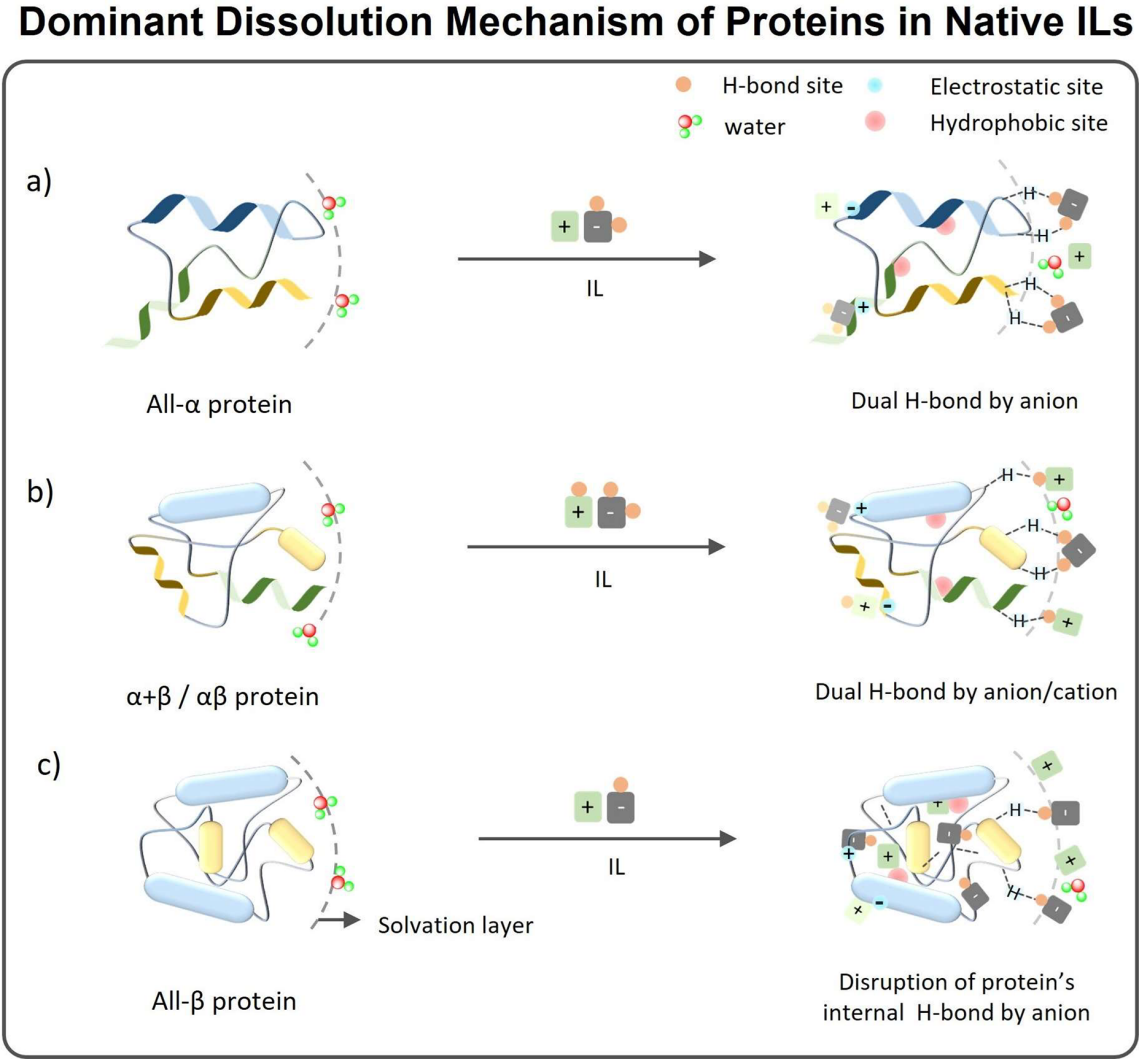
Dominant dissolution mechanism of proteins in a nano heterogeneous structure of native ILs. (a) Hydrogen bonding of protein with the anion of ILs, mainly observed for all-α proteins. (b) Hydrogen bonding of protein with both cation and anion of ILs, observed mainly in α/β or α+β proteins. (c) Disruption of the internal hydrogen bonds of proteins by the IL anion and steric interactions with the cation, mainly observed in all-β proteins. Besides the dominant H-bonding interactions, other interactions, like electrostatic and hydrophobic interactions, play a secondary role in improving the dissolution of proteins in ILs.

The above-discussed reports demonstrate that a relevant number of proteins, namely, CAL B, CRL, Cytochrome c, BSA, HSA, α-chymotrypsin, ovalbumin, myoglobin, lysozyme, lactoferrin, silk fibroin, Zein, keratin, and gelatin, can be solubilized in NILs. However, and with the exception of a few cases, solubilization in NILs resulted in alterations of the protein’s secondary and tertiary structure.^28^ Although all are connected, three different rationales have been used to describe the dominant molecular-level mechanisms responsible for the protein’s solubilization/stabilization: (1) H-bonding formation between the proteins and the ILs; (2) Kamlet–Taft parameters (α, β, and π*), which in turn show the hydrogen bond donor capacity (HBD) and hydrogen bond acceptor capacity (HBA) and dipolarity/polarizability of the IL;^283^ and (3) hydrogen bond disruption in proteins. Overall, from the works addressed, it can be seen that hydrogen-bonding of the IL ions with the protein surface has been the most accounted phenomenon observed with proteins bearing α/β or α+β secondary structural conformation. For proteins with an all-α conformation (Cytochrome c, BSA, HSA), higher β and π values of ILs have been accounted for as the reason for improved solubility, implying strong H-bonding (β value >0.7) between the IL ions and the protein. In the case of proteins with higher content of β-sheet structure, such as keratin and silk fibroin, disruption of intramolecular bonds of proteins by IL anions has been identified as the main reason for enhanced solubility. These views, proposed as ruling the solubilization of proteins in native ILs, are schematically presented in Figure 9. However, it is noted that H-bonding is dominant but not the sole force for the solubilization of proteins in NILs. Considering the broad polar heterogeneity of both proteins and ILs, protein dissolution/stabilization in native ILs involves secondary forces, such as Coulombic interactions, hydrophobic interactions of nonpolar groups, and van der Waal’s interactions. Experimental challenges for understanding of protein-neat ILs interaction arise due to the inapplicability of common techniques like in solution NMR and circular dichroism in near-pure ILs. Molecular dynamics (MD) simulations offer an alternative to better understand the interactions taking place.^284^ Shim et al.^273^ carried out MD studies on the solvation of cyclic hexa-peptides in the ILs [C_4_mim]Cl and [C_4_mim][PF_6_], revealing peptide structure distortion by electrostatic interactions occurring between the peptides and both ILs. The distortion was lower in the [C_4_mim]Cl due to the stabilization of the peptide by intermolecular H-bonding.^273^ Burney et al.^285^ simulated *Candida rugosa* lipase (CRL) in [C_4_mim][PF_6_] and [C_4_mim][NO_3_], concluding that ILs dampen protein dynamics, trapping the system near its initial structure due to electrostatic interactions.

Klähn et al.^286^ studied the solvation and stability of CAL-B in various imidazolium and guanidinium cations using MD simulations. The authors concluded that the interaction of CAL-B with the IL anion is dominated by Coulombic interactions, whereas that with the cation is by van der Waal’s interactions. The authors also showed that smaller ions led to stronger electrostatic screening with the solvent and hence stronger interaction with the enzyme. On the other hand, ions with large size and more dispersed surface charge increase enzyme-IL interactions, leading to the destabilization of the enzyme with decreased solvation. Overall, MD simulations provide molecular-scale insights into IL-protein interactions, overcoming experimental limitations in studying protein stabilization in native ILs.

As stated in the beginning of this section, it must be noted that even in a dry state few water molecules coexist with ILs, which have been shown at the desired location around proteins. This phenomenon ultimately leads to the reference of H-bonding acting in a different direction. ILs exhibiting hydroxyl or oxy functionalities in both ions^29,275,276^ are improved solubilizers and stabilizers of proteins. Therefore, these ILs could represent the most efficient ones for the solubilization of proteins and TPs in NILs, while keeping their stability.

**Figure 10:**
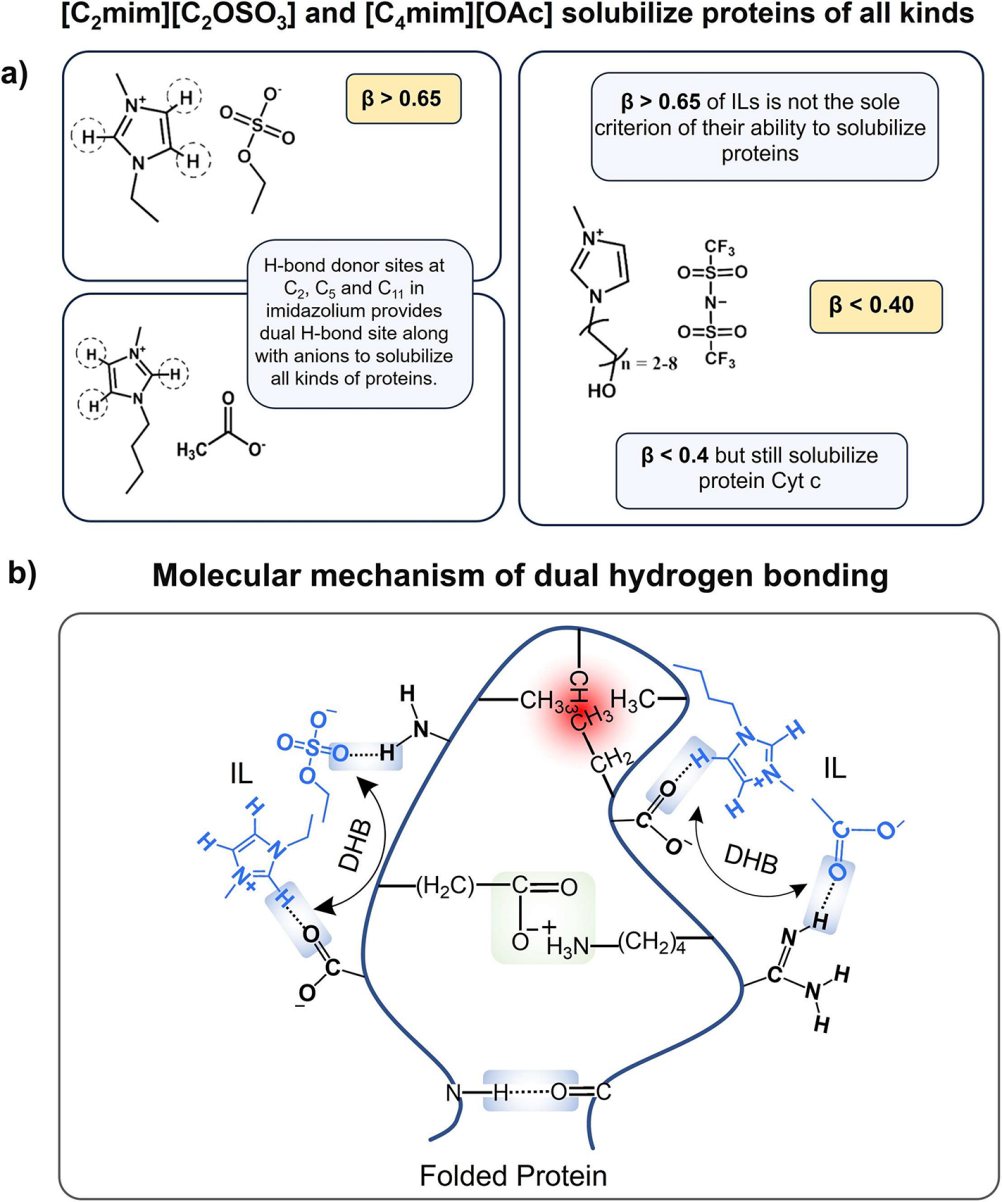
(a) Molecular structures of [C_2_mim][C_2_OSO_3_] and [C_4_mim][OAc], which solubilize all kinds of proteins, and [OHC_*n*=2–8_mim][NTf_2_] which dissolve Cytochrome c despite having β < 0.4. (b) depiction of the molecular mechanism of dual hydrogen bonding (DHB) by [C_2_mim][C_2_OSO_3_] and [C_4_mim][OAc] with folded proteins.

However, a special behavior has been observed with the ILs [C_2_mim][C_2_OSO_3_] and [C_4_mim][OAc], which have shown the ability to solubilize proteins of all kinds of secondary structures, namely, all-α (Cytochrome c, BSA, HSA, myoglobin), all-β (α-chymotrypsin, keratin), α/β (CAL B), and α+β (Ovalbumin, Lysozyme, and Lactoferrin). Because of their special behavior, we went into detail regarding their structures to uncover why these ILs are more suitable for protein solubilization, as discussed below. Comparing the β value (reflecting the hydrogen-bond basicity of the IL, and mainly dictated by the IL anion) they are on a suitable scale (>0.65)^32,148^ for dissolving biopolymers. Furthermore, [C_4_mim][OAc] has a higher hydrogen-bond basicity (β = 1.18)^287^ than [C_2_mim][C_2_OSO_3_] (β = 0.71),^288^ being thus a better solvent for proteins (Figure 10a).

In the same line, imidazolium-based ILs having β > 0.65, such as those containing Cl and [C_2_N_3_] anions, are good candidates to dissolve proteins as well, as experimentally demonstrated.^29,30,32,34,277,280^ In contrast, few ILs such as [OHC_*n*=2–8_mim][NTf_2_] with β < 0.4 can also solubilize proteins.^276^ Therefore, β may not be the sole criterion for the special dissolving behavior observed by [C_2_mim][C_2_OSO_3_] and [C_4_mim][OAc], with electrostatic, hydrophobic, and van der Waal’s interactions also playing a role.

When addressing the electronic structure of the [C_2_mim][C_2_OSO_3_] ion pairs as a dry solvent, it has been reported to exist in three conformations, varying based on the H-bonding ratio between the ethylsulfate anion and hydrogens of the imidazolium cation.^289^ The lowest-energy conformer is involved in 6 hydrogen bonds between the cation and the anion, wherein the C2–H9, C5–H11, and C4–H10 bonds show bifurcated interactions. However, two other conformations have free acidic hydrogens at the C_5_ position. This hydrogen can particularly participate in H-bonding with carboxylate groups at the protein surface along with H-bonding between the anion and the amino groups, thus providing dual H-bonding sites like observed in [C_2_OHmim][HOC_1_SO_3_] stabilizing BSA.^275^ As far as the case of [C_4_mim][OAc] is concerned, a similar phenomenon could be depicted. Even though we could not find its electronic structure, [C_2_mim][OAc] also contains free acidic hydrogens at the C_5_ position^289^ and therefore could be contributing toward dual H-bonding and improved protein solubilization of different conformations. Figure 10b shows a schematic overview of the dual hydrogen-bonding phenomenon at the molecular level.

Overall, hydrogen bonding occurring between ILs and proteins providing a water-like environment at the surface of the protein, ideally in the form of dual hydrogen bonding as discussed above, seems to be the major governing force of protein solubility and stability in NILs. Further augmentation in this force could be sought by analyzing the effects of adding small amounts of water into NILs, in which ILs could act as co-solvents, as discussed in the following section.

### 3.2 Ionic Liquids as Co-solvents

**Figure 11:**
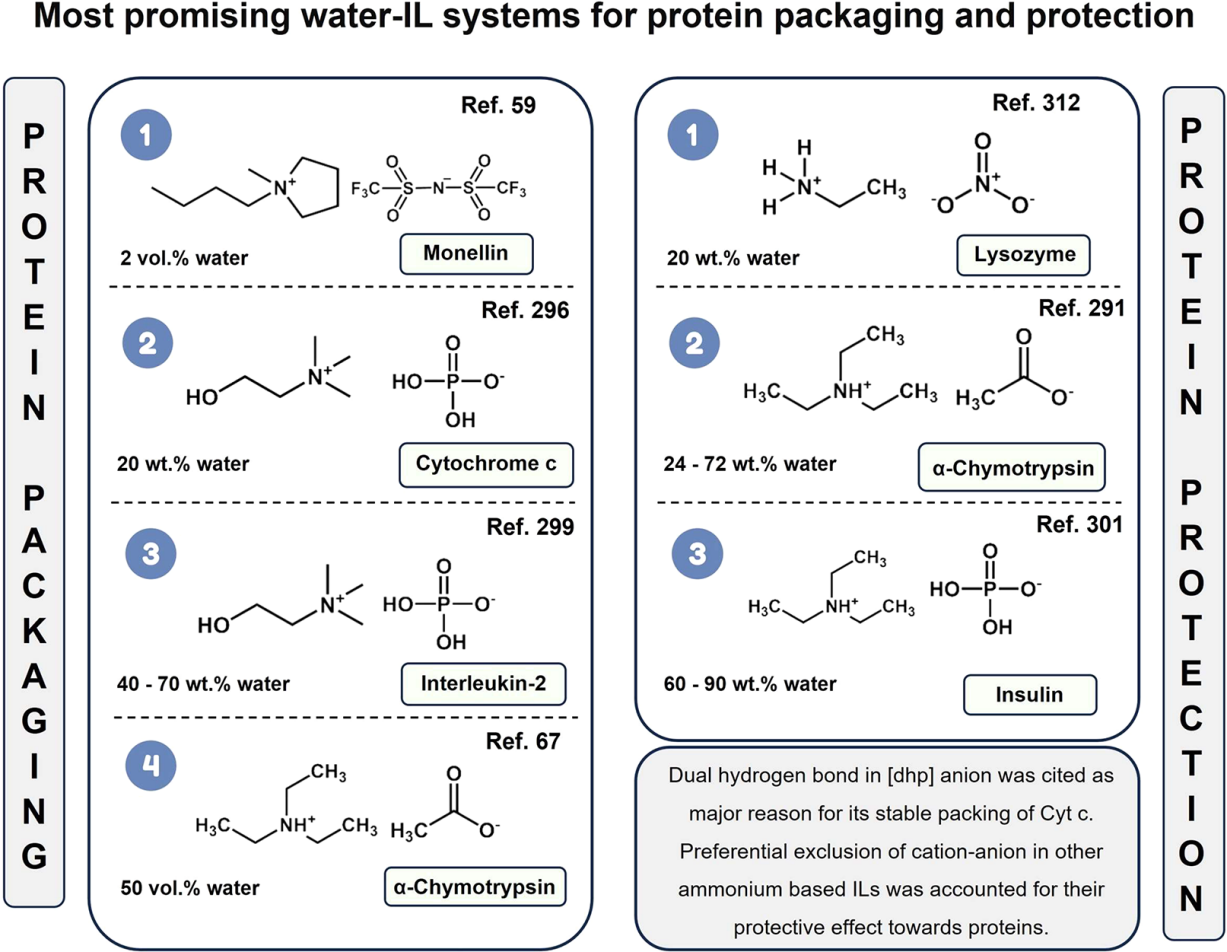
Most promising water-IL systems for protein packaging and protection.^59,296,299,67,312,291,301^

Following the proteins that showed robustness when solubilized in NILs, a significant amount of work has followed with the addition of 2–50% water, resulting in A-ILCS. These IL-water mixtures, in which the IL could act as co-solvent, lead to given advantages, including (1) providing sufficient water to hydrate the protein and (2) imparting thermal stability to the proteins by IL ions present in the form of clusters in the hydration layer.^24,27,54^ The chemical structures and composition of the most promising water-IL systems for protein packaging and protection are provided in Figure 11.

The mode of interaction, solvation, and stabilization of proteins by A-ILCS depends on the structure and polarity of the IL cation/anion and water content. For example, the earliest work in this direction was published by Baker et al.^59^ in 2004, who showed the stabilization of the protein monellin in A-ILCS with a hydrophobic IL, [C_4_mpy][Tf_2_N]:H_2_O (98:2 v/v) mixture, up to 105 °C compared to 40 °C in pure water. Using Trp fluorescence as an internal spectroscopic handle, the authors observed a blue shift in Trp fluorescence above 105 °C, which accounted for stripping off the water from the protein hydration layer by [C_4_mpy][Tf_2_N]. Moreover, calculated entropies of unfolding in [C_4_mpy][Tf_2_N]:water (136 J·K^–1^·mol^–1^) compared to water (250 J·K^–1^·mol^–1^) indicated more rigid solvation of the protein in the IL:water mixture.^59^ Diego et al.^60^ studied the structural stabilization of α-chymotrypsin in A-ILCS comprising [(C_2_)_2_mim][Tf_2_N]:1-propanol:H_2_O (85.5:12.5:2, v/v) using DSC, CD, and fluorescence techniques. From a thermodynamic point of view, the melting temperature of the protein increased by 10.4 °C, with an increase in the enthalpy of denaturation (dH_cal_) by 3-fold compared to water.

**Figure 12:**
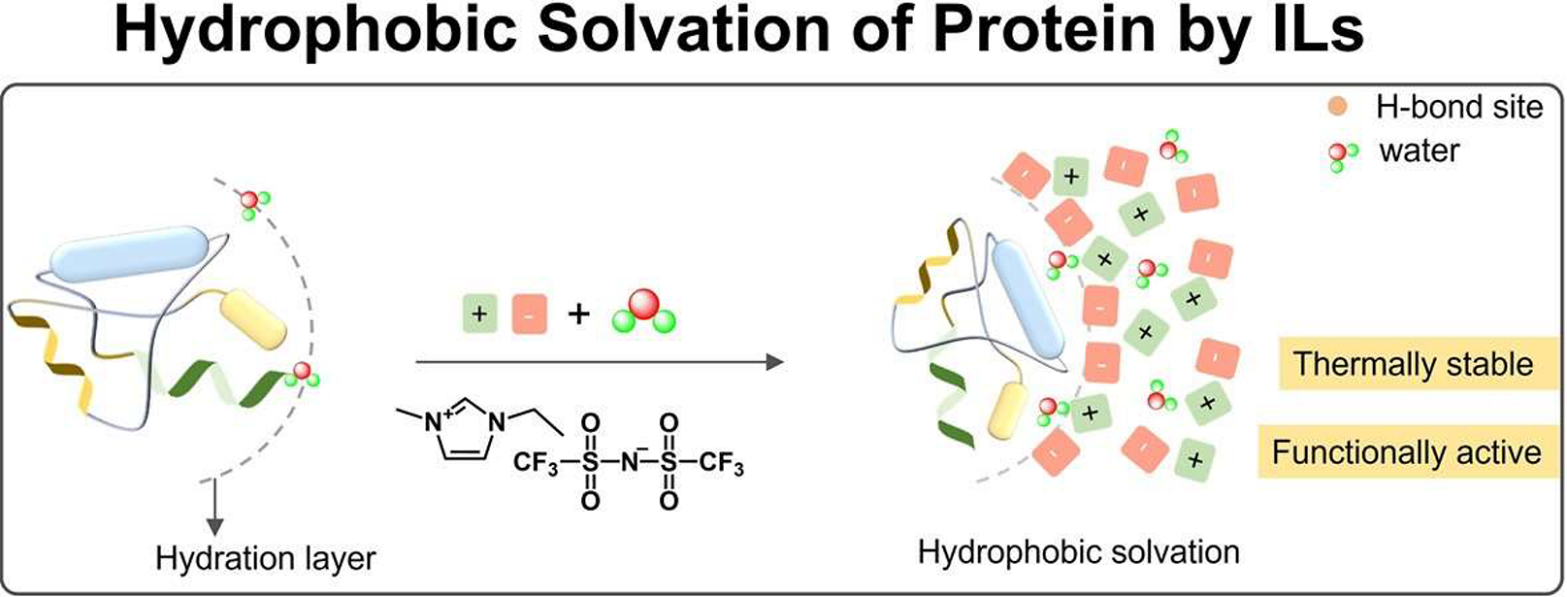
Illustration of the mechanism of protein stabilization by A-ILCS composed of hydrophobic ILs.

From a structural point of view, the [(C_2_)_2_mim][Tf_2_N] mixture stabilized the protein via the formation of a flexible and more compact 3D structure, while preserving the essential water shell. The all-β conformation of protein was preserved, with the rise in β-sheet from 33.4% to 47%, whereas the tertiary structural stabilization was reflected by a rise in Trp fluorescence indicating compaction of protein.^60^ Lozano et al.^62^ reported the structural and functional stability of enzymes, i.e., α-chymotrypsin in the [C_2_mim][NTf_2_]:1-propanol:H_2_O (85.5:12.5:2 (v/v/v) mixture. The mechanism of stabilization was explained based on the Dupont model of wet ILs.^24,54^ Enzymes reside in the hydrophilic gaps of the network, where the observed stabilization of enzymes could be attributed to the maintenance of this strong network around the protein. The extremely ordered supramolecular structure of ILs in the liquid phase was proposed to act as a “mold” in maintaining an active three-dimensional structure of enzymes in aqueous nanoenvironments, while avoiding the classical thermal unfolding.^62^ Therefore, A-ILCS of hydrophobic ILs form an IL solvent cage around proteins, allowing the stabilization of it against thermal denaturation (Figure 12).

This structure stabilization model however changes significantly with the introduction of ILs with more interactive sites, polarity, or structure slightly similar to that of water. For instance, Falcioni et al.^290^ reported the stability of the conformation and activity of enzyme Subtilisin in A-ILCS of [(OHC_2_)_2_NH_2_]Cl:H_2_O (97.8:2.2, v/v mixtures), which was due to the presence of two hydroxyl groups in the cation that coordinate with the denaturing Cl anion, hence overturning its deleterious effect.^290^

**Figure 13:**
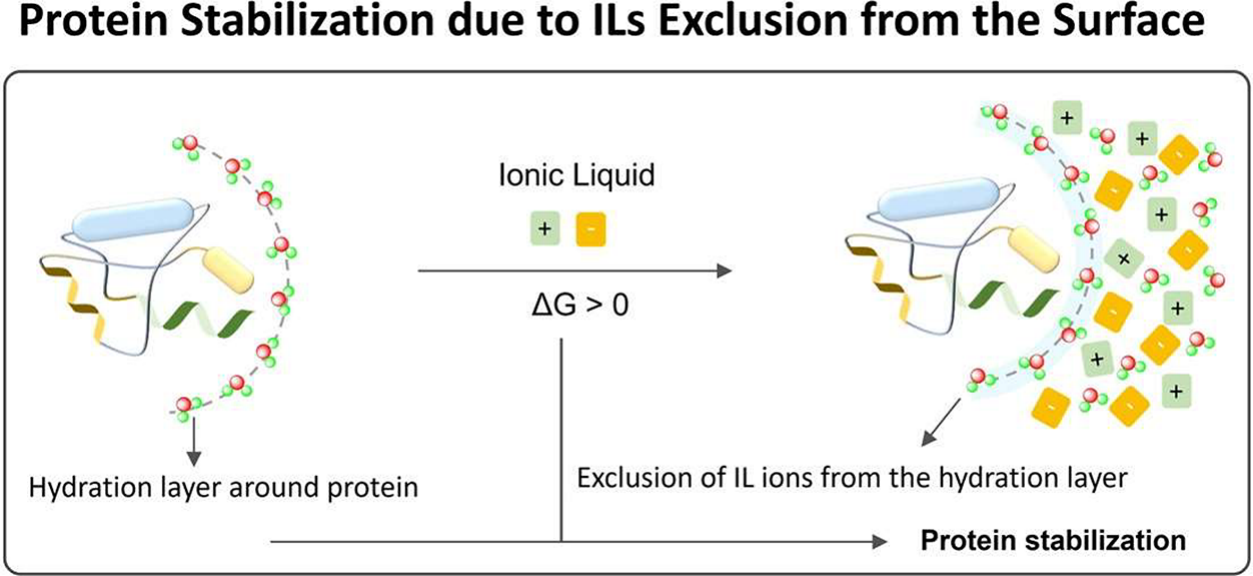
Illustration of the mechanism of protein stabilization by the quaternary ammonium cation family of A-ILCS according to preferential exclusion from protein surface concept.

Venkatesu and co-workers^67,291−295^ investigated the co-solvent effect of quaternary ammonium-based ILs on various proteins, with a special focus on α-chymotrypsin. In most of their reports, they showed the stabilization of proteins by the quaternary ammonium family of ILs, which was explained based on the mechanism of preferential exclusion of ILs ions from the protein surface. This is a phenomenon popular with the stability of proteins by osmolytes according to the osmophobic theory of Bolen^213^ and transfer Gibbs free energy concept (Figure 13).^215,216^

In their first work in the field, Attri et al.^67^ reported higher activity and stability of α-chymotrypsin in ILs: H_2_O (50:50, v/v) A-ILCS of [(C_2_H_5_)_3_NH][OAc] and [(C_2_H_5_)_3_NH][PO_4_] compared to [Bzmim]Cl, [Bzmim][BF_4_], and [(C_4_H_9_)_4_P][Br]. The phenomenon was explained based on unfavorable interactions of quaternary ammonium-based IL ions with the disulfide bonds and backbone of α-chymotrypsin.^67^ The [(C_2_H_5_)_3_NH][OAc]-based A-ILCS was also shown to refold α-chymotrypsin from the quenched thermally unfolded state. Further, Attri et al.^291^ reported that 3 molar of [(C_2_H_5_)_3_NH][OAc] in water attenuates the deleterious effect of urea on the denaturation of α-chymotrypsin due to the H-bond acceptor ability of acetate, strengthening the water-water and water-urea interactions and limiting the urea-α-chymotrypsin H-bonding interactions.^291^ To get deeper insights into the molecular mechanism of IL-protein interactions toward protein stability, the authors investigated the effect of A-ILCS of [Et_2_NH][OAc], [Et_3_NH][OAc], [Et_2_NH][dhp], [Et_3_NH][dhp], [Et_2_NH][HSO_4_], and [Et_3_NH][HSO_4_] on the stability of cyclic dipeptides, namely, cyclo(Gly-Gly), cyclo(Ala-Gly), cyclo(Ala-Ala), cyclo(Leu-Ala), and cyclo(Val-Val)^292^ based on the transfer free energy (Δ*g*_tr_) concept.^215,216^ The positive values obtained revealed unfavorable interactions between ILs and cyclic dipeptides, leading to the stabilization of the native structure of cyclic dipeptides. The authors concluded that peptide bonds, the peptide backbone unit, the alanyl residue, and the valyl residue (containing amide) play a more relevant role in protein folding/unfolding compared to side chains of proteins.^292^ A similar mechanism was proposed for the stabilization of zwitterionic glycine peptides, namely, glycine (Gly), diglycine (Gly2), triglycine (Gly3), tetraglycine (Gly4), and cyclic glycylglycine (c(GG)), with a decreasing order of the *m* value, [(C_2_)_2_NH_2_][HSO_4_] > [(C_2_)_3_NH][OAc] > [(C_2_)_3_NH][HSO_4_] > [(C_2_)_2_NH_2_][OAc] > [(C_1_)_3_NH][OAc]>[(C_1_)_3_NH][dhp].^293^ Attri et al.^294^ also reported stability of succinylated ConA in A-ILCS of [(C_2_)_2_NH_2_][dhp], [(C_2_)_3_NH][dhp], [(C_2_)_2_NH_2_][HSO4], [(C_1_)_3_NH][dhp], and [(C_2_)_3_NH][HSO4] with 50% sodium acetate buffer (v/v).^294^ The studied IL cations failed the Hofmeister series to explain the stability of succinylated Con A, despite being “chaotropic”.^294^ Contrary to this 50% A-ILCS, [(CH_3_)_3_NH]^+^ with a “kosmotropic” anion perfectly follows the Hofmeister series (SO_4_^2–^ > HPO_4_^2–^ > CH_3_COO^–^) in regard to the succinylated Con A stability.^295^ Therefore, when explaining protein stability in A-ILCS based on the Hofmeister series, it is actually the cation series or new organic anions which should follow the order to be considered as part of the new development. Additionally, quaternary ammonium-based ILs cannot be universalized for protein stability and the effect can reverse either by changing the anion or protein structure. For example, quaternary ammonium-based A-ILCS with the OH^–^ anion, *viz* ([(CH_3_)_4_N][OH], [(C_2_H_5_)_4_N][OH], [(C_3_H_7_)_4_N][OH], [(C_4_H_9_)_4_N][OH]): 50% sodium phosphate buffer, destabilized the structure of myoglobin and hemoglobin,^68^ which was due to the direct interaction of OH^–^ with the protein surface, unlike the exclusion effect reported earlier.^291,292^ In contrast, the presence of the hydroxyl functionality was later cited as the reason for the stabilization of Cytochrome c in A-ILCS.^61,63,66^ Therefore, the protein-IL interactions are highly specific and vary from NIL to A-ILCS, with different proteins, different ILs, and particular specific IL-protein combinations.

**Figure 14:**
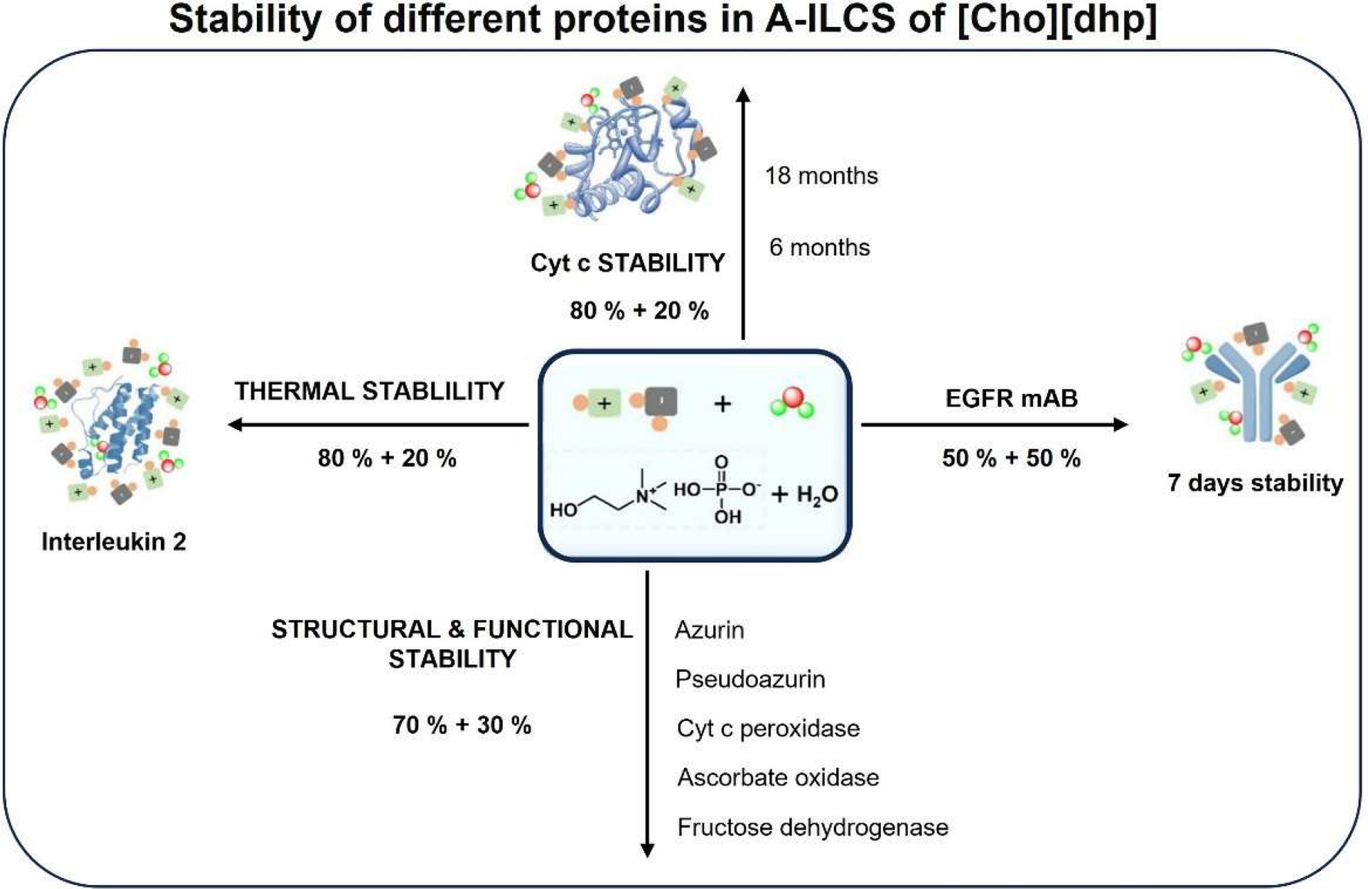
Stability effect of A-ILCS of [Cho][dhp] on different proteins.^61,63,66,296,298,299^

Fujita et al.^61^ introduced the biocompatibility term to ILs and studied the temperature-dependent secondary structure stabilization of Cytochrome c in 80% A-ILCS of [Cho][dhp] and [P_1,4_][dhp] in 20% water. Aqueous mixtures of [P_1,4_][dhp] stabilized Cytochrome c up to 130 °C, compared to 100 °C in mixtures with [Cho][dhp]. The [dhp] anion bears an H-bond donor and an acceptor site, like that of neutral water, being responsible for the high protein stabilization effect. Interestingly, when the water content in A-ILCS was increased up to 80%, denaturation of protein was observed at much lower temperatures (77 and 62 °C for [Cho][dhp] and [P_1,4]_[dhp]). Therefore, as a solute at low concentration, the [dhp] anion acted like a buffer solution in the thermal destabilization of the protein. This work demonstrated the opposite role exerted by IL ions at low and high concentrations of water, in which an optimized amount of water is beneficial to keep the stability of Cytochrome c.^61^ Working on the time-dependent stability of Cytochrome c, Fujita et al.^63^ reported excellent stability of the protein in 80% A-ILCSs (IL:H_2_O, 80:20, v/v) of cholinium-based ILs possessing different anions. The structural and functional activity of the enzyme was explained based on the kosmotropic order of the IL anions: [dhp] > [(C_4_H_9_)_2_ PO_4_] > [OAc] > [Lac] > [CH_3_SO_3_]. Still, the activity observed was highest for [Cho][dhp], which is the most suitable combination of a “chaotropic” cation with a “kosmotropic” anion. Remarkably, the long-term stabilization of Cytochrome c for up to 6–18 months was observed in A-ILCS with 80% [Cho][dhp] in water.^296,63^ This report showed the relevance of A-ILCS for a stable *in vitro* kinetic (long-term) packaging of proteins and TPs. Mazid et al.,^297^ from the same research group (MacFarlane and co-workers), investigated the biological structure and chemical stability of epidermal growth factor receptor monoclonal antibody (EGFR mAb) in cholinium-based buffered IL solutions, namely, [Cho][dhp]:H_2_O (20:80 and 50:50 v/v) stored at room temperature from 7 h to 7 days in the presence of proteinases. The EGFR mAb retained its α-helical structure and activity, as evidenced by successful binding to its cell receptor, while indicating the potential of this mixture for the packing of antibodies. Interestingly, a higher stability was observed at 50% [Cho][dhp] compared to 20% [Cho][dhp], which is in line with Fujita et al.^61,63^ However, both structural stability and functional activity showed a decrease with time. In 2010, Fujita et al.^66^ again showed the relevance of [Cho][dhp]:H_2_O (70:30, v/v) A-ILCS, but now on the enzymatic activity and thermal stability of several metalloproteins in addition to Cytochrome c, namely peroxidase, ascorbate oxidase, azurin, pseudoazurin, and fructose. Overall, this hydrated IL, i.e., [Cho][dhp], has indeed been one of the most promising ILs identified to keep the integrity of protein’s structure.^298^ The stability effect of [Cho][dhp] on different proteins in A-ILCS is illustrated in Figure 14.

The relevance of [Cho][dhp] for protein stabilization proven by Fujita et al.^61,63,66,296^ was further exploited by Weaver et al.,^299^ who investigated in detail the effect of [Cho][dhp] on the thermodynamics, structure, and stability of lysozyme and Interleukin-2 as a TP. Both proteins were found to be soluble in [Cho][dhp] at all tested concentrations, but solubility was limited at 80% [Cho][dhp] in water. Increasing the amount of [Cho][dhp] present in the aqueous protein solution resulted in an increased thermal transition temperature (*T*_m_), without significant changes in the protein’s tertiary structure for both proteins. Binding analysis with the lysozyme using isothermal titration calorimetry (ITC) showed that stability is not induced by the IL binding to the lysozyme. Thermal stabilization at low IL concentrations is due to shielding effects, where the net charge on the surface of the protein plays a key role; at high IL concentrations, stability is more dependent on solution properties. This work is especially promising since it addresses a TP, interleukin-2, paving the way for the use of IL-water mixtures to stabilize other TPs and other biopharmaceuticals. Using calorimetric and spectroscopic analysis Dhiman et al.^300^ reported enhanced thermal transition temperature (*T*_m_) and 2 weeks of room-temperature structural stability of TP, immunoglobulin G (IgG) in A-ILCS of [Cho][OAc] and [Cho]Cl (up to 2.5 mM of ILs in water).^300^ Kumar et al.^301^ also showed the thermal stability of TPs, namely of insulin, in A-ILCS of protic quaternary ammonium ILs (from 20% to 80%), namely, [(C_1_)_3_NH][HSO_4_], [(C_2_)_3_NH][HSO_4_], [(C_1_)_3_NH][dhp], [(C_2_)_3_NH][dhp], and [(C_2_)_3_NH][OAc]. The authors showed that A-ILCS stabilizes insulin in its active monomeric form, revealing the role of compatible quaternary ammonium-based ILs in the packaging of TPs.

Other works regarding A-ILCS of quaternary ammonium-based ILs have shown the stability and enhanced activity of Cytochrome c.^302−306^ For instance, Papadopoulou et al.^302^ reported the stability and a 20-fold increase in the activity of Cytochrome c in [OHC_2_NH_3_][HCO_2_] (60% in water), resulting from the high “chaotropicity” of the cation. Bhakuni et al.^303^ showed the thermal stability and packaging of Cytochrome c for a month, with a 1.5-fold rise in activity in an IL-based medium comprising [Cho][dhp] (50%) and synthetic crowding agents, like Fycol + ethylene glycol + sucrose (5%). Matias et al.^304^ reported the retention of intrinsic redox properties of Cytochrome c in an aqueous solution of [Cho][dhp] and [Cho][MES]. Still, with cholinium-based ILs, Bisht et al.^305^ demonstrated the thermal and thermodynamic stability of Cytochrome in A-ILCS of [Cho][Glu]:water (50:50, w/w) under multiple stresses, such as temperature and H_2_O_2_. The authors reported a 50-fold increase in Cytochrome c activity, thermal stability up to 120 °C, and thermodynamic stability up to 5 months under ambient conditions, thus demonstrating the good packaging ability of the studied A-ILCS toward Cytochrome c. With a different perspective, Takekiyo et al.^306^ studied the cryopreservation effect of aqueous IL solutions of [C_4_mim][SCN] and [C_2_NH_3_][NO_3_] on Cytochrome c, concluding that although these ILs denature the Cytochrome c before and after cooling to −196 °C, >90% of the enzyme activity following cryopreservation was recovered. Overall, it is clear that it is important to analyze the structure of proteins in water after treatment or storage in A-ILCS to know their structural changes.

**Figure 15:**
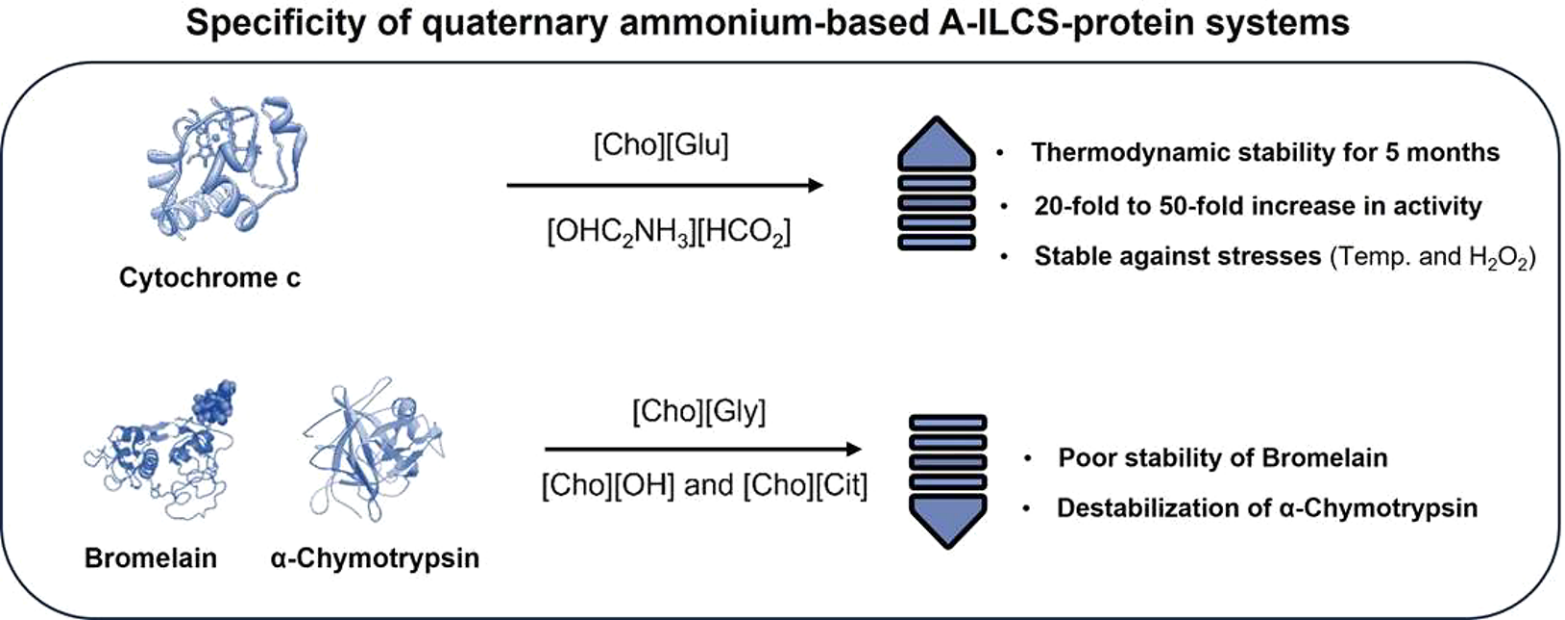
Specificity of quaternary ammonium-based A-ILCS-protein systems.^301−307,68^

The described studies show that aqueous solutions of quaternary ammonium-based A-ILCS in combination with suitable anions allow the efficient packaging of Cytochrome c. However, this notion can change for other proteins with slightly different IL cation–anion combinations. For instance, A-ILCS of [Cho][Gly] led to poor stability of Stem bromelain.^307^ While the authors presumed H-bonding of the glycinate anion with the protein peptide backbone as the main reason for the observed effect, this is in stark contrast with the long-term stability of Cytochrome c in A-ILCS of the same IL, wherein the glutamate anion has more H-bonding sites. We do believe that the observed effect could be due to the specificity of IL-protein systems (Figure 15). Further evidence of specificity is found in the destabilization of α-chymotrypsin by A-ILCS of [Cho][OH] and [Cho][Cit]^308^ and hemoglobin and myoglobin by A-ILCS of [(CH_3_)_4_N][OH], [(C_2_H_5_)_4_N][OH], [(C_3_H_7_)_4_N][OH], and [(C_4_H_9_)_4_N][OH].^68^

Beyond packaging, several other applications of A-ILCS in protein unfolding-refolding, fibrillation, and crystallization were studied. For example, Fujita et al.^309^ reported that [Cho][dhp] with 4 water molecules *per* ion pair showed the best refolding tendency toward the aggregated recombinant cellulase obtained from *Escherichia coli* (CcCel6A). To understand the mechanism of refolding, the authors studied a series of A-ILCS with phosphonium and quaternary ammonium cations combined with bromide, chloride, and dihydrogen phosphate (dhp) anion. These mixtures were restricted to a limited number of water molecules *per* ion pair (3 to 15) on the solubilization and refolding of aggregated Concanavalin A (ConA).^310^ The authors concluded that the solubilization of aggregated ConA in the studied A-ILCS decreases with an increase in the water content, wherein [P_44412_][dhp] and [N_8888_][dhp] were found to be the most effective refolders due to the stronger H-bonding established between the anion and the protein.^310^ Constantinescu et al.^311^ investigated the patterns and equilibrium of unfolding-refolding and aggregation of RNase A in A-ILCS (0.5 to 4 M IL in water) employing the Lumry-Eyring scheme. The A-ILCS of 0.5 M [Chol][dhp] increased the thermal stability of RNase A, wherein the unfolding transition is about 66% completed at 70 °C compared to complete unfolding in an IL-free sample. Moreover, A-ILCS of [Cho][dhp] refolded 5 to 90% of the structure of heat-denatured RNase A, being the system found as the most efficient in suppressing the RNase A oligomerization.^311^ Byrne et al.^312^ improved lysozyme stabilization by mixing two concepts, namely, conventional sugar protection and proton activity of ILs, and reported aggregation protection against reversible folding-unfolding and multiyear (3 years) stabilization of lysozyme in A-ILCS of [CH_3_CH_2_NH_3_][NO_3_]. The solution developed is composed of sucrose (27 wt %), [CH_3_CH_2_NH_3_][NO_3_] (31 wt %), water (20 wt %), and lysozyme (22 wt %). However, a small decrease in the denaturation enthalpy (*T*_m_ = 74 °C), from 20.70 J·g^–1^ to 19.72 J·g^–1^, was observed up to the third cycle. When subjected to DSC measurements, no changes in the unfolding enthalpy (dH_U_) were observed for cold-stored lysozyme, whereas a 1/3 decrease in dH_U_ was observed, indicating a 2/3 loss of protein to aggregation or hydrolytic decomposition. This work represents a significant development because protein samples usually stored in a refrigerator begin to aggregate after 7 days of storage. Therefore, the retention of 25% of the protein structural integrity after 3 years of incubation at room temperature is one outstanding achievement that should be further investigated, and the respective underlying molecular-level mechanism should be depicted.

Further understanding of the unfolding-refolding mechanism was sought by investigating the effect of proton activity (PA) of ILs on the denaturation temperature of lysozyme and RNase A, as a solution property parallel to that of pH in aqueous solution in the form of a two-state cooperative model (Native ↔ Unfolded).^313^ The authors showed that the unfolding temperature is altered by changing the PA, like what happens when altering the pH. An increase in the thermal stability of both the proteins was observed with an increase in the δ (N–H) shift. Finally, the authors developed a refolding index (RFI) metric to assess the stability of folded biomolecules in different solvent media and demarcated high RFI zones in hydrated PIL media using RNase A and lysozyme as model proteins.^314^ Wijaya et al.^315^ also used pH as a parameter to understand the effect of A-ILCS of [C_2_NH_3_][NO_3_] and [OHC_2_NH_3_][NO_3_] in 4–17% on the structure and activity of lysozyme. They found that the enzyme retains its α-helical structure, except in the acidic pH, and showed functional activity in the entire pH range (0–11) in the [C_2_NH_3_][NO_3_]-water mixture and from pH 4–11 in the [OHC_2_NH_3_][NO_3_]-water mixture. This is an important work considering that proteins are susceptible to pH-induced conformational changes. Mann et al.^316^ reported refolding of heat-denatured lysozyme in A-ILCS of [C_2_NH_3_][HCOO] and [C_1_OC_2_NH_3_][HCOO] at 25 wt %, where the A-ILCS of [OHC_2_NH_3_][HCOO] has shown to stabilize lysozyme against unfolding at high temperature due to strong H-bonding.^316^

**Figure 16:**
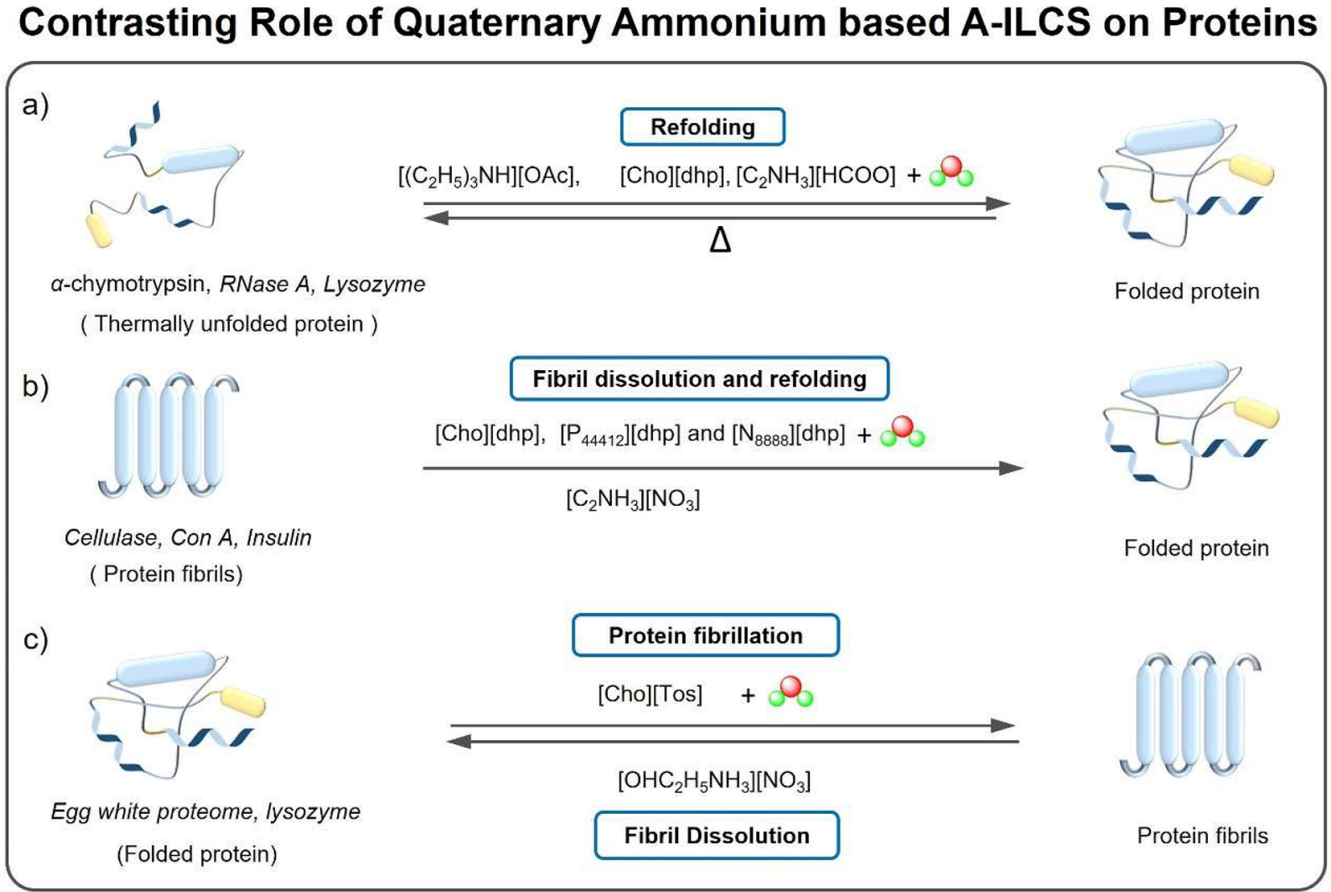
Contrasting role of quaternary ammonium-based A-ILCS on protein refolding and fibril dissolution/promotion.

Many proteins are prone to aggregation in an aqueous solution since the fibrillar state of a protein is the most thermodynamically stable, corresponding to its global energy minima. The best biological examples in this field are β-Amyloid, tau, and α-synuclein, responsible for neurodegenerative diseases, like Alzheimer’s and Parkinson’s diseases. Accordingly, A-ILCSs have been used as well to suppress protein fibrillation. Takekiyo et al.^317^ reported the suppression effect of insulin amyloid formation using aqueous solutions of the following ILs (from 0 to 30 mol %): [C_4_mim][SCN], [C_2_NH_3_][NO_3_], and [C_3_NH_3_][NO_3_]. Notably, at 30 mol % of IL, [C_3_NH_3_][NO_3_] showed a protective effect on the monomeric form of the protein. Byrne et al.^152^ also reported the fibrillation of lysozyme in PIL aqueous solutions and subsequent dissolution with the restoration of activity. The authors fibrillated the lysozyme with 80% NH_4_HSO_4_·20H_2_O and then redissolved the fibrils by the addition of [C_2_H_5_NH_3_][NO_3_], [(C_2_H_5_)_3_NH][CH_3_SO_3_], and [(C_2_H_5_)_3_NH][TfO]. Overall, [OHC_2_H_5_NH_3_][NO_3_] proved to be the most efficient IL in solubilizing the fibrils as it restored 72% of the activity, thus reinforcing the potential of this IL in biomedical applications targeting neurological diseases. However, besides the negative effects of protein fibrils, they can be beneficial when employed as biomaterials for applications in biotechnology, for example as a 3-D matrix for cell-grown and nanomedicines delivery. However, preparing fibrils makes use of pure and expensive proteins, further having long synthesis kinetics. Bharmoria et al.^318^ overcame this drawback by investigating the anion-specific interactions of ILs with the protein, allowing the instant fibrillation of the aqueous egg white proteome using an adequate IL, [Cho][Tos] (0.1 to 1 M in water). This IL contains interaction sites for ionic, hydrogen bonding, and π–π stacking interaction. Using several experimental techniques and computational studies, they found that H-bonding and hydrophobic interactions occurring between the IL and proteins are the driving forces to induce fibrillation, with additional beneficial effects afforded by ILs with aromatic anions by allowing the establishment of π···π interactions. The prepared fibrils were investigated for cytocompatibility by exposing the mouse fibroblast L929 cells to fibrils using a live–dead assay. The cells perfectly retained their membrane integrity and morphology, thus indicating cytocompatibility and possible application as a matrix for cell growth in biotechnology. The contrasting role of A-ILCS as fibril dissolving agents and fibril promoters, which is achievable due to the ILs’ designer solvents nature, is presented in Figure 16.

**Figure 17:**
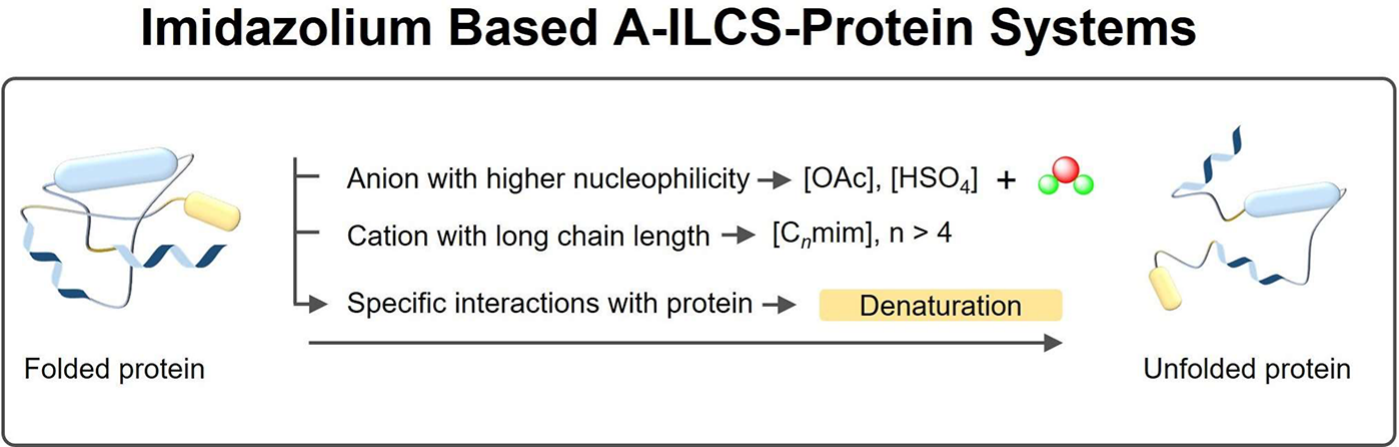
Illustration of the main conclusions obtained from imidazolium-based A-ILCS on protein denaturation and unfolding.

Beyond the quaternary ammonium family of ILs, several hydrophilic imidazolium-based A-ILCS were investigated to improve protein stability, denaturing/renaturing, and crystallization. In this field, Takekiyo et al.^319^ studied the effects of the nano heterogeneous structure of [C_4_mim][NO_3_] on protein conformation in water. Unfolded state of lysozyme was observed at 6 M [C_4_mim][NO_3_] due to specific interactions of the anion with lysozyme, and a partially globular state was observed at 10 M [C_4_mim][NO_3_] due to the reduced hydration of unfolded lysozyme.^319^ Yoshimura et al.^320^ reported the cryoprotection effect of A-ILCS of [C_4_mim]Cl and [C_2_NH_3_][NO_3_] from 0 to 30 mol % on lysozyme. A small loss in structure and activity of lysozyme occurred upon cooling to −196 °C, which was reversed upon returning to ambient temperature. Tavares et al.^321^ reported the maintenance of activity of laccase in A-ILCS of [C_2_mim][MDEGSO_4_], [C_2_mim][C_2_OSO_3_], and [C_2_mim][C_1_SO_3_] (10 to 75% in water) at pH 9.0, in addition to just a 10% loss in activity for 1 week of incubation in A-ILCS of [C_2_mim][MDEGSO4].^321^ Ferdjani et al.^322^ reported the activity and stability of a β-glycosidase (*Thermus thermophilus*) and two α-galactosidases (*Thermotoga maritima* and *Bacillus stearothermophilus*) in A-ILCS of [C_1_mim][C_1_OSO_3_] and [(C_1_)_2_mim][C_1_OSO_3_] (30–80%). Kim et al.^323^ reported a higher activity of *Candida antarctica* lipase B in A-ILCS of [C_4_mim][TfO] due to the stabilization of enzyme active sites. This concept was further vindicated by Nordwald et al.,^324^ who engineered the active site of lipase A (lipA) from *Bacillus subtilis* to increase resistance against 50% [C_4_mim]Cl in water.^324^ Using the SANS technique, Heller et al.^71^ reported dimeric to monomeric conformational transition of the green fluorescent protein (GFP), with a decrease in thermal denaturation in 25 and 50 vol % of [C_4_mim]Cl. Further analysis of IL-GFP interactions using a multitechnique approach in 1 M [C_4_mim]Cl, [C_4_mim][OAc], [C_4_C_1_py]Cl, [C_4_C_1_py][TfO], and [C_4_C_1_py][OAc] revealed direct interactions of IL ions with the protein surface, dominated by the type of anion to induce secondary/tertiary structural changes.^325^ In this direction, Lou et al.^326^ investigated the role of imidazolium IL paired anions ([BF_4_], [HSO_4_], Cl, [NO_3_], and [OAc]) on the structure and function of papain in A-ILCS (15% ILs in water). The authors concluded that the anion has a high impact on the structure, activity, and enantioselectivity of papain, since it was more stable in [C_*n*_mim][BF_4_] (*n* = 2–6) containing systems compared to [C_4_mim][HSO_4_], [C_4_mim]Cl, [C_4_mim][NO_3_], or [C_4_mim][OAc]. The strong nucleophilicity of lower stabilizing anions caused higher interactions with positively charged sites on the enzyme and breaks internal H-bonding or forms new hydrogen bonds that perturb the enzyme structure. Additionally, due to the strong acidity, [C_4_mim][HSO_4_] broke the disulfide bond between Cys-56 and Cys-95 located in the helical domain of the papain molecule, leading to the denaturation of the protein.^326^ Jha et al.^327^ investigated the effect of the alkyl side chain length of imidazolium-based ILs; [C_*n*_mim]Cl (*n* = 2, 4, 6, 10) from 0.01 to 1.5 M was applied to address the structural stability of Stem bromelain. It was concluded that their destabilizing effect increases with the cation alkyl chain length and consequent increase in hydrophobic interactions with the protein backbone.^327^ Figueiredo et al.^328^ reported that the hydrating ability of the IL anion and hydrophobicity of the cation dominates the stability of a small α-helical protein, when studied in 240 mM to 1000 mM IL ([C_4_mim]Cl, [C_4_mim][dca], [C_2_mim], and [C_2_mim][dca]) solution in water. Weakly hydrating anions, like [dca], dehydrate the positively charged residues on the protein backbone, whereas slightly more hydrophobic cations like [C_4_mim]^+^ cause higher denaturation. A summary of the main conclusions obtained from imidazolium-based A-ILCS on protein denaturation and unfolding is presented in Figure 17.

Many reports used the internal fluorescence of tryptophan as a marker to investigate protein unfolding or denaturation. However, it is not a confirmatory technique since an increase or decrease in tryptophan fluorescence can happen due to multiple factors, like the inner filter effect and change in solvent polarity, other than quenching due to IL binding.^69^ Therefore, it is important to use advanced characterization tools such as SAXS, SANS, and fluorescence correlation spectroscopy to obtain correct information on IL-protein interactions. For example, from SANS and SAXS analysis, Baker et al.^70^ reported the denaturation of Cytochrome c and HSA to random coil conformation in 50% [C_4_mim]Cl in water, behaving like classical denaturants such as Guanidinium-hydrochloride and urea. In a series of works, Bhattacharya and co-workers^112,113,329^ exploited the fluorescence correlation spectroscopy technique to understand the protein dynamics in A-ILCS. Sasmal et al.^329^ reported the opposing effect of [C_5_mim]Br on conformational dynamics (unfolding and refolding) of HSA when in native or denatured form by fluorescence correlation spectroscopy (FCS). FCS is also a useful tool to measure hydrodynamic radii (*R*_h_) of colored protein solutions whose absorption/emission interfere with the excitation laser wavelength of the dynamic light scattering (DLS) instrument. For example, A-ILCS of [C_5_mim]Br showed contrasting interaction behavior with human serum albumin (HSA), wherein it unfolded the native conformation and refolded the denatured conformation of HSA.^329^ During unfolding-refolding, the dynamics of the protein side-chain changed faster at τ_R_= 3–40 μs, whereas interchain interactions occurred at a slow time scale of τ_R_ = 100–300 μs. The authors further investigated the change in the microenvironment of HSA in the presence of 1.5 M [C_5_mim]Br and 6 M GdnHCl from femtosecond up-conversion using 7-diethylamino-3-(4-maleimidophenyl)-4-methylcoumarin (CPM) as a covalently attached probe to cys-34. They found a decrease in solvation time (τ_s_) of HSA from 650 to 260 ps (∼2.5 times) and 60 ps (∼11 times) in the presence of 1.5 M IL and GdnHCl, respectively, due to protein unfolding. Upon refolding analysis, a ∼2-fold faster solvent relaxation (τ_s_ ≈ 30 ps) compared to GdnHCl denatured HSA was found.^112^ Hence, different conformations of proteins can be easily identified by getting information on their solvation using FCS, which is an advance relative to the conventionally known *R*_h_ analysis-based DLS technique.^112,113,329^ A similar behavior was observed during FCS analysis of Cytochrome c in A-ILCS of [C_5_mim]Br using Alexa Fluor 488 as probe.^113^ The different relaxation of solvent around different conformations of Cytochromes could help to identify native, molten globule (MG-I and MG-II), and refolded conformation of Cytochrome c.^114^ These studies also indicate that IL-based refolded proteins never attain the native conformation since they show different solvation dynamics and may operate differently when used in different applications.

**Figure 18:**
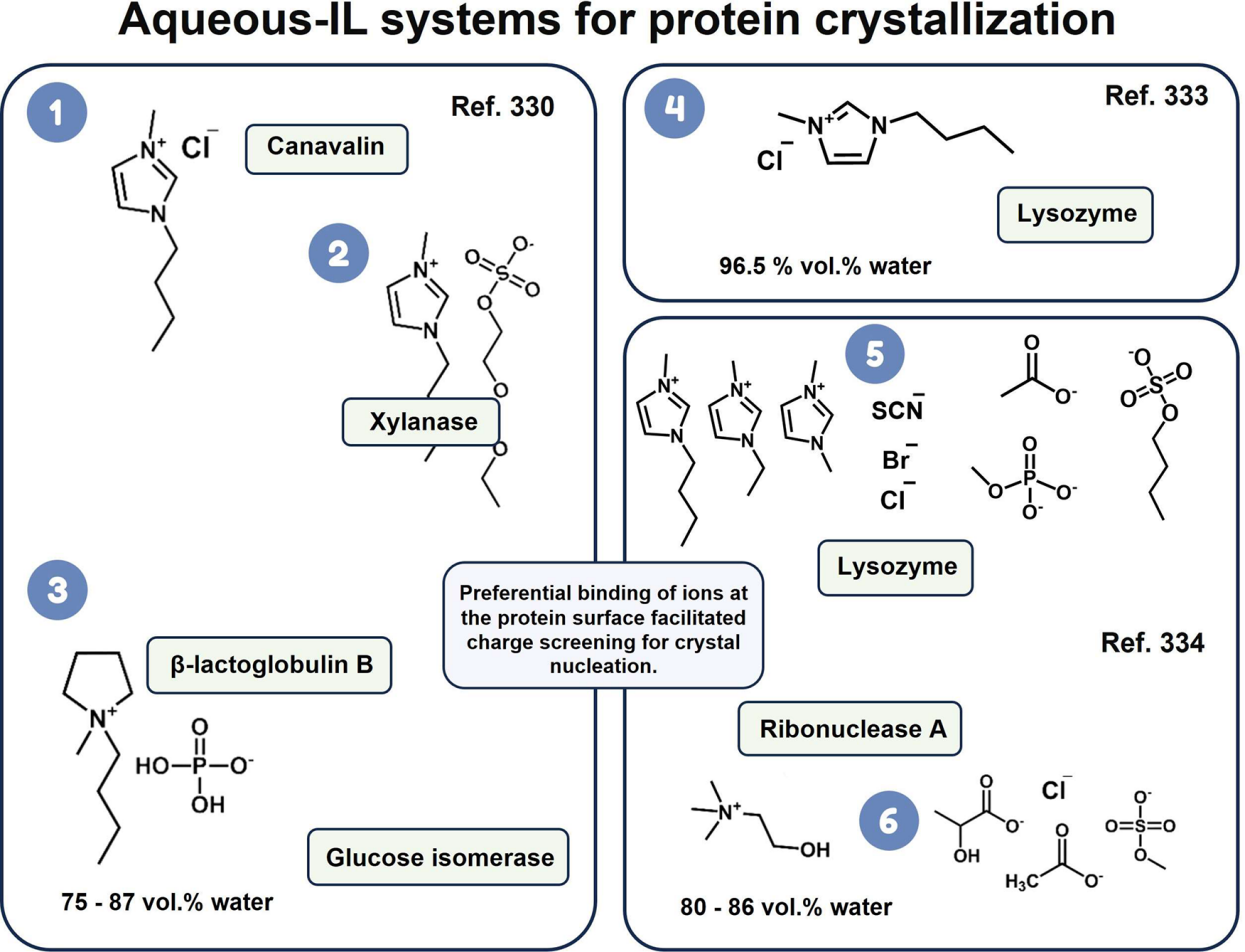
Most suitable water-IL systems for protein crystallization.^330,333,334^

While A-ILCSs are important for protein packaging, another application of high interest is to promote protein crystallization by tuning the properties of water. Protein crystals hold high relevance when elucidating the correct structure of the protein. If IL ions are trapped during crystallization, it would give correct information about their binding sites. The most suitable water-IL systems reported are depicted in Figure 18.

Pusey et al.^330^ studied the crystallization tendency of A-ILCS comprising 0.4 M [C_4_mim]Cl, [C_4_mim][MDEGSO_4_], and [P_1,4_][dhp] on the crystallization of canavalin, β-lactoglobulin B, xylanase, and glucose isomerase. All protein-IL combinations produced at least one crystal, in which the ILs were proposed to act as a primary precipitant agent.^330^ Hekmat et al.^331^ reported enhanced lysozyme crystallization kinetics with reduced polymorphism in A-ILCSs (2.5 to 10% of ILs in water) of [OHC_2_NH_3_][HCO_2_], [C_2_NH_3_][NO_3_], [(CH_3_OC_2_H_5_)_2_NH_2_][OAc], [OHC_2_H_5_(CH_3_)_2_ NH] [Gly], and [Cho][dhp]. Soft anions such as formate or glycolate are superior to ILs with hard anions, like nitrate, in promoting crystallization. Wang et al.^332^ reported enhanced thermal resistance of lysozyme crystals prepared in 1% [C_4_mim]Cl, which is due to the change in lysozyme-lysozyme molecular interactions. This IL promoted the nucleation of crystals with small size, lower surface free energy, and improved morphology. Chen et al.^333^ reported that 3.5% [C_4_mim]Cl in water lowered supersaturation conditions to promote nucleation of lysozyme at ambient conditions. The X-ray diffraction pattern of the crystals showed the incorporation of the imidazolium cation into the crystal framework of the protein, where it is bound to the N and C atoms of Trp62, the C atoms of Trp63, and the O atom of Asp101.^333^ This is solid evidence of specific molecular interaction between IL ions and amino acid residues of proteins.

**Figure 19:**
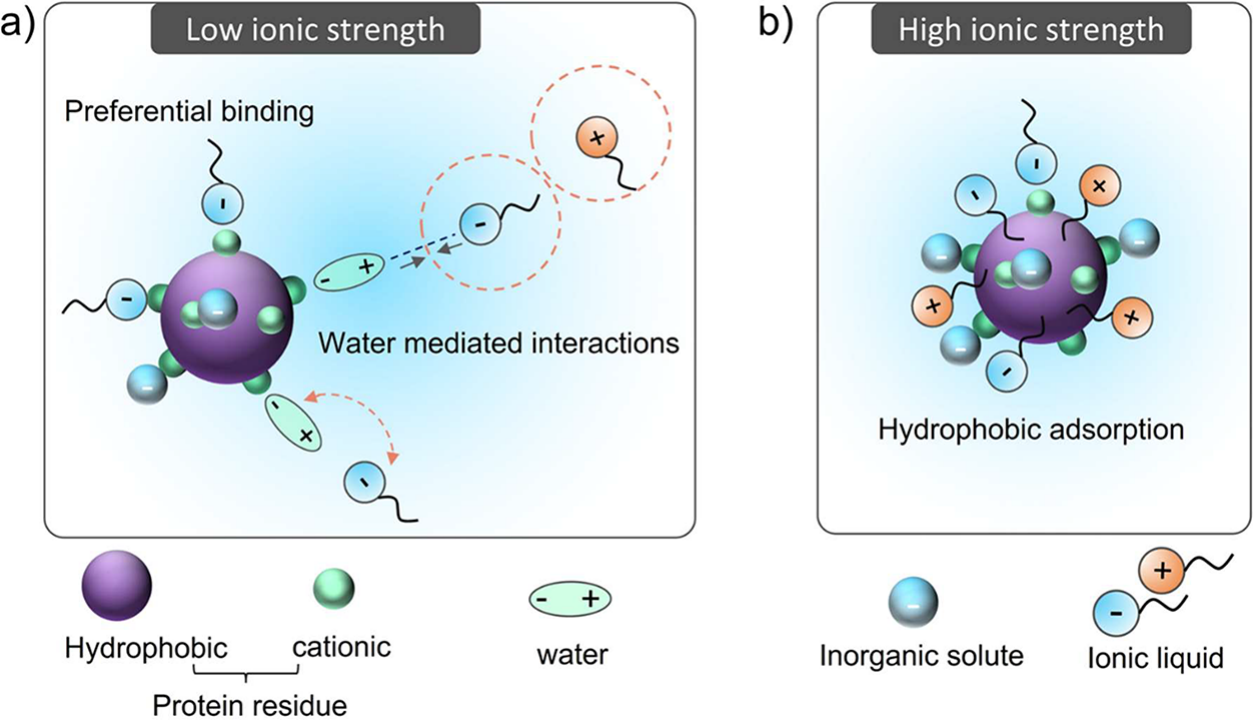
Schematic representation of interactions of a protein containing hydrophobic and cationic surface groups with its surroundings (IL ions, water, and inorganic ions). At low ionic strength, preferential binding of weakly hydrated anions to the cationic residues causes charge screen, resulting in the salting-out of the protein. The water-mediated interactions: electrostatic stabilization of hydration (salting in) and exchange of water molecules hydrating the protein with positively (salting-out) and negatively (salting-in) hydrated ions. At high ionic strength, nonspecific adsorption of weakly hydrated ions to the hydrophobic surface reduces the protein-water interfacial tension resulting in the salting-in of the protein. Adapted with permission from ref (334). 2012 The Royal Society of Chemistry.

In depth mechanisms of the IL on protein’s crystallization was further investigated by Kowacz et al.,^334^ who studied the effect of the IL type and its concentration (0.0625 to 1 M) on the crystallization of lysozyme and RNase A. The ILs used were [C_1_mim][C_1_OPO_3_], [C_2_mim]Cl, [C_2_mim]Br, [C_2_mim][SCN], [C_2_mim][OAc], [C_2_mim][C_2_SO_3_], [C_2_mim][C_4_SO_3_], [C_4_mim][Cl, [C_4_mim][OAc], [Cho]Cl, [Ch][OAc], [Ch][Lac], and [Ch][C_1_SO_3_]. The authors explained that IL-protein molecular interactions drive protein crystallization, based on a combined effect of the intrinsic properties of ILs and their ionic strength. The relative salting-in/-out ability of ILs to assist crystallization changes upon increasing or decreasing their ionic strength. At low ionic strength, when the electrostatic interactions predominate, the ions screen the charge on the protein surface by preferential binding to avoid repulsive interactions between the similarly charged protein molecules, thus facilitating protein nucleation to promote crystallization. At high ionic strength, the IL ions influence the crystallization by altering the protein–water interfacial tension therein. ILs stabilize the protein in solution by lowering the protein–water interfacial tension via hydrophobic adsorption on the protein surface. As a result, anions that exert the highest salting-out effect at low ionic strength become the most prominent salting-in agents when hydration forces start to dominate at high ionic strength, thus inhibiting the seeding of protein crystallization.^334^ The molecular mechanism exerted by ILs on protein crystallization at low and high ionic strength is depicted in Figure 19.

Further evidence of the molecular mechanism of ion-protein interactions was obtained from simulation studies of A-ILCS-protein systems. From MD studies, Jaganathan et al.^335^ confirmed stable compaction of Cytochrome c, solvated by [C_2_NH_3_][NO_3_] (0.1 to 1 mM in the water), due to H-bonding between [C_2_NH_3_][NO_3_] and water, and electrostatic interactions between [C_2_NH_3_][NO_3_] and charged residues on the protein surface. This rationale supports the experimental results of A-ILCS-protein interactions for the quaternary ammonium family of ILs. Burney et al.^336^ reported stability of lysine to glutamate surface engineered *Bos taurus* α-chymotrypsin and *Candida rugosa* in A-ILCSs of 20% [C_4_mim]Cl and [C_2_mim][C_2_OSO_3_] in water, which was due to the clustering of cation and decreased anion interaction with the protein surface. The role of anion-specific interactions was further studied by Latif et al.^337^ on the dynamic properties of *Candida antarctica* lipase B and *Candida rugosa* lipase solvated by [C_4_mim[PF_6_], [C_4_mim[BF_4_], [C_4_mim]Cl, [C_4_mim[TfO], and [C_4_mim[Tf_2_N] (10% to 50% ILs in water). The ILs destabilized the enzymes by stripping off water from the hydration layer, in the order [Tf_2_N] < [PF_6_] < [TfO] < [BF_4_] < Cl. Li et al.^338^ reported competitive binding of [C_4_mim]^+^ at the active site of cellulase cellobiohydrolase I (CBH I) at high [C_4_mim]Cl concentration, inducing the inactivation of the enzyme. Manna et al.^339^ also reported a loss in activity of endoglucanase Cel12A from *Rhodothermus marinus* (RmCel12A) in 20%, 40%, and 60% aqueous solution of [C_2_mim][OAc] due to the intrusion of [C_2_mim] cation into the active site of the enzyme, thus affecting the dynamic motions around the active site.^337^ The cation-specific IL-protein interactions were further observed with A-ILCS of [C_4_mim]Cl on three α-helix bundles (the domain of protein A from *Staphylococcus aureus* (BdpA)). The authors cited the preferential accumulation of the [C_4_mim] cation at the protein surface as the reason for protein stabilization in 25%, 45%, and 65% of [C_4_mim]Cl in water. The accumulated [C_4_mim] cation reduces the H-bonding between water and the protein by displacing the water molecules from the protein surface, hence stabilizing the backbone hydrogen bonds.^339^ Using enhanced molecular dynamics simulations, Jaeger et al.^340^ reported closeness of HSA solvated in concentrated A-ILCS of [C_4_mim][BF_4_] and [Cho][dhp] with its crystal structure studied on time scales of hundreds of nanoseconds. Moreover, HSA solvated in 20% [Chol][dhp] in water was found to have similar structural stability to that found in water. The MD simulation studies thus support the specificity of IL-protein interactions for both stability and destabilization in A-ILCS.

#### 3.2.1 Mechanisms of Interaction

From the discussed works involving A-ILCS and proteins, three major mechanisms of interaction have been found to rule the protein’s stability: (1) hydrophobic solvation; (2) dual-hydrogen-bonding; and (3) preferential exclusion of ions from the protein surface.

##### 3.2.1.1 Hydrophobic Solvation

When considering hydrophobic ILs like [C_4_mpy][Tf_2_N], [(C_2_)_2_mim][Tf_2_N], [C_2_mim][Tf_2_N], [C_4_(C_3_)_3_N][Tf_2_N], [C_4_(C_1_)_3_N][[Tf_2_N], and [C_2_(C_1_)_2_C_3_N][[Tf_2_N], the stabilization of proteins is a result of their rigid solvation in their compact form via hydrophobic interactions and molding into the polar domains of A-ILCS (Figure 12).^59,60,62^ Solvation is driven by hydrophobic interactions with the hydrophobic patches on the protein surface. The water molecules dissolved in [Tf_2_N]-containing ILs behave as free water, interacting via H-bonds with the anions of the ILs, thus providing the desired aqueous-like microenvironment for protein solvation. Going by the secondary structure, three of these proteins (Lysozyme, CAL B, α-Chymotrypsin) possess α+β, and each one possesses α/β and all-β secondary structural conformation. Therefore, these proteins already contain a significant amount of β structure with lesser structural flexibility, which might be assisting in their stable compaction in hydrophobic ILs.

##### 3.2.1.2 Preferential Exclusion

Preferential exclusion has been particularly observed in the quaternary ammonium family of ILs.^67,291−295,301^ The stabilization is caused by unfavorable interactions of the IL ions with the protein surface, due to which ions get excluded, thus causing stabilization by a phenomenon similar to the osmophobic theory of Bolen^213^ and the transfer Gibbs free energy concept (Figure 13).^215,216^ In such cases, IL ions behave similarly to normal osmolytes in preserving the protein structure.

##### 3.2.1.3 Hydrogen Bonding

**Figure 20:**
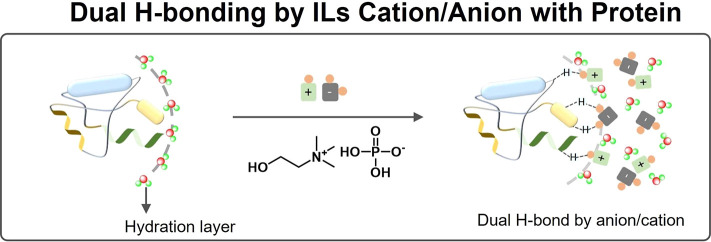
Mechanisms of dual hydrogen bonding, such as with the [dhp] anion leading to higher stabilization of the protein structure.

Hydrogen bonding between IL ions and the protein surface has accounted for both protein stability and destabilization. For example, the most promising IL identified in the set of works considered is [Cho][dhp], which stabilized, among other proteins, the structurally flexible all-α protein Cytochrome c for 18 months.^61,63,66,296,298^ The [dhp] anion was shown to interact with proteins both by donating and accepting protons through hydrogen bonding.^61,63^ Therefore, the water-like dual H-bonding character of [dhp] anion provides a beneficial environment to proteins, whereas the rigid matrix of the IL prevents aggregation-induced denaturation by restricting proteins at specific locations in the ionic matrix (Figure 20).

These restrictions in the ionic matrix limit the protein’s diffusion and protein–protein interactions, which are the major cause of their aggregation. The stabilization effect of [Cho][dhp] is not restricted to Cytochrome c only and has also been applied to stabilize lysozyme^299^ and refolding of RNase A^311^ and cellulase,^309^ thus showing its broad-range application for proteins of different secondary structures. In contrast, anions other than [dhp], but still carrying hydrogen bond sites, such as [OAc], [CH_3_SO_3_], Cl, and [OH],^68−70,305,319^ have been reported to destabilize the protein structure. This is because these anions do not contain dual H-bonding donor sites like [dhp], and they mostly contain H-bond acceptor sites. In these ions, the ionic interaction with the cationic sites on proteins results in the disruption of the existing hydrogen bonding network of proteins to cause unfolding. Like [dhp], the [HSO_4_] and [Lac] anions contain dual H-bonding sites, but each one for H-bond donation and H-bond acceptation, which could be the possible reason for their denaturation effect on proteins.^326^ Interestingly, the high acidity of [HSO_4_] anions was also cited as the reason for breaking the disulfide bonds of the protein papain, inducing denaturation at high concentration (15% in buffer solution);^326^ however, it should be noted that [dhp] is also very acidic and stabilizes proteins.^61,63^ These results show that it is not just cation-protein or anion-protein interactions, but also the cation-anion interactions on the protein surface, that govern the overall effect on the stability of proteins in A-ILCSs.

### 3.3 Ionic Liquids as Adjuvants

Going further into the dilution of ILs in water, in which their structure and solution properties vary significantly, leads to the ILs here defined as adjuvants (concentrations lower than 2%). It should however be kept in mind that even in dilute solutions, ILs do not exist in the form of isolated ions as conventional inorganic electrolytes, but rather in associative forms, such as triple ions, contact ion pairs, solvent shared ion pairs, and loose ion pairs.^24,27^ These forms are reflected in a different interaction behavior with proteins than that observed with typical high-charge density salts. In the field of using ILs as adjuvants (A-ILAS), the systems have been studied in pursuit of understanding the mechanisms of interaction of ILs with proteins, based on Hofmeister effects, and other noncovalent interactions for protein stability, activity, fibrillation, etc.

Constantinescu et al.^341^ investigated the thermal denaturation tendency of ILs on RNase A and formulated a Hofmeister series in comparison to inorganic salts at pH 7.0. The cation series of Hofmeister toward RNase A denaturation follows the order K^+^ > Na^+^ > [C_1,1,1,1_N]^+^ > Li^+^ > [C_2,2,2,2_N]^+^ > [C_2_mim]^+^ > [C_4_mpyrr]^+^ > [C_4_mim]^+^ > [C_3,3,3,3_N]^+^ > [C_6_mim]^+^ > [C_4,4,4,4_N]^+^, whereas the order of anions is [SO_4_]^2–^ > [HPO_4_]^2–^ > Cl^–^ > [C_2_OSO_3_]^−^ > [BF_4_]^−^ ≈ Br^–^ > [C_1_OSO_3_]^−^ > [TfO]^−^ > [SCN] > [N(CN)_2_]^−^ > [Tf_2_N]^−^. In comparison to inorganic cations and with the exception of [C_1,1,1,1_N]^+^, all organic cations destabilize RNase A despite their chaotropic nature, which is against the notion of the modern Hofmeister series wherein chaotropic cations are considered as protein stabilizers (Figure 3).^91−95^ Accordingly, there are forces beyond hydration effects which dictate IL-protein interactions in diluted aqueous solution. Weibels et al.^342^ investigated these forces by the ion specific activity analysis of yeast alcohol dehydrogenase in dilute aqueous solutions of ILs. The authors found hydrophobic interactions as a key factor governing the decreased activity of the enzyme, with the following Hofmeister series of cations and anions: Cl^–^ ∥ Br > [C_2_OSO_3_] > [TfO] > [BF_4_] > [dca] > [SCN] and Na^+^ > [Me_4_N]^+^ > [Chol]^+^ > [C_2_mim]^+^ > [Et_4_N]^+^ > [Bu_4_N]^+^ > [Gdm]^+^ > [C_4_mim]^+^. Kumar et al.^343^ performed comparative analysis of the Hofmeister series of anions with sodium and the [C_4_mim]^+^ cation on α-chymotrypsin. The ILs studied were [C_4_mim][HSO_4_], [C_4_mim][SCN], [C_4_mim]I, [C_4_mim]Br, and [C_4_mim][OAc]. The comparative Hofmeister order of sodium- and [C_4_mim]-paired anions based on the stability against thermal denaturation follows the order SO_4_^2–^ > Br^–^ > I^–^ > SCN^–^ > [OAc]^−^ > Cl^–^, and [OAc]^−^ > Br^–^ > Cl^–^ > HSO_4_^–^ > SCN^–^ > I^–^. The authors concluded that the Hofmeister anions combined with the sodium cation were a complete denaturant for the CT structure. On the other hand, a combination of the same anions with [C_4_mim]^+^ presented the reverse effect on the CT native structure. Therefore, the stabilization/destabilization effects may vary for cation/anion combination, protein, and pH.

Zhao et al.^18^ have reviewed the ion-specific effect of ILs toward protein stabilization/enzyme activity and concluded that in dilute aqueous-IL solutions, with several exceptions, the ion specificity of many enzymatic systems is in line with the traditional Hofmeister series/kosmotropicity; however, the specificity in concentrated or neat ILs is determined by β, the nucleophilicity of anions, hydrophobicity of IL, and other factors.^18^ Kumar et al.^344^ also reviewed the Hofmeister effect of ILs on proteins and concluded that the interactions are dominated by ion specificity and solvent environment related to H-bond disruption, nonpolar interactions, and electrostatic effect to stabilize or destabilize the protein. The bottom line is that the Hofmeister-type effect of ILs is inconsistent in the literature and cannot be generalized. This trend may be because ILs simply do not follow the concept on the grounds of hydration effects alone. Beyond the Hofmeister effect, the preferential electrostatic binding of [C_4_mim]^+^ on α-chymotrypsin and *Candida rugosa* lipase,^345^ and [Amim]^+^ on Hemoglobin^346^ surface, with exclusion of the counterion Cl into the bulk solution, was reported as the reason for protein conformation stability. Similarly, double layer clustering of [C_6_mim]Cl and [C_8_mim]Cl at the protein–water interface was reported as the reason for stable dispersion of proteins, as verified with BSA, HSA, IgG, β-Lg, and Gel-B around their isoelectric point, pH 5.0, in an electrolytic solution of IL.^347^ The surface selective binding of IL molecules to proteins screens their surface charge, leading to pH-independent dispersion stability by arresting their protonation and deprotonation in aqueous solution. This notion is very similar to the Poisson–Boltzmann, Debye–Hückel, and DLVO theories of colloidal stability of proteins in inorganic salt solutions.^197−201^ Therefore, these theories do find validation with organic salt solutions in the form of ILs as well.

**Figure 21:**
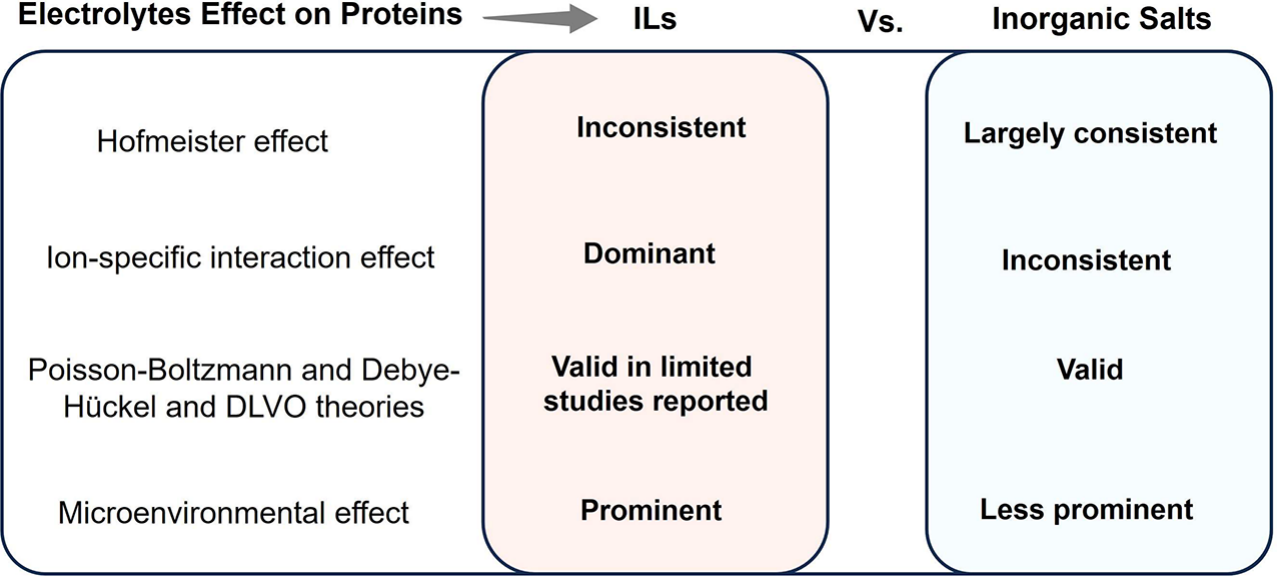
Comparative effects of ILs versus inorganic salts as electrolytes on proteins.

Information availed from colloidal stability of proteins is limited to aggregation-induced destabilization effects, whereas molecular level information on IL-protein interactions could be obtained from spectroscopic assays or docking studies. For example, using small-angle neutron scattering, Heller et al.^348^ reported that the thermally driven unfolding and aggregation of HSA by GdnHCl share a common pathway with [C_4_mim]Cl and [C_4_mim][OAc], wherein [C_4_mim]^+^ and Gdn^+^ cations associate with the surface of the protein to induce more attractive protein–protein interactions and conformational changes. In these, the anion controls the temperature of unfolding. By combining fluorescence and docking studies, Shu et al.^349^ showed that cationic imidazolium moieties of ILs, viz. [C_4_mim][NO_3_], [C_4_mim]Cl, and [(C_4_)_2_im]Cl, enter the subdomains of BSA and interact with the hydrophobic residues of domain III to induce Trp quenching. Huang et al.^350^ reported static quenching of BSA fluorescence by ILs according to the following order: [C_8_mim]Cl > [C_6_mim]Cl > [C_4_mim]Cl. ILs interacting with tryptophan (Trp) and tyrosine (Tyr) residues lead to changes in the structure and internal hydrophobic conformation of BSA. An illustration of the comparative effects of ILs versus inorganic salts as electrolytes on proteins is shown in Figure 21.

In a series of studies, Fan’s research group^351−356^ exploited the internal fluorescence of tryptophan as a tool to investigate the molecular-level interaction of ILs and the consequent effect on proteins’ secondary/tertiary structure and biological activity. In their first report, showing the [C_8_mim]Cl interaction with L-tryptophan in water,^351^ the authors demonstrated that [C_8_mim]Cl quenches Trp fluorescence by a static mechanism, wherein the IL is weakly associated with Trp via van der Waals interactions and hydrogen bonding. This report had implications on the understanding of the molecular-level interactions of the IL-Trp system, which is generally used as an informative tool to monitor protein unfolding upon interaction with ILs. Following this, the authors reported static quenching of papain’s fluorescence with the decrease in activity by [C_8_mim]Cl and [C_4_mim]Cl due to H-bonding and van der Waals interactions.^352,353^ In a subsequent work,^354^ they reported quenching of the Trp fluorescence of papain or pepsin by [NH_2_C_2_C_4_im]Br based on static or dynamic mechanisms, either by H-bonding and van der Waals or hydrophobic interactions with the IL, respectively. They also showed that [NH_2_C_2_C_4_im]Br had no obvious effect on the secondary structure of both enzymes, leading to a slight increase in their activities due to the chaotropicity and hydrogen bond donating ability of [NH_2_C_2_C_4_im]^+^.^354^ Later, the authors reported the ILs induced activity decrease of lipase due to hydrophobic interactions of the cation and H-bonding interactions of the IL anions and the active site of the enzyme.^355^ They classified the cations effect in three groups based on decreasing activity, as follows: (I) [C_4_mim]Cl < [Bzmim]Cl < [C_7_mim]Cl < [C_8_mim]Cl; (II) [C_4_mim]Br < [Bzmim]Br < [C_7_mim]Br < [C_8_mim]Br; (III) [C_4_Py]Br < [C_8_Py]Br. The order of anions was represented as [C_4_mim]CF_3_SO_3_] > [C_4_mim]N(CN)_2_ ≈ [C_4_mim][ClO_4_] ≈ [C_4_mim]Br > [C_4_mim]Cl > [C_4_mim][BF_4_] and [C_4_mim][TfO] > [C_4_mim][N(CN)_2_] ≈ [C_4_mim][ClO_4_] ≈ [C_4_mim]Br.^355^ To generalize this notion the authors also investigated the effect of 12 ILs on the structure and activity of trypsin from a toxicity point of view.^356^ The order of ILs for trypsin inhibition was presented as [C_10_mim]Br > [C_8_mim]Br ≈ [C_6_mim]Br > [C_4_mim]Br; [C_10_mim]Cl > [C_8_mim]Cl ≈ [C_6_mim]Cl > [C_4_mim]Cl; and [C_4_mim]Br ≈ [C_4_mim][NO_3_] = [C_4_mim]Cl ≈ [C_4_mim][BF_4_] ≈ [C_4_mim][TfMs] > [C_4_mim][OAc].

**Figure 22:**
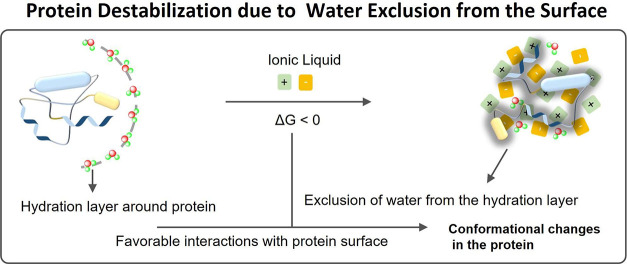
Mechanism of IL interaction with protein surface via favorable interactions.

From the thermodynamic analysis, H-bonding supported by hydrophobic interactions was given as the key driving force for structure and consequent activity inhibition of trypsin. Based on these studies, Fan et al.^355,356^ prepared a regression-based model, which produced satisfactory results to describe the relationship between the inhibitory ability and hydrophobicity or H-bonding ability of ILs toward proteins. This model could be useful and decrease the analysis time when investigating the interactions of ILs with proteins. Ventura et al.^357^ reported that ILs with higher β and π values are more effective in decreasing the activity of *Candida Antarctica* lipase B due to direct H-bonding and dispersion interactions with proteins around the active site. Support to the various experimental molecular interaction results also came from molecular dynamics studies, wherein Klähn et al.^73^ studied thermally induced unfolding of *Candida antarctica* lipase B (CAL-B) in the presence of a series of ILs, namely, [C_4_mim][NO_3_], [C_4_mim][BF_4_], [C_4_mim][PF_6_], [CH_3_OC_2_mim][BF_4_], acyclic [BAGUA][BF_4_], cyclic [BCGUA][BF_4_], cyclic nitrate [MCGUA][NO_3_], and cyclic [DCGUA][NO_3_]. They found that the destabilization of the protein surface was mostly facilitated by Coulombic interactions established between the IL anion that exhibits localized charge and strong polarization with the cationic residues, whereas the destabilization of the protein core was facilitated by direct hydrophobic interactions with core and alkyl chains of ILs, further inducing major conformational changes that enabled the access of ILs to the protein core. The surface instability resulted in the unraveling of α-helices, and an increase of surface area and radius of gyration of proteins, whereas core instability resulted in the disintegration of β-sheets due to the diffusion of ions into CAL-B and hence increasing protein-IL van der Waals interactions. Jaeger et al.^358^ simulated the effect of [C_2_mim][OAc] and [C_2_mim][C_2_OSO_3_] on the structure and activity of 11 xylanase from *Trichoderma longibrachiatum*, concluding that the enzyme solvated in higher concentrations of ILs generally remains more stable with respect to its crystal structure than when solvated in water. On the other hand, the decrease in activity arises due to the strong binding of [C_2_mim]^+^ to the active site of the enzyme by competitive inhibition. The mechanism of the IL’s interaction with protein surface via favorable interactions is illustrated in Figure 22.

From the described studies, it is evident that in the case of protein destabilization by ILs, the process begins with Coulombic interactions with the protein surface, which opens the protein hydrophobic core to facilitate hydrophobic associations. Further unfolding, refolding, or compaction of proteins depends on the IL concentration and its effect on the microenvironment of the protein. For example, Mangialardo et al.^359^ reported that [C_2_NH_3_][NO_3_] induced significant conformational transition of lysozyme, from fibril state to natural state, observed by a decrease in β-interchain conformation. The [C_2_NH_3_][NO_3_] reversed change in the microenvironment around Phe, Tyr, and Trp residues of lysozyme occurred via fibrillation. Similarly, Sankaranarayanan et al.^360^ reported the β → helix → β sheet conformational transition of myoglobin induced by an amino acid anion-based IL, i.e., [C_1_mim][Phe], at different concentrations by altering the polarity around the protein. Sankaranarayanan et al.^361^ also studied the effect of pH on nonspecific interactions of ILs to induce helix ↔ β conformation transition of bovine β-lactoglobulin at pH 4.0 and 7.5 in the presence of [C_2_mim][C_2_OSO_3_]. At pH 4.0, the protein initially changed to an intermediate β-turn structure, followed by a change to a more stable native β-sheet structure; at pH 7.5, the initial native β state traversed to a helical structure and then returned to the native β state due to the change in the microenvironment around the protein due to the IL concentration. Ghaedizadeh et al.^362^ reported a decrease in the activity of *Renilla* Luciferase due to [C_4_mim][BF_4_] and [C_4_mim][PF_6_] induced transition to a shrunken conformation and a coil-shaped structure due to collapse in α/β fold and reduction in α-helices. Rawat et al.^363^ reported dissociation of the aggregates of BSA, β-lactoglobulin, and immunoglobulin (IgG) into oligomers by ILs, namely, [C_2_mim]Cl, [C_4_mim]Cl, [C_6_mim]Cl, and [C_8_mim]Cl. The cations bind selectively to the negatively charged patches on the protein to induce dissociation, whereas the Cl^–^ anion stabilized oligomers via hydration effects. Bisht et al.^364^ reported the complete refolding of unfolded lysozyme by [C_4_(C_1_)_3_N][NTf_2_] and [C_1_(C_2_)_2_C_1_OC_2_N][NTf_2_], with a 13% increase in its functional activity by altering the solution properties.

Besides giving valuable information on the molecular mechanism of IL-protein interactions, the electrolytic nature of ILs as adjuvants has also been exploited for applications in oxygen sensing and IL-PAGE for the separation of proteins.^365,366^ Ding et al.^365^ reported that a direct electrochemical response of myoglobin could be observed for oxygen reduction from basal plane graphite (BPG) when [OHC_2_mim][BF_4_] was used as a supporting electrolyte. The myoglobin adsorbs on the surface of BPG forming a stable monolayer. The biosensor developed could directly detect the concentration of oxygen in an aqueous solution with a detection limit of 2.3 × 10^–8^ M. Hasan et al.^366^ exploited pyridinium-based ILs, viz. [C_*n*_PyrBr] (*n* = 4, 8), as buffer additive for high-resolution separation of low and high molecular weight proteins, including catalase, transferrin, BSA, ovalbumin, and α-lactalbumin, in IL-polyacrylamide gel electrophoresis as an alternative to the SDS-PAGE technique.

### 3.4 Ionic Liquids as Surfactants

Apart from the above-mentioned applications, formulations comprising surface-active ionic liquids (SAILs) and proteins are an emerging area of IL-protein research. SAILs are ILs that behave as ionic surfactants.^145^ SAILs show aggregation behavior similar to ionic surfactants when dissolved in any medium (polar/nonpolar), but they have different adsorption characteristics and may display superior surface-active properties if properly designed.^145^ The properties sometimes mimic the ones induced by the addition of bulky organic cations/anions to conventional ionic surfactants.^367^ Na[DBS], a common laundry detergent, has been compared with the SAIL [C_8_mim][C_12_OSO_3_] for the stabilization of a laundry enzyme—cellulase.^120^ Comparing the adsorption isotherms, Na[DBS] reduces the surface tension at CAC (2.9 mmol L^–1^) to 32.6 mN·m^–1^ and forms micelles,^120^ whereas [C_8_mim][C_12_OSO_3_] reduces the surface tension at CAC (0.42 mmol L^–1^) to 26 mN·m^–1^ and forms vesicles.^121^

**Figure 23:**
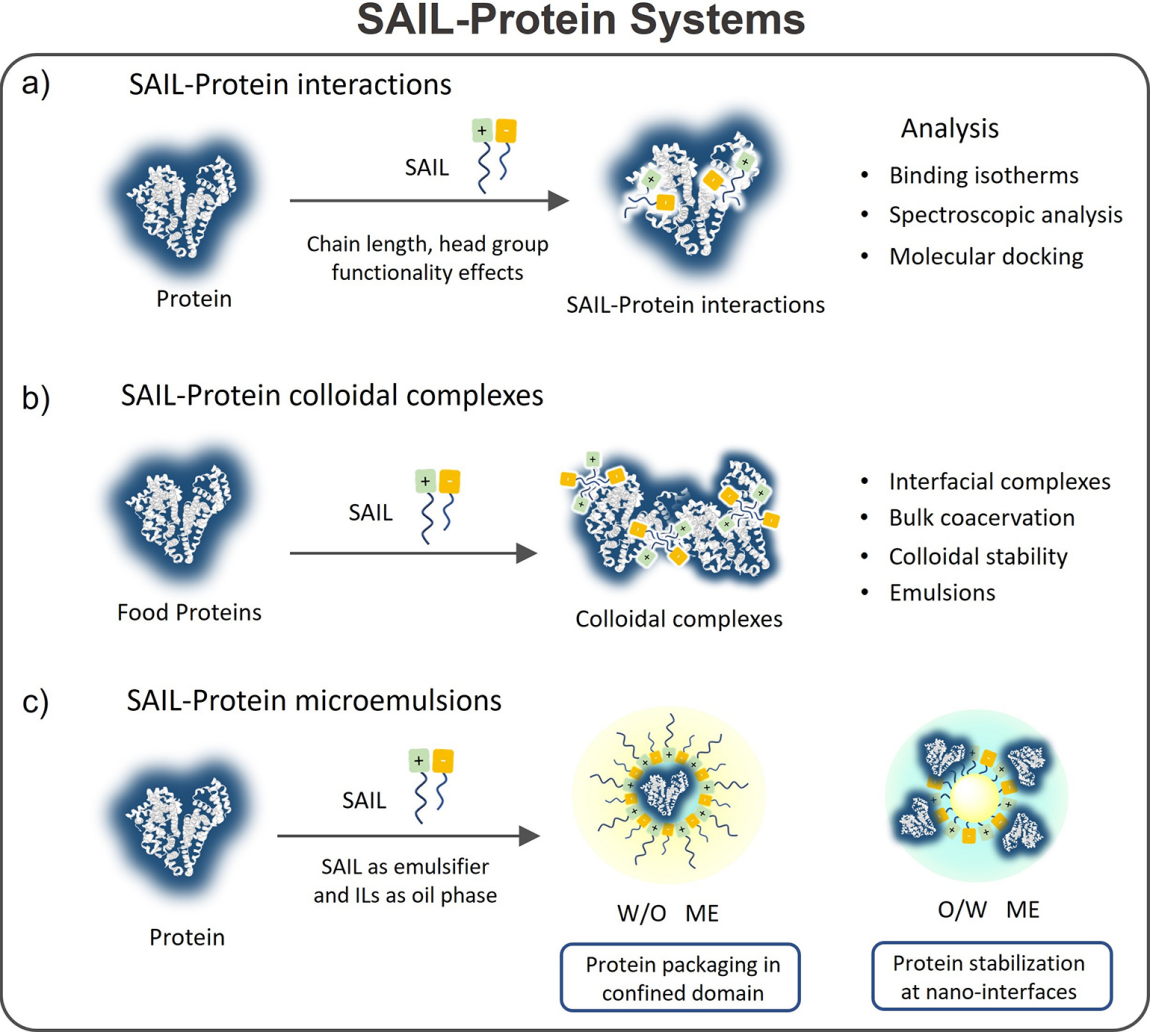
SAIL-protein systems: (a) SAIL-protein interaction studies; (b) SAIL-protein colloidal complexes; and (c) SAIL-protein microemulsions.

Therefore, [C_8_mim][C_12_OSO_3_] has better surface-active properties than Na[DBS] and is a promising candidate to replace it, if not bound by the constraints of biodegradation. The superior surface-active properties of SAILs could be exploited to formulate more efficient SAIL-protein systems for several applications.^143,144,223−226^ Most of the reported studies are focused on interfacial and spectroscopic analysis of the SAILs binding to proteins in different concentration regimes, and the consequent effect on their structure/function, coacervation, and storage in confined domains of microemulsions, as shown in Figure 23.

Geng et al.^116,117^ reported endothermic binding of [C_14_mim]Br to BSA, leading to (i) the protein secondary structure stabilization due to H-bonding below the CMC and (ii) destabilization due to hydrophobic interactions above the CMC. They concluded that compared to traditional cationic surfactants, [C_14_mim]Br is superior both in the protection and destabilization of BSA. Kumar and Kang’s research groups^118−123^ have been actively involved in understanding the effect of the SAILs chain length, headgroup functionality, and amphiphilicity on the structure and function of various proteins to materialize practically feasible formulations. For example, they have reported that by increasing the chain length of both the IL cation and anion, from [C_4_mim][C_8_OSO_3_]^118^ to [C_8_mim][C_12_OSO_3_],^119^ the stability of BSA is significantly enhanced.^118,119^ Increasing the chain length decreases the CAC from 26 mM to 0.39 mM, which reduces the denaturing monomeric binding regime of the SAIL to BSA and hence stabilizes it.^118,119^ They generalized this idea for colloidal stabilization of BSA in the cat-anionic vesicles of the ILs [C_8_mim]Br and the laundry detergent [Na][DBS] for 1 week of incubation, showing the relevance of IL-based molecular assemblies for kinetic stabilization of proteins.^120^ The authors further exploited the higher surface activity and protein stabilizing tendency of [C_8_mim][C_12_OSO_3_] to formulate a stable liquid colloidal formulation with a laundry enzyme cellulase as a candidate for detergent applications.^121^ For enhanced practical relevance, they synthesized a biodegradable SAIL, [Cho][DBS], and formulated a stable colloidal formulation with cellulase for potential liquid detergent application.^368^ In an interesting work,^122^ they further reported that the structure of the SAIL can be tuned to make assemblies that can mimic biological membranes. For this, they synthesized a SAIL, [Cho][AOT], having structural similarity to phosphatidylcholine, which is part of the inner membrane of mitochondria. The bilayers of [Cho][AOT] biomimicked the inner membrane of mitochondria in inducing all-α to α+β conformational transition of cytochrome c, supporting the relevance of this SAIL for studying *in vitro* membrane-protein interactions.^122^ Further, they altered the headgroup functionality of the imidazolium cation and reported ordered self-assemblies (long rods and helical fibers) of BSA in a specific concentration regime of amide, [C_12_Amim]Cl, and ester, [C_12_Emim]Cl, functionalized SAILs.^123^ Interestingly, fibers dissolved below and above this concentration regime, which gives an advantage to these SAILs, particularly for long-term stable kinetic packaging of BSA in the form of fibers.^123^ They also exploited colloidal complexes of the SAILs [C_12_C_1_im]Cl, [C_12_Amim]Cl, and [C_12_C_2_mim]Cl with BSA for pH-dependent controlled transport of a lipophilic dye—Rhodamine 6G (R_6_G).^369^

Several other research groups have also studied the effect of the cationic headgroup functionality on the structure of proteins. For example, Wang et al.^124^ reported that ester-functionalized SAILs, [C_1_COOC_2_C_1_im][C_12_OSO_3_] and [C_1_COOC_2_C_1_Py][C_12_OSO_3_], stabilized the secondary structure of BSA below 1 × 10^–3^ mol·L^–1^ and destabilized it above this concentration. The cationic imidazolium was found more destabilizing compared to the pyrrolidinium headgroup.^124^ Yan et al.^125^ reported that [C_10_mim]Br induced marked changes in the secondary structure of BSA, driven by strong hydrophobic interactions, compared to [C_*n*_mim]Br (*n* = 4, 6, 8).^125^ Pinto et al.^126^ reported that the pharmaceutical SAILs cetylpyridinium salicylate ([CetPy][Sal]) and benzethonium salicylate ([Be][Sal]) bind strongly to the hydrophobic sites of HSA and quench tryptophan fluorescence.^126^ Zhou et al.^127^ reported higher unfolding of BSA by imidazolium-based gemini surfactants ([C_*n*_-*s*-C_*n*_im]Br_2_, *n* = 10, 12, 14, *s* = 2, 4, 6) compared to quaternary ammonium surfactants (C_12_C_2_C_12_) and their corresponding monomers ([C_12_mim]Br and DTAB). This effect is due to stronger π–π interactions between the imidazolium rings on gemini surfactants with aromatic residues (Trp, Tyr, and Phe) of BSA, in addition to the electrostatic and hydrophobic interactions. Gospodarczyk et al.^128^ reported the stabilization of BSA by dicationic imidazolium (Gemini) surfactants, namely, [3, 3′[1,8(2,7-dioxooctane)]bis(1-dodecylimidazolium)chloride ([C_12_-oxyC_4_–C_12_im]Cl_2_) and [3, 3′[1,12(2,11-dioxadodecane)]bis(1-dodecylimidazolium)chloride ([C_12_-oxyC_8_–C_12_im]Cl_2_), at low concentration, followed by partial unfolding at higher concentration with the retrieval of unfolded structure post-CMC.^128^ Maurya et al.^129^ reported quenching of HSA fluorescence and a decrease in α-helical content, from 62.97% to 26.61%, in the concentration window of 0.99 × 10^–5^ to 13.0 × 10^–5^ M of a cationic imidazolium-based Gemini surfactant [C_12_-4-C_12_im]Br_2_ due to hydrophobic interactions.^129^ The IL [C_12_-4-C_12_im]Br_2_ was reported to act similarly toward lysozyme by quenching the fluorescence and decreasing the α-helix (from 34% to 29%) and β-sheet (from 28% to 9%).^130^

**Figure 24:**
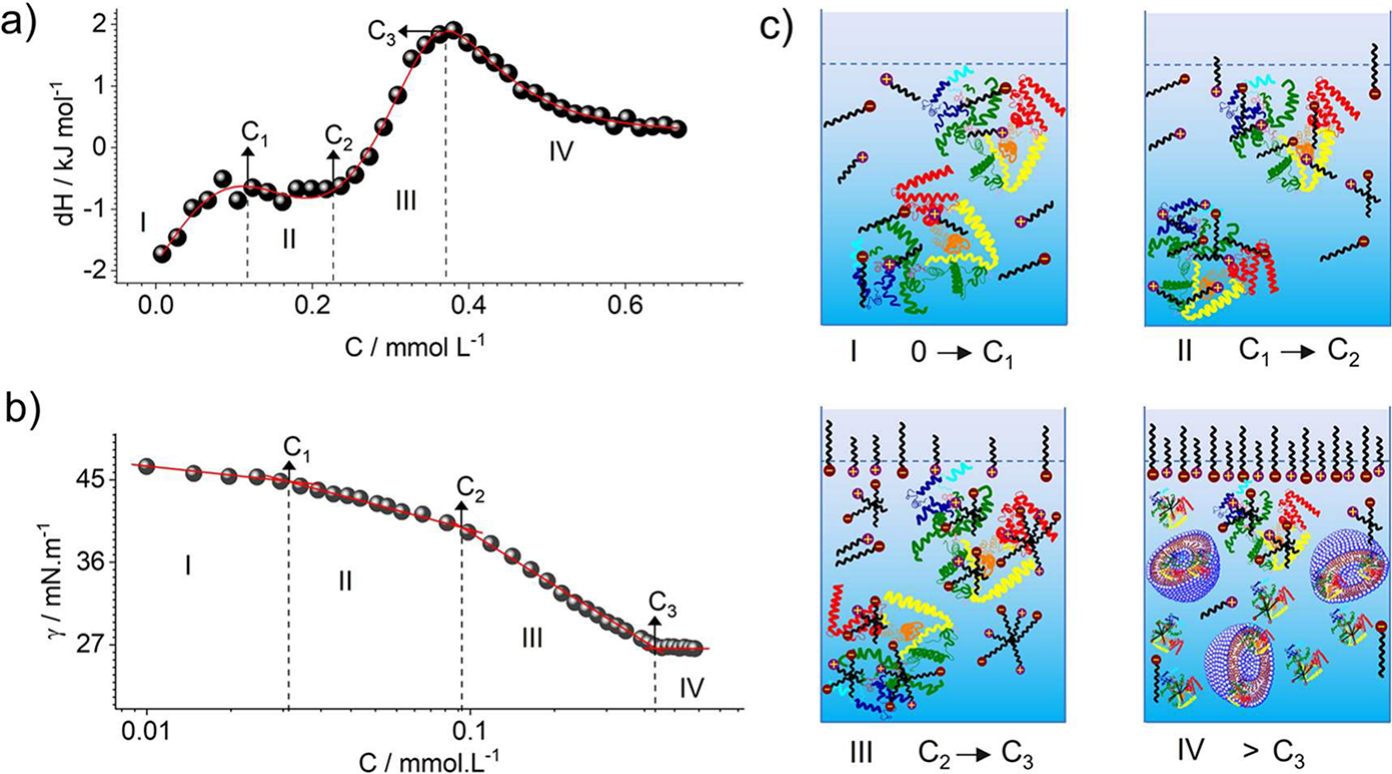
SAIL-protein interactions in aqueous solution at various concentration regimes, specified as I (0 → C_1_), II (C_1_ → C_2_), III (C_2_ → C_3_), and IV (>C_3_). These regimes can be identified with various techniques such as (a) ITC curves indicating various interaction regimes and (b) surface tension vs concentration plots indicating various interaction regimes. (c) Graphical illustration of SAIL-protein structures in various interaction regimes. The terms are C_1_, critical aggregation concentration; C_2_, protein saturation concentration; and C_3_, critical micelle/vesicular concentration.

Comparing the SAIL-protein interaction mechanism with ionic surfactant-protein systems, the mechanism is not very dissimilar.^224^ The overall analysis of the mechanism of interaction requires several techniques.^223,224^ Two key techniques to identify these interaction regimes are isothermal titration calorimetry (ITC) and tensiometry (Figure 24a,b).

In ITC, the binding curve of SAIL to protein can be obtained by subtracting the enthalpogram of the SAIL titration to the buffer solution from the SAIL titration to the protein solution (Figure 24a). Additionally, the adsorption isotherm of SAIL in protein solution is generally different from the one from the buffer solution until C_2_. This is because proteins, being differentially charged macromolecules, also possess inherent surface-active properties. Due to this they also adsorb at the air–liquid interface, thus leading to lower surface tension as compared to that of pure water. When SAIL is added to the protein solution it interacts with proteins both at the air–liquid interface and in bulk solution, leading to the formation of different SAIL-protein monomers and SAIL-protein aggregate complexes until C_2_. However, when the protein gets saturated with SAIL, further addition of SAIL displaces the SAIL-protein complexes from the air–liquid interface into the bulk and starts adsorbing at the interface until the formation of free micelles or vesicles in the solution (Figure 24c). That is why the adsorption isotherm of SAIL in pure water and SAIL in protein solution beyond C_2_ match exactly (Figure 24b). The feasibility of SAIL-protein interaction can be calculated using the following equation:^120,224^
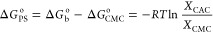
1where Δ*G*_PS_^o^ is the standard free energy of protein-surfactant interaction; Δ*G*_b_^o^ is the standard free energy of surfactant aggregation on polymer; Δ*G*_CMC_^o^ is the standard free energy of aggregation of surfactant in the protein solution; *R* is the universal gas constant; *T* is the temperature in Kelvin; and *X*_CAC_ and *X*_CMC_ are the mole fractions of SAIL at the CAC and CMC in the protein solution, respectively. The number of surfactant molecules binding per protein can be calculated from the slope of protein concentration vs the surfactant CMC in protein solution using the following equation:^120^
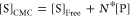
2where [S]_CMC_ is the surfactant concentration at CMC, [S]_Free_ is the free surfactant concentration, [P] is the concentration of protein under study, and *N* is the number of surfactant molecules attached to the protein.

These studies are important when considering interfacial and bulk complexation of SAILs with proteins to gain insights into their potential applications in food products. For example, Singh et al.^131^ reported interfacial complexation of [C_4_mim][C_8_OSO_3_] and [C_8_mim]Cl with a food protein—gelatin—at low concentration, whereas coacervation in the bulk solution was observed near the CMC. Interestingly, the [C_8_mim]Cl-gelatin coacervates remained stable up to a very high concentration beyond the IL CMC. Liu et al.^132^ investigated the detailed mechanism of the interaction of [C_12_mim]Br with the milk protein β-casein micelles (β-CM) in different concentration regimes. Below C_1_, the individual [C_12_mim]Br monomers bind to the β-CM shell close to the hydrophobic core to form a β-CM-[C_12_mim]Br (monomer) complex, further leading to a decrease in the environmental polarity of β-CM. Just over C_1_, [C_12_mim]Br molecules aggregate into micelle-like aggregates on the micellar shell, which led to the collapse of the N-terminal of β-casein and strengthened the hydrophobicity of the protein molecules, resulting in a more compact structure of β-CM. With a continuous increase in [C_12_mim]Br concentration, β-CMs are associated with each other in a network-like structure. Beyond C_3_, the net positive charges on the complexes, owing to the binding of more cationic surfactant molecules, lead to redissociation of the complexes, corresponding to the formation of the new nanosized β-CM-[C_12_mim]Br complexes. All the β-casein molecules are saturated by SAIL aggregates above C_s_, and free SAIL micelle-like aggregates appear in the bulk phase above the CMC.^132,133^

Cao et al.^134^ investigated the interfacial behavior of the protein β-casein in the presence of [C_16_mim]Br and found that at high concentrations of surfactant the [C_16_mim]Br and β-casein coadsorb at the air/water interface. However, when compared with β-casein/DTAB mixture at the air/water interface, the β-casein/[C_16_mim]Br solutions have a lower interfacial activity, which results from the stronger attraction with aromatic rings through π–π interaction. Cao et al.^135^ also studied the effect of [C_16_mim]Br on the interfacial properties of various forms (normal N form as well as the fast F and aged A forms) of BSA at the decane/water interface. The addition of [C_16_mim]Br did not influence the structure of BSA below the isoelectric point; however, a significant influence on the dynamic interfacial properties of BSA was observed above the isoelectric point due to the electrostatic interactions established between BSA and [C_16_mim]Br.^135^ Haung et al.^370^ investigated the interfacial and bulk properties of pepsin (PEP) in the presence of [C_16_mim]Br, wherein the globular structure of pepsin was proposed to be the decisive factor controlling the nature of the interfacial film. The authors reported negligible change in the conformation of pepsin both at the interface and in the bulk phase at low IL concentrations (1 × 10^–8^ to 1 × 10^–6^ mol L^–1^), whereas preferential unfolding of pepsin was observed primarily at the decane-water interface at moderate IL concentrations (1 × 10^–6^ to 5 × 10^–5^ mol L^–1^).^370^ Mandal et al.^136^ reported the formation of lysozyme-[C_4_mim][C_8_OSO_3_] coacervates between C_1_ and C_2_ controlled by protein concentration, secondary structural alterations, and solution pH. Singh et al.^371^ reported enhanced antimicrobial activity of lysozyme upon complexation with SAILs in water, namely, [Cho][Sar] and [Cho][Doc]. While these works have given useful insights about SAIL-protein coacervation and have shown promise in terms of enhanced emulsification, due to the toxicity of some imidazolium-based ILs, reservations persist for their practical applications in food industries.

Beyond interactions and coacervation studies, SAILs have been also used to stabilize the protein in confined domains of microemulsions for kinetic packaging and interfacial catalysis.^137−140,372−375^ Debnath et al.^372^ reported preservation of the secondary structure of trypsin with 4-fold higher activity in oil in water (o/w) microemulsion of [C_2_mim]Br confined in the water pool, using CTAB as an emulsifier. Pavlidis et al.^138^ reported a 4.4-fold rise in the activity and a 25-fold increase in the shelf life of lipase due to the adoption of a more rigid form of the protein in the confined domain of the water in IL ([C_4_mim][PF_6_] and [C_4_mim][BF_4_]) microemulsion, emulsified with Tween 80.^138^ Kundu et al.^373^ reported the stabilization of BSA in the confined water domain of water in oil microemulsion comprising a SAIL, i.e., [ProC_3_][LS], as the emulsifier and cyclohexane as the dispersion medium.^373^ Mao et al.^140^ selectively isolated hemoglobin at pH 5.0 in a water in oil microemulsion comprising [C_10_mim]Br as the emulsifier and [C_4_mim][PF_6_] as the dispersion medium. The isolated hemoglobin could be back-extracted with 55.6% efficiency in Britton-Robinson buffer at pH 12.0. Kaur et al.^374^ reported a high thermal stability of lysozyme, up to 120 °C, at the nanointerfaces of ethylene glycol in the [C_2_mim][NTf_2_] microemulsion with a dialkylimidazolium-based SAIL as emulsifier.^374^

Few other works have reported positive results on the encapsulation of proteins in SAILs micelles, their fibril inhibition, and refolding ability. For example, Bento et al.^375^ reported enhanced degradation of the dye indigo carmine B by laccase encapsulated in aqueous micelles of [N_10111_]Br and [C_10_mim]Cl. Alves et al.^376^ reported the stable encapsulation of lysozyme in micelles of a fluorinated anion-based SAIL, [C_1_mim][C_4_F_9_SO_3_], for protein drug delivery. Kundu et al.^377^ demonstrated the chain length effect in decreasing order of BSA fibril inhibition tendency of SAILs, as follows: [C_16_mim]Cl > [C_12_mim]Cl > [C_8_mim]Cl. Singh et al.^378^ reported higher refolding and stabilization of molten globule state of urea/GndHCl and alkali denatured Cyt c by [C_10_mim]Cl compared to [C_8_mim]Cl.^378^

#### 3.4.1 IL Surfactant-Protein vs Ionic Surfactant-Protein Systems

**Figure 25:**
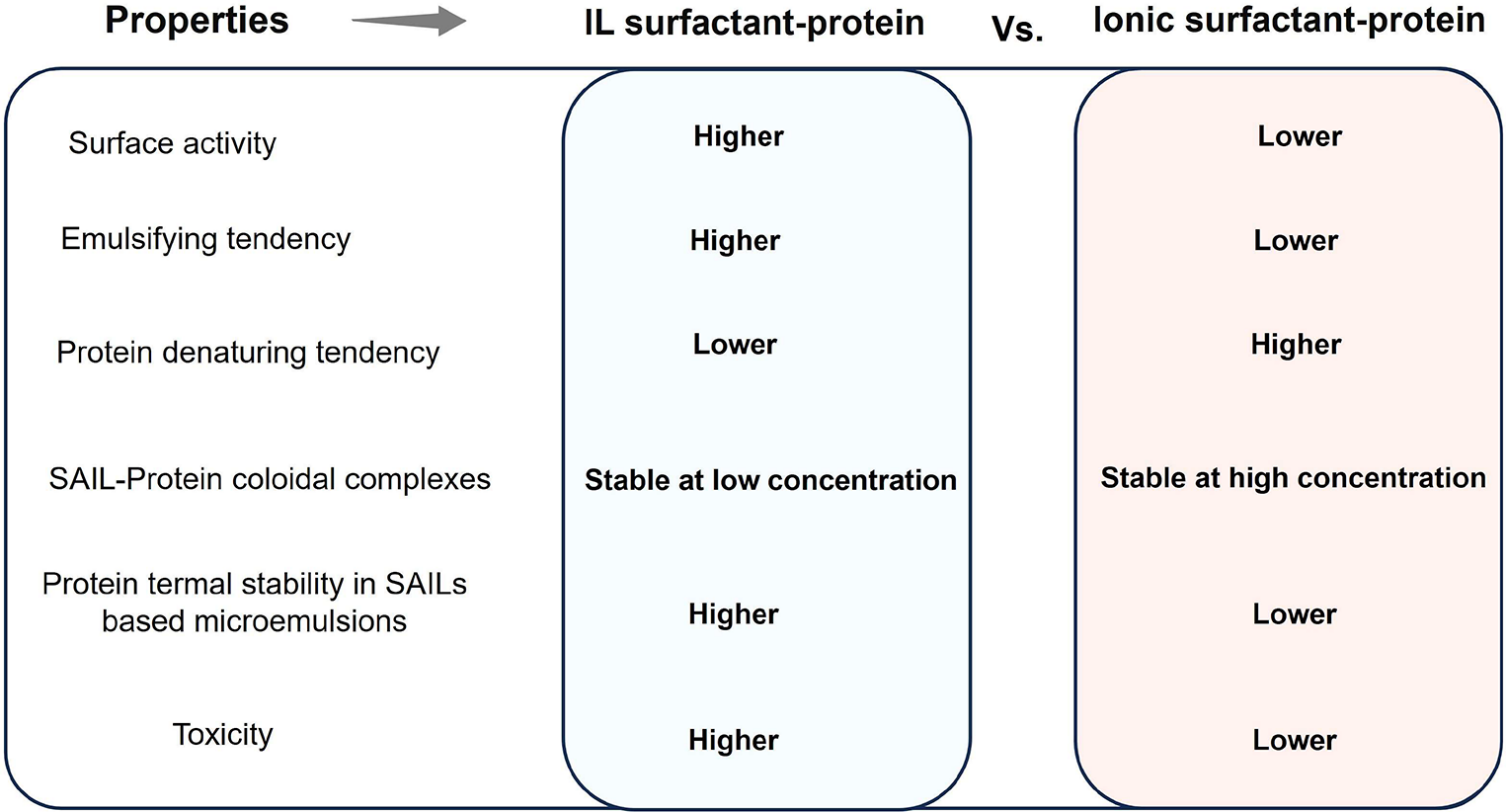
Comparative analysis of IL surfactant-protein vs ionic surfactant-protein systems.

The comparative analysis of IL surfactant-protein vs ionic surfactant-protein systems is depicted in Figure 25. The available SAIL-protein systems, although few to date, are superior in most cases when compared to the conventional surfactant-protein systems. For example, the SAILs are generally less denaturing toward proteins when compared to the conventional ionic surfactants.^118−130^ This behavior is due to the presence of a large organic cation in the IL which counters the direct interactions of the long-chain anion with proteins. Furthermore, ILs increase the aggregation tendency at low concentrations and decrease the denaturing monomeric binding region with the protein.^118−130^

Due to the low interfacial tension of SAIL-protein complexes, and protein stabilization in the monomeric regime, these systems have practical implications for practical *in vitro* stabilization of TPs during transportation. Moreover, they form stable colloidal complexes with food-grade proteins, like gelatin^131^ and β-casein^132−134^ at much lower concentrations; however, the possible toxicity of imidazolium-based SAILs used in such studies preclude their applications in food industries in the current scenario. The high emulsifying tendency of SAILs has been exploited to isolate, store, and enhance the activity of proteins in the confined domains or nanointerfaces by formulating them into microemulsions.^140,372−374^ Bharmoria et al.^149^ reported the thermal stabilization of cytochrome c in vesicular assemblies of the SAIL [Cho][AOT] in [C_2_mim][C_2_OSO_3_], up to 180 °C. Such assemblies could be useful in the stable packaging of TPs and other proteins translated to confined domains of microemulsions. Considering the stability of proteins observed in the quaternary ammonium family of ILs,^86,110^ these proteins can be confined in the polar phase of IL microemulsions for long-term packaging along with TPs. For example, Kaur et al.^374^ showed the stabilization of lysozyme at 120 °C in the confined nanointerface of the microemulsion containing ethylene glycol as the confined polar phase, dialkylimidazolium-based SAIL as an emulsifier, and [C_2_mim][Tf_2_N] as the nonpolar phase. These results lead to future possibilities for long-term functional packaging of proteins in all IL-based solvent media. SAILs can be a potential competitor to ionic surfactants in detergent industries due to their superior surface activity; however, efforts are needed to address their toxicity issues in addition to the stability of laundry enzymes (lipase, protease, and cellulases) in the formulation.

### 3.5 Proteins in Ionic-Liquid-Based Aqueous Biphasic Systems

The processing/extraction of pure proteins from aqueous media involves, most of the time, the use of highly viscous hydrophobic (nonwater-soluble) ILs with a low protein-friendly character. To overcome these concerns, hydrophilic ILs have been investigated in liquid–liquid extraction in the form of aqueous biphasic systems (ABSs). ABSs are ternary systems composed of water and two other phase-forming components, in which above a certain concentration occurs phase separation. Each phase is aqueous and enriched in one of the other remaining components. Conventional ABSs are formed by polymer–polymer, polymer–salt, or salt–salt combinations.^379^ In 2003, Rogers and co-workers^380^ demonstrated the potential of ILs to prepare ABSs by mixing them with inorganic salts. Thereafter, several studies available in the literature clarified that IL-based ABSs can be generated with a wide variety of organic/inorganic salts, polymers, amino acids, and carbohydrates, which have been reviewed in detail, including applications with proteins, in our earlier reviews.^17,381^

**Figure 26:**
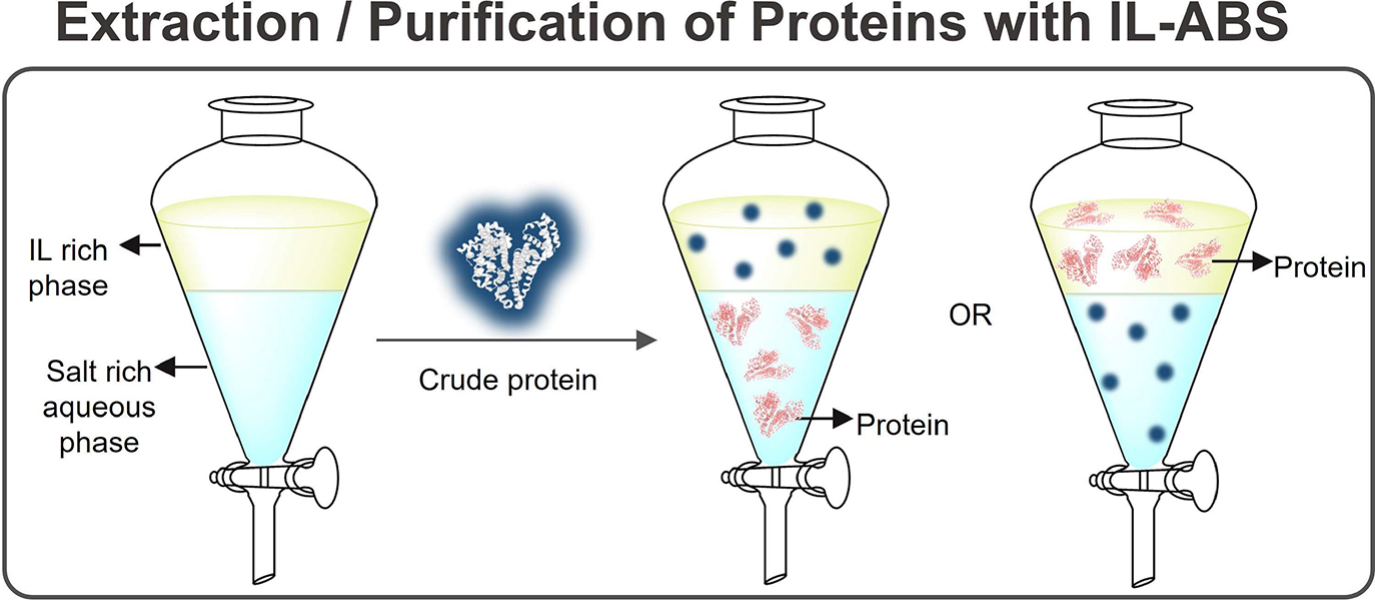
Extraction and purification of proteins using IL-based ABSs.

More recently a large number of works published to date have focused on the extraction performance of ABSs for proteins, with few addressing however the protein’s stability and mechanisms of interaction with ILs. Although we could think on the IL co-solvent effect, as discussed before, ABSs are more intricate since a third component is also present at the IL-rich phase. An illustration of steps carried out for the extraction and purification of proteins using IL-ABSs is shown in Figure 26.

Due to their natural affinity for water, proteins are expected to partition into the aqueous phase of IL-ABS, but both phases are water-rich. Therefore, there is not a universal phenomenon dictating protein partitioning; still, in ABSs comprising ILs most proteins tend to partition to the IL-rich phase, a phenomenon that is independent of having or not a strong salting-out species as the third phase-forming component. For example, Pereira et al.^378^ reported the single-step extraction of BSA into the IL-rich phase of ABS composed of phosphonium- and quaternary ammonium-based ILs combined with potassium citrate/citric acid at pH 7.0. The authors used different ILs ([P_4444_]Br, [P_4444_]Cl, [P_i_(_444)_1][Tos], [P_4441_][C_1_OSO_3_], and [N_4444_]Cl) for the separation of BSA. SE-HPLC and FT-IR studies confirmed the stability of BSA extracted into the IL-rich phase.

Furthermore, BSA was recovered from the IL-rich phase via dialysis, followed by the IL reuse. On the other hand, Quental et al.^157^ reported 100% extraction efficiency of a stable BSA from the fetal bovine serum in the IL-rich phase of ABSs formed by cholinium-based ILs ([Cho][OAc], [Cho][Bit], and [Cho][Prop]) and polypropylene glycol with a molecular weight of 400 g·mol^–1^ (PPG 400). Both works^382,157^ show that proteins prefer the IL-rich phase, independent of having a second phase enriched in a salt or in a polymer. Therefore, protein’s portioning in IL-based ABSs is majorly governed by specific interactions occurring with the ILs instead of being ruled by a salting-out phenomenon.

Taha et al.^383^ explored the use of ABSs comprising cholinium-based ILs with Good’s buffer anions for the extraction of BSA. The advantages of the studied ILs are their self-buffering properties in the biological pH range and their easily tunable polarity and hydrophobicity. Complete extraction of BSA was achieved into the IL-rich phase, without compromising the structural stability of BSA as confirmed from CD and FT-IR analysis. The authors also compared the stability of BSA in aqueous solutions of the ILs with the respective Good’s buffer precursors, a more conventional IL ([Cho]Cl), and a well-known protein stabilizer (sucrose). The α-helicity of BSA was shown to follow the order [Cho][TES] > [Cho][Tricine] > [Cho][HEPES] > sucrose > TES > [Cho][Cl] > HEPES > Tricine. These results show that the studied ILs are better stabilizers of BSA, which is due to the dual hydrogen bonding established between the IL ions and BSA as revealed from molecular docking studies. Gupta et al.^384^ also reported 100% extraction efficiency of α-chymotrypsin using IL-ABSs comprising Good’s buffer ILs. They concluded that the studied ILs provide a better stabilizing effect toward α-chymotrypsin, which was confirmed from tryptophan fluorescence studies. The same type of ILs was used for the extraction of TPs, namely, immunoglobulin Y (IgY) from egg yolk, with extraction efficiencies ranging from 79 to 94%.^385^ Ramalho et al.^386^ reported the single-step extraction of IgG from rabbit serum using IL-ABS, as confirmed by SE-HPLC.^386^ Mondal et al.^387^ also reported the single-step extraction of IgG from rabbit serum into the IL-rich phase using ABSs formed by cholinium-based ILs, with 85% extraction efficiency. The structural integrity of the extracted protein was confirmed by SE-HPLC, SDS-PAGE, and FT-IR. The purity of the extracted IgG was enhanced by 58% when compared with its purity in serum samples.^387^

**Figure 27:**
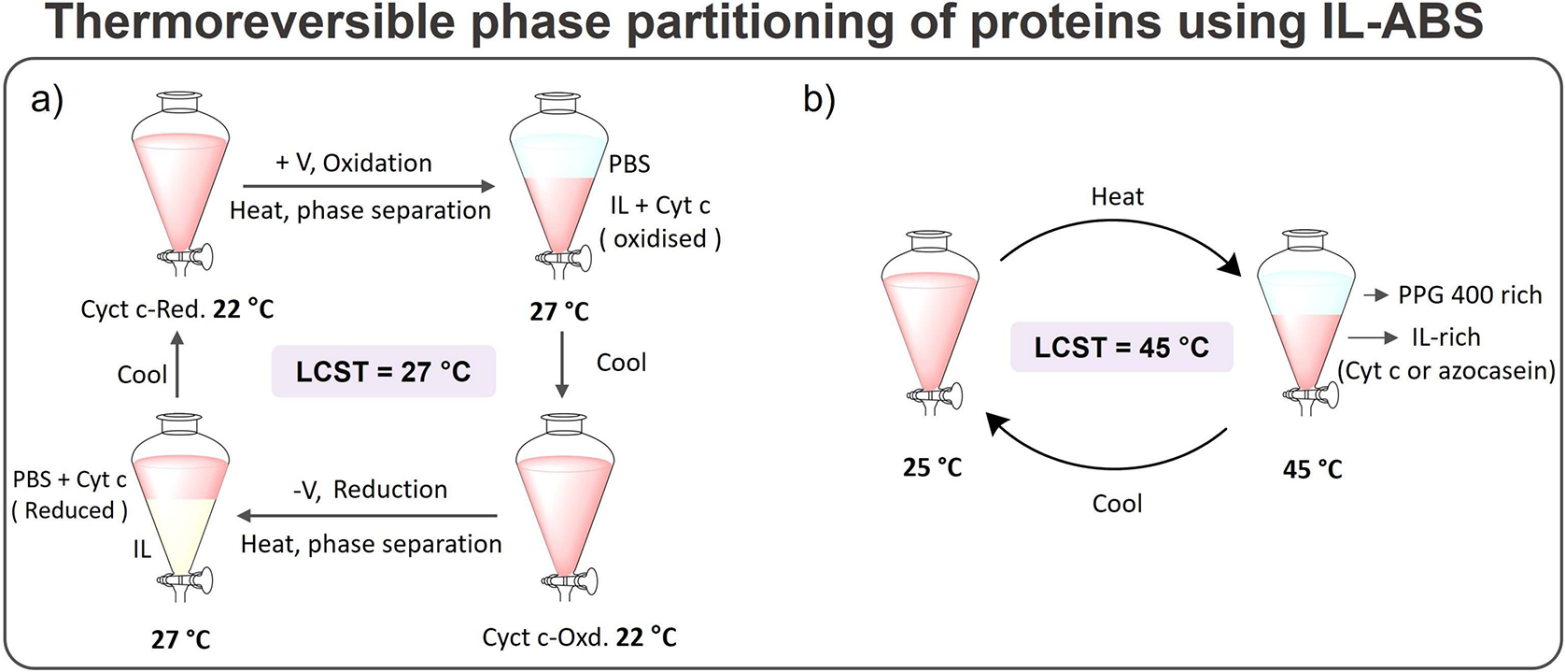
Illustration of the use of thermo-reversible IL-based systems for the separation of proteins: (a) 50% [P_4,4,4,4_][TMBS] + 50% H_2_O and (b) 6% [N_11[2(N11)]0_][C_1_CO_2_]+ 30 PPG 400 + 64% H_2_O.

Beyond molecular interactions and salting-out effects, other factors like molecular recognition and temperature have been reported to affect the stability of proteins in IL-ABSs. For example, Tseng et al.^388^ explored the importance of biomolecular recognition for the extraction of arginine and lysine-based peptides and proteins from the aqueous phase to crowned 1,2,3-triazolium cation and [NTf_2_]-based IL phase of IL-ABS. Both peptides and proteins retained their structural stability upon extraction into the IL-rich phase, as confirmed from NMR and CD analysis.^388^ Ikeda et al.^389^ investigated the effect of the lower critical solution temperature of the IL [P_4,4,4,4_][TMBS] for the partitioning of Cyt c. The authors found the selective partitioning of the oxidized and reduced form of Cytochrome c into the IL or buffer phase. The oxidized cytochrome c gets transferred to the [P_4,4,4,4_][TMBS] phase, whereas reduced Cyt c remained in the buffer phase in the functionally active forms (Figure 27a).

Passos et al.^158^ also investigated the low critical solution temperature behavior of IL-ABS comprising protic ILs and PPG 400 for the separation of proteins, namely, Cytochrome c and azocasein. Both proteins maintained their native structure in the IL-rich phase at 45 °C, as confirmed from FT-IR results (Figure 27b). The described temperature-sensitive IL-ABSs should be explored at the practical scale for the extraction and purification of high-value proteins, especially TPs such as immunoglobulins and other recombinant proteins. Additionally, challenges associated with the economic viability and the stable extraction with high purity from the real protein matrices must be resolved, aiming at establishing sustainable IL-ABSs for protein purification and stabilization.^390^

### 3.6 Poly(ionic liquid)-Protein Conjugates

**Figure 28:**
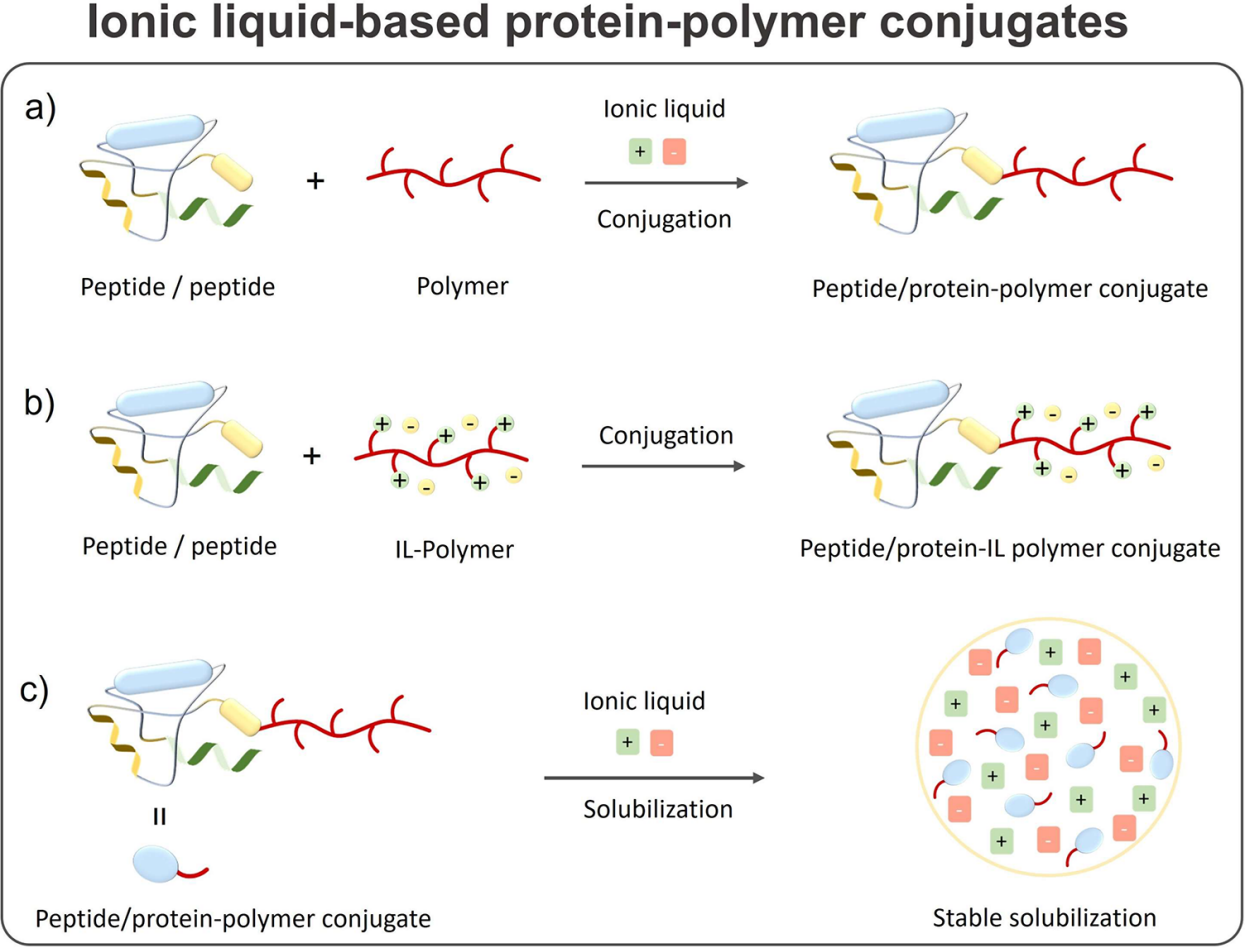
Strategies of IL-based protein–polymer conjugates: (a) ILs or A-ILCS as medium for stable protein–polymer conjugation; (b) ILs as medium for solubilization and stability of protein–polymer conjugates; and (3) stable protein-IL polymer conjugates.

PEGylation is extensively used to shield proteins from enzymatic degradation, improving their long-term storage and pharmacokinetic efficiency.^153^ However, sometimes the success of such protein–polymer conjugations is largely constrained, namely by (i) low protein conjugation efficiency; (ii) restricted choice of solvents, such as water, methanol, and DMSO; (iii) laborious and time-consuming polymerization, workup, and storage processes; (iv) denaturation of proteins during harsh conditions applied for polymer conjugation; and (v) competitive hydrolysis of polymer side-end chain having amide functionalities.^153^ Taking these issues altogether, ILs can be employed as potential alternatives for the synthesis of protein/peptide–polymer conjugates and as media for packaging proteins. Table 2 summarizes the reported works in both fields. These works can be divided into three categories: (1) ILs or A-ILCSs as medium for stable protein-polymer conjugation; (2) stable protein-IL polymer conjugates; and (3) ILs as medium for solubilization and thermal stability of protein-polymer conjugates. An illustration of these systems is shown in Figure 28.

**Table 2:** Summary of the IL-Based Protein/Peptide Polymer Conjugates

P–P Conjugate	Ionic Liquid as Solvent/Conjugate
Lysozyme-polyMPC^153^	[C_2_mim][TfO]
BM_2_^391^	[C_2_mim][OAc]
Imidazolium salt-peptide conjugates^392^	[(CO_2_H)^4^C_4_C_1_im]Br^–^ and [(CO_2_H)^15^C_15_C_1_im]Br^–^
Cytochrome P450-PEO^389^	[C_2_mim][Tf_2_N]
Lipase A-PAcMO^394^	[C_4_mim][PF_6_]
Myoglobin-glycolic acid ethoxylate lauryl ether^150^	[bmpy][TfO] and [bmpy][Tf_2_N]
[C-IgG][S] liquid ionic conjugate^395^	[C-IgG][S]

Chen et al.^153^ reported one-pot synthesis of lysozyme-poly(methacryloyloxyethyl phosphorylcholine) (polyMPC) conjugate in [C_2_mim][TfO] with sodium borate buffer at pH 9.0. The authors observed less than 5% of aggregation of the conjugate from SEC-HPLC analysis, thus demonstrating the significant stability of the prepared conjugate. Baumruck et al.^391^ reported the native chemical ligation of highly hydrophobic membrane-associated peptides in [C_2_mim][OAc]/buffer mixture at pH 7–7.5, with 80–95% yield, outperforming the existing gold standard ligation method. The secondary structural integrity of these peptides was confirmed from CD spectroscopy.^391^ Reinhardt et al.^392^ reported a structurally stable imidazolium salt-antimicrobial peptide conjugate showing enhanced antimicrobial activity compared to the nonconjugated peptide.^392^ Ohno and co-workers^393^ showed solubility and enhanced thermal stability up to 120 °C of a chemically modified Cytochrome P450 with poly(ethylene oxide) in [C_2_mim][Tf_2_N].^393^ Chado et al.^394^ reported 19-fold enhancement in the activity of covalent functionalized lipase A with polymer poly(4-acryloylmorpholine) (PAcMO) dissolved in [C_4_mim][PF_6_].^394^ Brogan et al.^150^ conjugated myoglobin with surfactant glycolic acid ethoxylate lauryl ether, leading to 84 surfactant molecules per protein. The PP-conjugate showed significant solubility in dry ILs, with the retention of the secondary structure up to 55 °C.^150^ Overall, this is an innovative approach to overcome the limitations of the robustness of proteins to get solubilized in dry ILs and should be implemented for thermal stabilization of other proteins and TPs. In this direction, Slocik et al.^395^ investigated the polycationic nature of modified antibodies and their ability to form ion pairs for the conversion of primary Immunoglobulin G antibodies into stable protein liquids that retained more than 60% binding activity after repeated heating up to 125 °C. The IgG cantonized with 1-ethyl-3-(3-dimethyl aminopropyl) carbodiimide (C-IgG) was paired with Poly(ethylene glycol) 4-nonyl phenyl 3-sulfopropyl ether anion (S) to form a room-temperature protein ionic liquid (P-IL).^395^ This is an important advancement on grounds of the poor temperature-dependent stability of therapeutic proteins for long-term storage and must be more deeply investigated. Using atomistic MD simulations, Balasubramanian et al.^396^ reported that the polymer surfactant functionalized lipase A, lysozyme, and myoglobin dissolved in [C_2_mim][NTf_2_] exhibits higher intraprotein hydrogen bonding, in addition to the greater thermal stability and IL tolerance due to the screening of protein-IL interactions.^396^

IL-based protein-polymer conjugates are mainly comprised of imidazolium-based ILs combined with chloride, bromide, acetate, Dicyandiamide, and tetrafluoroborate anions. Very few studies emphasized the potential of biocompatible and biodegradable cholinium-based ILs as prospective alternatives to imidazolium ones. Nowadays, there is pervasive availability of more benign ILs, such as those composed of cholinium-based cations combined with anions derived from carboxylic acids, biological buffers, among others, which should be explored for the synthesis of therapeutic protein-polymer conjugates and application in the biopharmaceutical field.

## 4 Conclusions and Future Prospects

Herein, we have comprehensively reviewed the works published to date on IL-protein systems, excluding however works dealing with biocatalysis. These systems comprise proteins dissolved in (*i*) neat ILs; (*ii*) ILs as co-solvents; (*iii*) ILs as adjuvants; (*iv*) ILs as surfactants; (*v*) ILs as phase-forming components of aqueous biphasic systems; and (*vi*) IL-polymer-protein/peptide conjugates. The reviewed works allowed us to make inferences regarding the main molecular mechanisms and IL-protein interactions affecting the stability/conformational alteration/unfolding/misfolding/refolding of proteins.

The expectancy of high-temperature biocatalysis has been the driving force in studies involving enzymes and NILs. However, few NILs have successfully cracked the hard nut of solubilizing proteins of different secondary structural conformations such as BSA, HSA, α-chymotrypsin, ovalbumin, myoglobin, lactoferrin, silk fibroin, zein, and keratin. The formation or disruption of hydrogen bonding between IL ions and proteins has been found to be the major force for the stable solubilization of the investigated proteins. Therefore, from a protein packaging perspective, ILs possessing H-bonding ability in both the cation and anion, such as [C_*n*_OHmim][HOC_*n*_SO_3_], have been identified as the most efficient. Few other ILs, such as [C_2_mim][C_2_OSO_3_] and [C_4_mim][OAc], have been found to solubilize proteins of all kinds of secondary structures. This possibility is due to their nanoheterogeneous structure and should be taken as standard when designing new NILs for protein solubilization. Although the published results to date are promising and optimistic, the practical feasibility of such systems for protein packaging is rather gloomy. This is due to various factors, namely, (*i*) lack of systematic research in one IL or protein direction (too many scattered ILs and proteins have been investigated), (*ii*) biocompatibility issues of promising ILs reported, and (*iii*) lack of processes for suitable re-extraction of proteins after solubilization in NILs.

On the co-solvent front, results reported for protein stabilization are more promising than in NILs. The paradigm of introducing water or an aqueous medium as the environment around proteins is indeed an advantage from several points of view. The stability of proteins has been explained by hydrophobic solvation, H-bonding, and preferential exclusion phenomena. The most promising IL-water systems identified are the ones containing quaternary ammonium cations, which have shown stabilization (thermal, structural, and functional) toward several proteins, operating via either preferential exclusion or H-bonding mechanisms. However, the stabilization effect in most cases depends on the concentration of IL and type of IL ions. Unlike with the fewer works available on NIL-protein systems, concerted efforts have been made by various research groups in A-ILCS-protein systems. A-ILCSs of the quaternary ammonium family have been particularly investigated for protein stability, refolding, and cryoprotection. [Cho][dhp] has been identified as the best candidate to stabilize Cytochrome c for long periods, providing evidence on the use of A-ILCS of [Cho][dhp] for protein packaging purposes. The [Cho][dhp] was also investigated for the stabilization of relevant TPs, namely, Interleukin 2 and IgG. The stabilization of other TPs, such as insulin, against thermal and aggregation-induced denaturation was demonstrated with the use of A-ILCS comprising [(C_2_)_3_NH][dhp]. Concerning imidazolium-based ILs, some of them have shown protein stabilization via hydrophobic solvation and clustering of the imidazolium cation at the protein surface, both via electrostatic and hydrophobic interactions. However, their precipitation effect on proteins came as blessings in disguise, followed by their use to develop strategies for the crystallization of proteins.

As far as IL adjuvants are concerned, valuable information regarding molecular-level interactions with proteins has been reported from both experimental and computational studies. Intrinsic fluorescence of BSA by Trp residue was used to locate the binding regions based on fluorescence quenching. However, the structural alterations in proteins were mainly documented from secondary structural behavior, assessed by circular dichroism and FT-IR. All kinds of noncovalent interactions (electrostatic, H-bonding, hydrophobic, and van der Waals) were used to support the stabilization/destabilization/conformational transition effects depending upon the cation and anions of ILs used. The Hofmeister effect based on preferential binding or exclusion phenomena has been applied to explain the IL-induced effect on proteins, however with large inconsistency and with rare consensus among different research groups. The understanding of the molecular mechanism of IL-protein interactions using spectroscopic techniques allowed the report of a regression-based model that produces satisfactory results to describe the relationship between the inhibitory ability, hydrophobicity, and H-bonding ability of ILs toward proteins. This type of model could be useful and can limit the analysis time when investigating the interactions of ILs with proteins. Moreover, the application of ILs as adjuvants to resolve bands in SDS-PAGE is another promising application reported, having relevant implications for biochemists and biotechnologists, and must be exercised with other ILs seeking further improvements in resolution.

In the case of surfactant ILs, the most important advantage over conventional ionic surfactants is that they are most of the time observed with higher surface activity, with reduced CAC. This behavior is useful for application in food industries and cleaning purposes. The mechanism of interactions is not very dissimilar to conventional surfactant-protein systems. On a positive note, their denaturing tendency toward proteins has been found to be lesser than their conventional counterparts. This behavior can be attributed to the presence of larger organic cations. Moreover, SAIL-protein combinations lead to lower interfacial tensions and stabilize the protein in a monomeric regime, which is important for TP stabilization and from a toxicity point of view. One of the key advantages that SAILs further exhibit is their thermal stability, which could be utilized to store proteins in assemblies like microemulsions. In this field, the works reporting the isolation of hemoglobin in the polar part of the water in IL microemulsion and thermal stabilization of cytochrome c in vesicular assemblies up to 180 °C hold high relevance. The key limitation of SAILs is that most of them belong to the imidazolium cation family that has been raising toxicity and biocompatibility issues.

As far as IL-ABS-protein and IL-protein-polymer conjugates are concerned, they are promising systems with wide implications. IL-ABSs are particularly useful on account of providing a water-rich medium during extraction or purification steps. However, the stability of the protein has been poorly addressed in IL-ABSs after the extraction step. Promising results in this direction are works on the use of IL-ABSs to purify high-value proteins, such as biopharmaceuticals, and the use of stimuli-responsive IL-ABSs that provide technical advantages. IL-protein-polymer conjugates have been scarcely investigated. Yet, promising results have been published on their use toward the stable packaging of proteins at room or high temperature. In the available works, proteins were functionalized with polymers to be either soluble in ILs or to behave as an IL by themselves.

**Figure 29:**
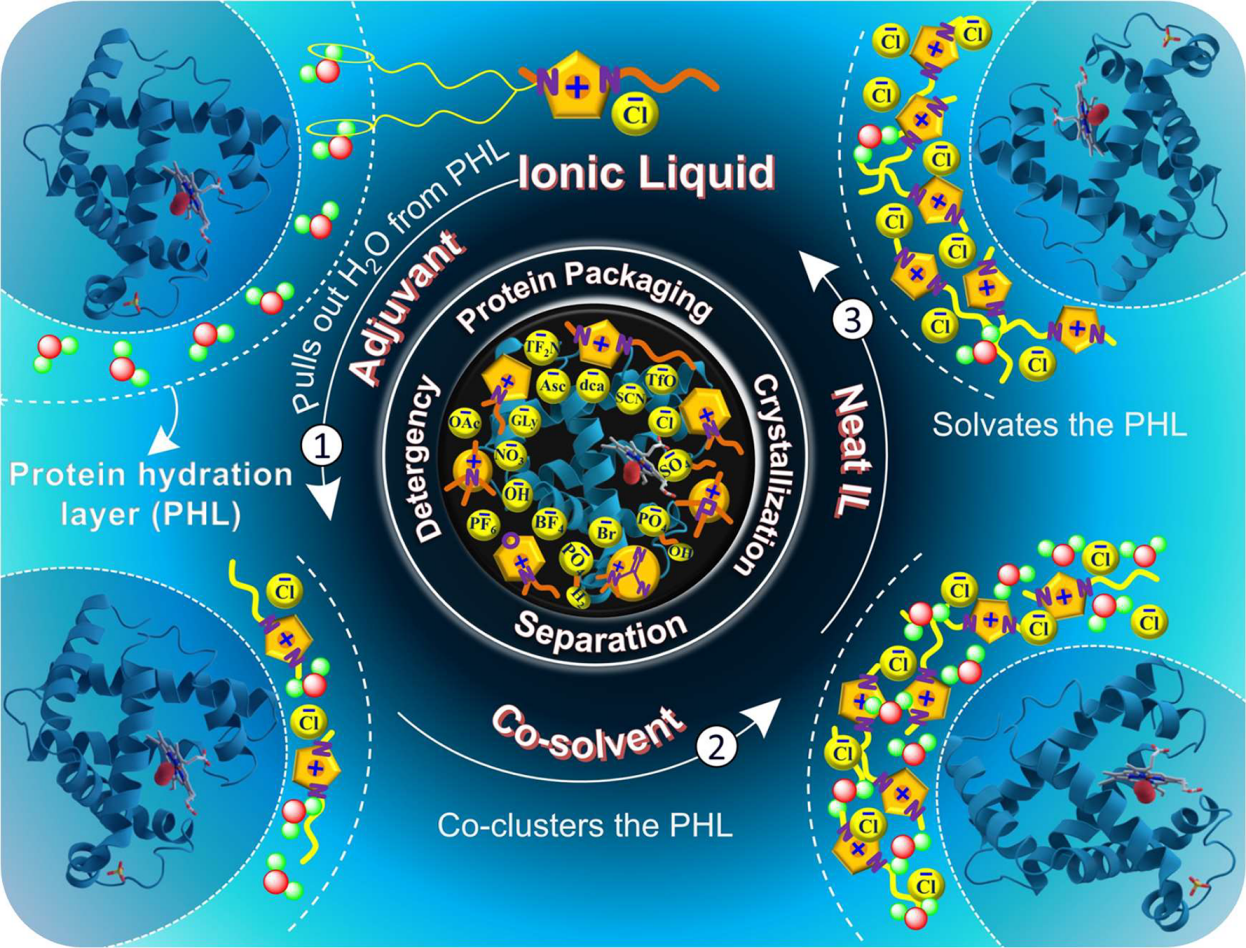
Summary of IL-protein interactions wherein (1) ILs as adjuvants in most cases pull out water from the protein surface due to favorable interactions with the protein surface; (2) ILs as co-solvents co-cluster in the protein hydration layer; and (3) ILs as native solvents solvate the protein.

Since the inception of the IL-protein journey, significant contributions have been made by several authors, on both the fundamental and applied fronts. The field has moved beyond enzyme biocatalysis, and relevant and high-value proteins, such as TPs, have been considered with ILs. All contributions have certainly shown the relevance of ILs in the field of proteins, but it is still in its infancy concerning mature and concrete technological applications. On the optimistic front, the promising contributions available do support the IL potential in the field of proteins, and yes, it can be stated that “Ionic liquids, if properly designed, do exhibit suitable characteristics to dissolve, extract, stabilize and purify proteins”. The IL-protein interactions with different forms of ILs and applications are summarized in Figure 29. The list of promising systems having both fundamental and applicative implications produced to date is summarized in Table 3.

**Table 3:** List of Most Promising Protein-IL Systems Using ILs in Various Forms

Protein	Ionic Liquids	Category	Goal
CAL B^29^	[Me(OEt)_3_-Et_3_N][OAc], [Me(OEt)_3_-Et-Im][OAc]	NIL	Stable Packaging
Cytochrome c^31,32^	[C_2_mim][C_2_OSO_3_], [Amim]Cl	NIL	Stable Packaging
CALB^275^	[C_2_OHmim][Tf_2_N], [C_3_OHmim][Tf_2_N] [C_3_OHTEA][HOC_2_SO_3_]	NIL	Stable packaging
Insulin^282^	[Cho][gerenate]	NIL	Stable packaging
Monellin^59^	[C_4_mpy][Tf_2_N]	A-ILCS	Stable packaging
Cytochrome^61,63,66,296^	[Cho][dhp]	A-ILCS	Stable packaging
Interleukin 2, IgG^299,300^	[Cho][dhp]	A-ILCS	Stable packaging
α-chymotrypsin^68,291^	[(C_2_H_5_)_3_NH][OAc], [(C_2_H_5_)_3_NH][PO_4_]	A-ILCS	Stable packaging
Insulin^301^	[(C_2_)_3_NH][dhp],	A-ILCS	Aggregation suppression
Lysozyme^312^	[CH_3_CH_2_NH_3_][NO_3_]	A-ILCS	Stable packaging
Lysozyme^333^	[C_4_mim]Cl	A-ILCS	Crystallization
Lysozyme^331^	[OHC_2_NH_3_][HCOO], [(C_1_)_2_OHC_2_NH][OHC_2_COO]	A-ILA	Crystallization
BSA, β-lactoglobulin (IgG)^363^	[C_2_mim]Cl, [C_4_mim]Cl, [C_6_mim]Cl, [C_8_mim]Cl	A-ILA	Protein fibril dissolution
BSA, ovalbumin, α-lactalbumin^366^	[C_4_Pyr]Br, [C_8_Pyr]Br, [C_11_Pyr]Br	A-ILA	Protein’s separation
Insulin^317^	[PAN][NO_3_]	A-ILA	Aggregation suppression
pepsin and papain^354^	[NH_2_C_2_C_4_im]Br	A-ILA	Activity enhancer
Trypsin^372^	[C_2_min]Br/CTAB	IL-ME	Activity enhancer
Lipase^138^	[C_4_mim][PF_6_], [C_4_mim][BF_4_]	IL-ME	Activity enhancer
Hemoglobin^140^	[C_10_mim][Br]+[C_4_mim][PF_6_]	IL-ME	Stable extraction
Cellulase^121,368^	[C_8_mim][C_12_OSO_3_], [Cho][DBS]	SAIL	Detergency
Cytochrome c^149^	[Cho][AOT]+[C_2_mim][C_2_OSO_3_]	SAIL	Stable packaging
BSA^123^	[C_12_Amim]Cl, [C_12_Emim]Cl	SAIL	Protein fibril dissolution
BSA^383^	[Cho][TES], [Cho][Tricine], [Cho][HEPES],	IL-ABS	Stable extraction
IgY^383^	[Cho][MES], [Cho][Tricine], [Cho][CHES], [Cho][HEPES], [Cho][TES]	IL-ABS	Stable extraction
Cytochrome c and Azocasein^158^	[N_11[2(N11)]0_][C_1_CO_2_]	IL-ABS	Stable extraction
IgG^387^	[Cho][Asc]	IL-ABS	Stable extraction
Myoglobin^150^	[bmpy][NTf_2_], [bmpy][OTf]	P–P–Conjugate	Stable Packaging
IgG^395^	[C-IgG][S]	P-IL	Stable Packaging

**Figure 30:**
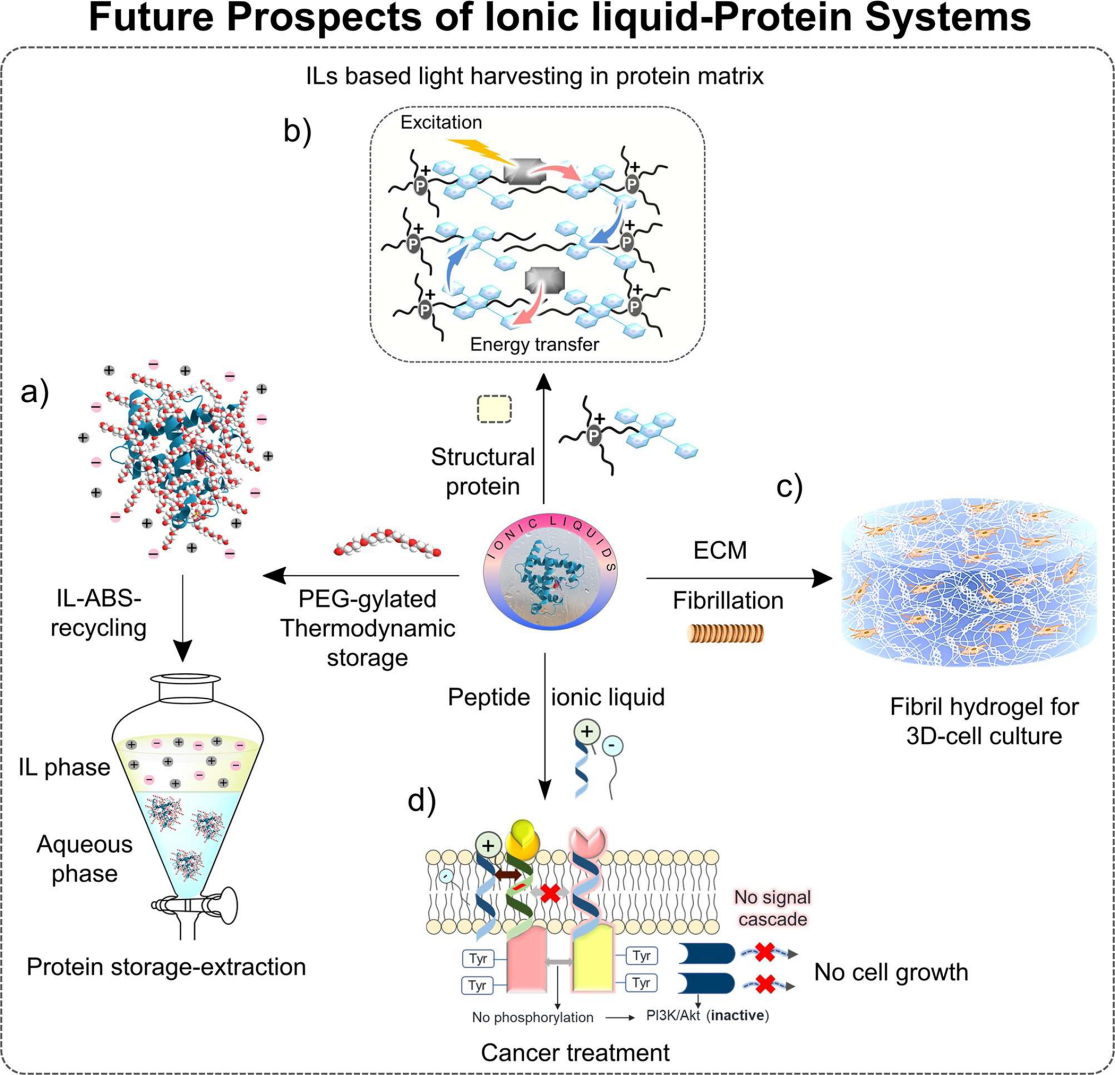
Future prospects of IL-protein systems. (a) Thermodynamic storage and extraction of proteins. (b) Air stable light-harvesting via fluorescent ILs embedded in the protein matrix. (c) Utilization of IL-protein chemistry to prepare fibrils from ECM and further use to create cytocompatible hydrogels for 3D-cell growth. (d) Anticancer membrane peptide–ionic liquids for cancer treatment.

Apart from the discussed possibilities and based on the listed promising systems making use of ILs, IL-protein systems should be investigated for other promising applications. Some identified opportunities are summarized in Figure 30. For thermodynamic packaging of proteins or TPs in dry ILs, the strategy of Brogan et al.^150^ is very useful. Its combination with thermoreversible IL-ABS developed by Passos et al.^158^ would provide a suitable pathway for protein re-extraction. The proposed process can avoid the power consumed during protein storage at low temperatures.

The tuning nature of ILs can further be investigated to introduce fluorescence. The solubilization of fluorescent ILs in the matrix of structural proteins could provide a suitable platform for air-stable exciton energy transfer, like the one observed in the thylakoid membrane during photosynthesis. ILs have been used earlier in light-harvesting by photon upconversion *via* the triplet–triplet annihilation process, which can give direction for the design of a suitable system.^397−399^ The hybrid protein hydrogels prepared using protein fibrils of extracellular matrices should be considered as promising cytocompatible 3D platforms for cell culture. Generally, the synthetic acrylate hydrogels used for 3D cell growth have been found to be toxic;^400^ therefore, developing hydrogels from biological raw material could overcome such issues. Most studies on IL-protein systems have been carried out on single IL-single protein systems, which is not the case for biological systems where proteins are never found alone in the cell, except chlorella. Therefore, future studies, if targeted at the protein of a specific location of the cell, must be done in the presence of other proteins of that cell, i.e., protein crowded medium for biomimicking biological fluids.^401−403^ This would reveal the actual effect of IL-protein and protein-protein interactions when we think of applying them for biochemistry, cell biology, and biotechnology purposes. Furthermore, when studying the effect of ILs and proteins (individual or combined), the effect of the IL cation and anion and their use at different ratios should also be investigated to see the actual relevance of using them in the IL form. We think that these protocols would foster the field with high scientific credibility rather than notoriety.

The biomembrane plays an important role in the signaling growth activities in cells. However, a mutation in the gene responsible for cell growth can result in amplification of cell growth signals due to fast heterodimerization of epidermal growth factor receptors (EGFR), which can cause enhanced cell growth and lead to cancer.^404−406^ This trend can be controlled by stopping the growth signal through transfection of the cell with a competitive synthetic EGFR membrane-active peptide (EGFR-MAB) for fake heterodimerization. Here, membrane peptide labeled surface-active ILs can play a key role because their self-assembled structures can alter the cancer cell membrane either via altering the membrane structure through hydrophobic interactions or by forming fake heterodimers with membrane peptides to stop the cell signaling for malignant growth. Fundamental studies in this direction can lead to significant advances in the treatment of cancer.

Although not considered in this review, the use of ILs to allow the stable immobilization of enzymes on nanomaterials is an emerging field.^407−409^ Enzyme immobilization on nanoscale materials offers valuable advantages in enhancing enzyme stability during challenging reaction conditions. However, current immobilization methods suffer from a significant drawback: irreversible damage caused by the intense interaction between the enzyme and the carrier. To address this issue, the use of ILs becomes crucial as they promote softer interactions, reducing the damage caused during the immobilization process in a solid matrix. By combining enzyme surface modification through nanomaterials and solvent manipulation using ILs, an environmentally friendly approach can be created in the form of protein nanoconstructs for resilient biocatalysis.

## Abbreviations

IL: Ionic Liquid
TPs: Therapeutic proteins
FDA: Food and Drug Administration
NILs: Neat ionic liquids
A-ILCS: Aqueous concentrated-IL solution
A-ILAS: Aqueous adjuvant-IL solution
SAIL: Surface active ionic liquid
IL-ME: Ionic liquid-microemulsion
IL-ABS: IL-aqueous biphasic system
P-P-Conjugate: Poly(ionic liquid)-protein conjugate
P-IL: Protein-ionic liquid
CAL B: Candida antarctica lipase B
BSA: Bovine serum albumin
HSA: Human serum albumin
CRL: *Candida rugosa* lipase
[C_*n*_mim]: Alkyl imidazolium
[C_*n*_OHmim]: Hydroxy alkylimidazolium
[CnPy]: Alkyl pyrolidinium
[CnPr]: Alkylpyridinium
[C_*n*_P]: Alkyl phosphonium
[Cho]: Choline
[C_*n*_N]: Tetraalkyl ammonium
[C_*n*_NH_3_]: Alkyl ammonium
[DEA]: Diethanolammonium
[BAGUA]: Butylpentamethylguanidinium
[BCGUA]: Butyltrimethylguanidinium
[MCGUA]: Tetramethylguanidinium
[DCGUA]: Decyltrimethylguanidinium
[Gdn]: Guanidinium
[Mor]: Morpholinium
[TMG]: Tetramethylguanidinium
[C_3_OHTEA]: Hydroxypropyl triethylammonium
[DMEA]: N-N-dimethylethanolammonium
[OAc]: Acetate
[HCOO]: Formate
[Tf_2_N]: Bis(trifluoromethylsulfonyl)imide
[Sar]: Sarcosinate
[Doc]: Deoxycholate
[TGA]: Thioglycolate
[Pn]: Propionate
[C_*n*_OPO_3_]: Alkylphosphate
[SCN]: Thiocynate
[HSO_4_]: Hydrogen sulfate
[MDEGSO_4_]: 2(2-Methoxyethoxy)ethylsulfate
[Gly]: Glycolate
[Lac]: Lactate
[OH]: Hydroxy
[BF_4_]: Tetrafluoroborate
[Bit]: Bitartarate
[DHCit]: Dihydrogencitrate
[Prop]: Propionate
[But]: Butyrate
[dca]: Dicyanamide
[dhp]: Dihydrogen phosphate
[NO_3_]: Nitrate
[PF_6_]: Hexafluorophosphate
[C_*n*_OSO_3_]: Alkyl sulfate
[TfO]: Trifluoromethanesulfonate
[Asc]: L-Ascorbate
I: Iodide
Cl: Chloride
Br: Bromide
[DBS]: Dodecylbenzenesulfonate
SDS: Sodium dodecyl sulfate
CTAB: Cetyltriethylammonium bromide
HEPES: 2-[4-(2-Hydroxyethyl) piperazin-1-yl]ethanesulfonic acid
TES: 2-[(2-Hydroxy-1,1-bis(hydroxymethyl) ethyl) amino] ethanesulfonic acid
Tricene: N-Tris(hydroxymethyl)methyl] glycine
CHES: 2-(cyclohexylamino)ethanesulfonic acid
MES: 2-(N-Morpholino) ethanesulfonic acid
PEO: Poly(ethylene oxide)
PEG: Polyethylene glycol
